# Analysis of the miR482 Gene Family in Plants

**DOI:** 10.3390/genes15081043

**Published:** 2024-08-08

**Authors:** Wei Kuang, Danfeng Qin, Ying Huang, Yihua Liu, Xue Cao, Meng Xu

**Affiliations:** College of Agriculture and Forestry Sciences, Linyi University, Linyi 276000, China; 202108120106@lyu.edu.cn (W.K.); qindanfeng@lyu.edu.cn (D.Q.); huangying@lyu.edu.cn (Y.H.); liuyihua@lyu.edu.cn (Y.L.); xumeng@lyu.edu.cn (M.X.)

**Keywords:** miR482, molecular characterization, phylogenetic evolution, target identification, ceRNA network

## Abstract

MicroRNA482 (miR482) is a conserved microRNA family in plants, playing critical regulatory roles in different biological activities. Though the members of the miR482 gene family have been identified in plants, a systematic study has not been reported yet. In the present research, 140 mature sequences generated by 106 precursors were used for molecular characterization, phylogenetic analysis, and target gene prediction, and the competing endogenous RNA (ceRNA) network mediated by miR482 was summarized. The length of mature sequences ranged from 17 nt to 25 nt, with 22 nt being the most abundant, and the start and end of the mature sequences had a preference for uracil (U). By sequence multiplex comparison, it was found that the mature sequences of 5p were clustered into one group, and others were clustered into the other group. Phylogenetic analysis revealed that the 140 mature sequences were categorized into six groups. Meanwhile, all the precursor sequences formed a stable hairpin structure, and the 106 precursors were divided into five groups. However, the expression of miR482 varied significantly between different species and tissues. In total, 149 target genes were predicted and their functions focused on single-organism process, cellular process, and cell and cell part. The ceRNA network of miR482 in tomato, cotton, and peanut was summarized based on related publications. In conclusion, this research will provide a foundation for further understanding of the miR482 gene family.

## 1. Introduction

MicroRNAs (miRNAs) are endogenous small non-coding RNAs (ncRNA), 17–25 nts in length, which are widely distributed in both plants and animals [1]. They were first reported in the nematode *Caenorhabditis elegans* as early as 1993 [2]. However, the first miRNAs in plants were reported in 2002, with miRNAs discovered in *Arabidopsis thaliana* [3]. Since then, with the development and application of high-throughput sequencing, more and more miRNAs have been reported in radish [4], wild soybean [5], tomato [6], and so on. So far, miRNAs from 271 species, including nearly 200 plant genomes, have been studied.

miRNAs are regarded as gene expression regulators, which play key roles at the post-transcriptional level by binding to target mRNAs and combining with argonaute proteins to achieve the blockage of mRNA translation or degradation of target mRNAs [7,8]. Additionally, in some cases, miRNA could induce the generation of secondary phased siRNAs (phasiRNAs) from the target genes after the cleavage [9]. miRNAs are involved in various biological processes, ranging from plant growth and development to biotic and abiotic stresses [10,11]. In particular, their function in the responses to different stress has attracted more attention. For example, apple miR160 can improve drought resistance by promoting the development of roots and rhizomes [12]. The knockdown lines of soybean miR166 displays reduced gibberellin content and plant dwarfing [13]. miR398 regulates the expression of target genes (the Cu/Zn superoxide dismutase gene family) to adapt to a variety of adverse stresses [14]. miR482 inhibits the nucleotide-binding site–leucine-rich repeat (*NBS-LRR*) genes, which are involved in resistance to pathogens [15].

Long non-coding RNAs (lncRNAs) and circular RNAs (circRNAs) are other kinds of non-coding RNAs. By interacting with miRNA response elements (MREs), they can act as competing endogenous RNAs (ceRNAs) to regulate the expression of target genes with miRNAs [16,17,18]. This regulatory model reveals the complex ceRNAs network formed by different kinds of non-coding RNAs mediated by miRNAs. Currently, the regulatory mechanism of ceRNAs is a hotspot for further research on miRNAs.

miR482 is a class of miRNAs distributed in various plants. The first plant that miR482 was identified in was *Populus trichocarpa*, while it was absent from Arabidopsis, and it was found that miR482 was conserved between rice and Populus by searching the rice genome [19]. It is worth mentioning that miR2118 has highly similar sequences to miR482, and together with miR482, they are now known as the miR482/2118 superfamily. However, there are differences between their beginning sequences; miR482 expands more widely in dicots while miR2118 expands in monocots, and they have different functions: miR482 is mainly responsible for anti-disease functions, and miR2118 is mainly related to growth and development [20]. Afterward, it was successively reported in soybean [21], apple [22], tomato [23], and other plants. The regulation modes of miR482 vary among different plants, making its function interesting. For example, miR482 in black cottonwood targets the gene encoding a resistance protein [19]. It also directly targets the *NBS-LRR* gene in tomato, engaging in the late blight response process [24]. In cotton, miR482 targets the *NLR* gene, playing a role in the response to dahlia yellow mosaic disease [25]. Additionally, miR482/2118 in litchi cleaves a long non-coding RNA gene, *LcTASL1*, and induces the production of phasiRNAs, which is involved in GA signaling [26]. These findings suggest that miR482 plays a positive role in regulating multiple stress responses [24,27,28]. Therefore, research on the molecular features, evolutionary patterns, target gene functions, and ceRNAs network of the miR482 family in plants is very meaningful, and could lay a theoretical foundation for further understanding of the genetic evolutionary structure and functional studies of the plant miR482 family.

## 2. Materials and Methods

### 2.1. Data Preparation of miR482 Family Members for Bioinformatic Analysis

For the identification of the miR482 family in plants, the mature and precursor sequences of the miR482 gene family in plants were respectively downloaded from the miRBase database server v. 22.0 (http://www.mirbase.org/, accessed on 10 August 2023) [29]. The sequences were used for the following bioinformatic analysis.

### 2.2. Molecular Characterization of miR482 Family Members

The mature and precursor sequences of the miR482 family were classified and analyzed, respectively. Based on the mature sequences, the sequences were divided into two groups, with all the 5p sequences forming one group and the rest forming the other. The two groups were analyzed for base preference separately on the MEME website (https://meme-suite.org/meme/tools/meme, accessed on 4 July 2024) [30]. According to the minimum number of mature bases, a minimum of 17 and 19 motifs were set for base preference analysis, and other settings according to system defaults. Meanwhile, the precursors were submitted to the RNAfold website (http://rna.tbi.univie.ac.at//cgi-bin/RNAWebSuite/RNAfold.cgi, accessed on 22 August 2023) for secondary structure prediction.

### 2.3. Sequence Alignment and Phylogenetic Tree Estimation

The multiple alignment of the miR482 gene family was conducted by MEGA software (version 11) using the ClustalW method. And MEGA software was utilized to align mature and precursor sequences of the plant miR482 family, and then to construct the phylogenetic tree based on the neighbor-joining (NJ) method [31]. Ultrafast bootstrapping was carried out 1000 times, and other parameters were set to default [32].

### 2.4. Expression Profiling of miR482

To analyze the expression level of miR482 in different tissues of different species, the expression data of miR482 in *Citrus sinensis*, *Nicotiana tabacum*, *Solanum lycopersicum*, *Solanum tuberosum*, *Fragaria vesca*, *Glycine max*, *Manihot esculenta*, and *Prunus persica* were obtained from PmiREN (Plant miRNA ENcyclopedia) and used for comparison.

### 2.5. Target Gene Prediction and GO Enrichment Analysis

The plant small RNA target analysis server tool (psRNATarget, https://www.zhaolab.org/psRNATarget/home, accessed on 24 August 2023) was employed to predict the potential target genes of miR482 in plants [33]. The analysis was conducted under a strict setting where the maximum expected value was set to 1.5. Then, the National Center for Biotechnology Information (NCBI, https://www.ncbi.nlm.nih.gov/, accessed on 28 August 2023) was used for an online comparison to obtain the gene function annotation. The EGGNOG-MAPPER website (http://eggnog-mapper.embl.de, accessed on 4 July 2024) was employed for the GO annotation of predicted target genes. And the results of the GO annotation were visualized using the online omicshare tool (https://www.omicshare.com/tools/Home/Soft/gogseasenior, accessed on 4 July 2024).

### 2.6. CeRNA Network Construction

To further analyze the function of plant miR482, we systematically summarized related research on the regulation of miR482 by ncRNAs described in the current literature. The ceRNA network of miR482 was based on the following publications: Liu et al. [34], Liu et al. [35], Xu et al. [36], Si et al. [37], and Li et al. [38]. And the ceRNA network of miR482 was constructed using Cytoscape (version 3.9.1).

## 3. Results

### 3.1. Statistical Analysis of Members of the miR482 Family in Plants

**Figure 1:**
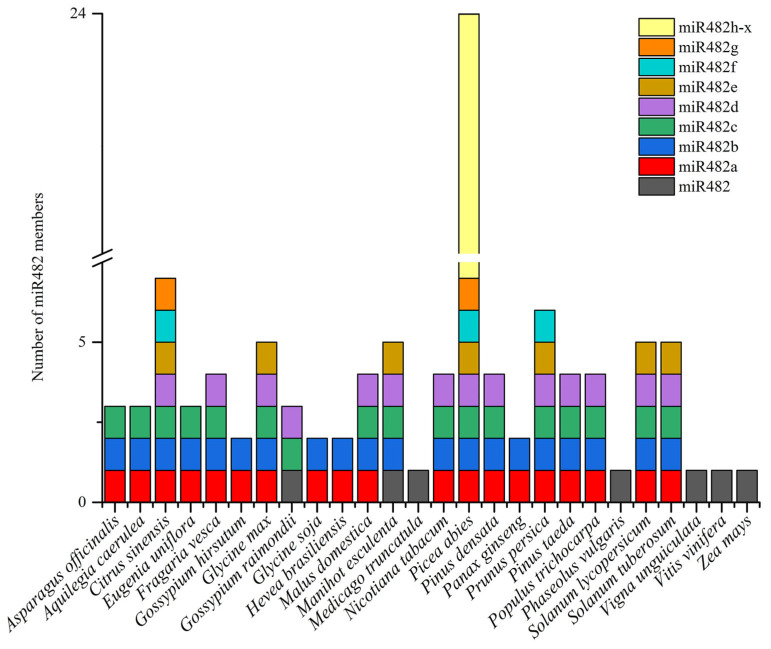
Presence and distribution of the miR482 family in plants.

During the accession into the miRbase database, the members of the miR482 family in plants were downloaded (Table S1). In total 140 mature sequences from 26 species belonging to 14 families were found, as shown in Table 1 and Figure 1. The largest miR482 family was *Picea abies* with 25 members including 24 different submembers ranging from miR482a to miR482x, followed by *C. sinensis* with 14, including 7 different submembers, while only 1 member was in *Medicago truncatula*, *Phaseolus vulgaris*, *Vigna unguiculata*, *Vitis vinifera*, and *Zea mays*. Among these sequences, 99 mature sequences of miR482 were located at the 3′ end of the precursor, while 41 mature sequences were at the 5′ end. Meanwhile, 91 mature sequences were experimentally verified, 8 mature sequences were obtained by homologous comparison, and 41 mature sequences remained to be verified.

### 3.2. Molecular Characterization of the Mature and Precursor Sequences of the miR482 Family

**Figure 2:**
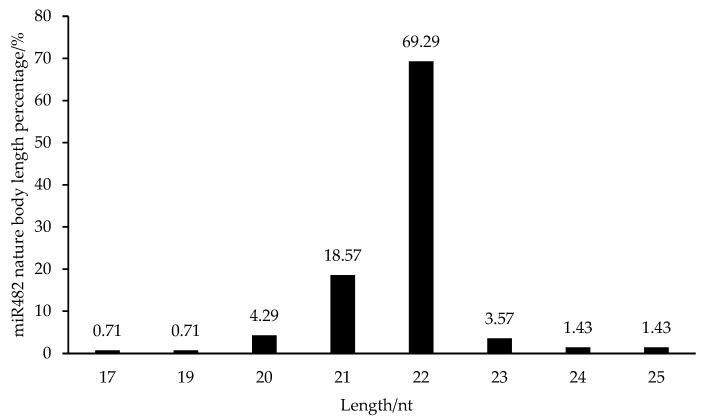
Length distribution of the miR482 family in plants.

The distribution of the length of mature sequences in the miR482 family is shown in Figure 2. The length of miR482 mature sequences was mainly within 20–23 nt. And the sequences of 22 nt in length accounted for 69.29% and were the most abundant, which is basically in line with the typical miRNA maturation length characteristics of plants.

**Figure 3:**
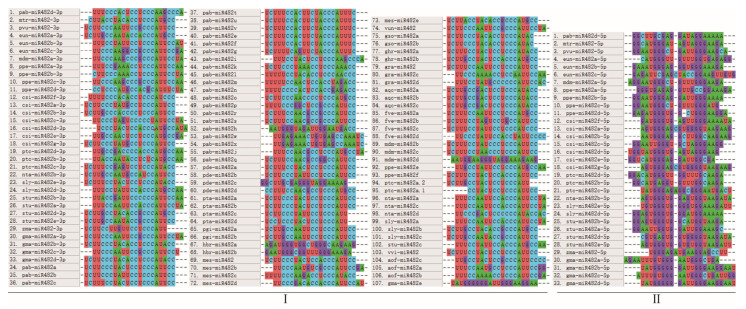
Multiple-sequence comparison of mature sequences in the miR482 family: (**I**) comparison of the 3p and non 5p or 3p miR482 sequence; (**II**) comparison of the 5p sequences.

A sequence multiplex comparison of the mature sequences is shown in Figure 3. Most mature sequences were highly conserved. These sequences could be categorized into two groups based on base characterization and conservatism, where the mature sequences produced at the 5′ end were clustered into group II and the rest were clustered into group I.

**Figure 4:**
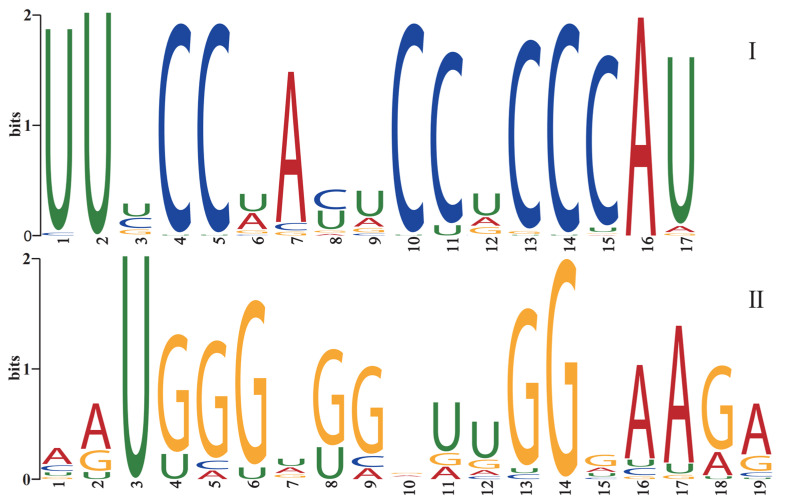
Base preference analysis of the mature sequences in the miR482 family. (**I**) 3p sequences and non 5p or 3p miR482 sequence were analyzed for base preference as a group; (**II**) 5p sequences were analyzed for base preference as a group.

The 140 mature sequences of the miR482 family in plants were divided into two groups utilized for base preference analysis at each position, and the results are shown in Figure 4. In group I, a preference for uracil (U) was observed in the 1st, 2nd, and 17th positions, whereas the 4th, 5th, 10th, 11th, 13th, 14th, and 15th positions showed a preference for cytosine (C). In group II, a preference for uracil (U) was observed only in the 3rd position, whereas the 4th, 5th, 6th, 8th, 13th, 14th, and 18th positions showed a preference for guanine (G).

Based on the precursor sequences of the miR482 family, the secondary structure of the precursor sequences was predicted via the RNAfold website. All the precursor sequences of the miR482 family could form the stable hairpin structure (Figure S1). The number of loop structures formed by each precursor was not the same, the miR482 mature sequences could be generated from either 5p or 3p, and the mature sequences might also include some of the loop sequences and stem sequences.

### 3.3. Phylogenetic Analysis of the miR482 Family in Plants

**Figure 5:**
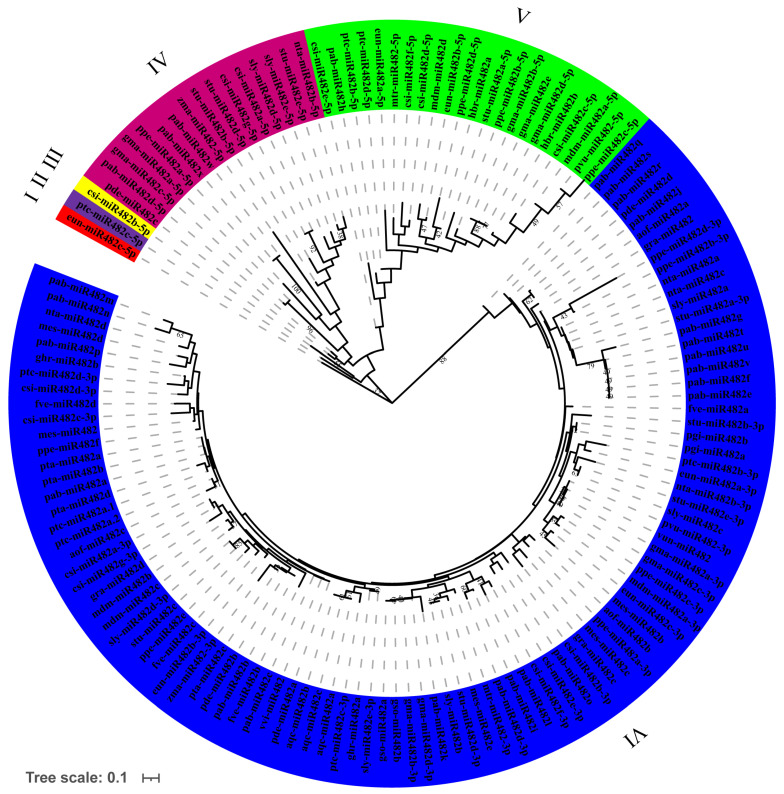
Phylogenetic relationship and classification of the mature sequences from the plant miR482 family.

To explore the evolutionary relationships of the miR482 family members in plants, a phylogenetic tree based on the alignments of 140 mature sequences was constructed (Figure 5). All the mature sequences could be divided into six groups, from group I to group VI. Group VI (Figure 5, blue branches) contained the most mature sequences, including 99 mature sequences of the miR482 family. Groups IV and V contained 16 and 22 sequences, respectively. And only 1 sequence was in groups I, II, and III, respectively. All miR482-3p mature sequences were clustered into group VI, while miR482-5p mature sequences were widely distributed in other groups.

**Figure 6:**
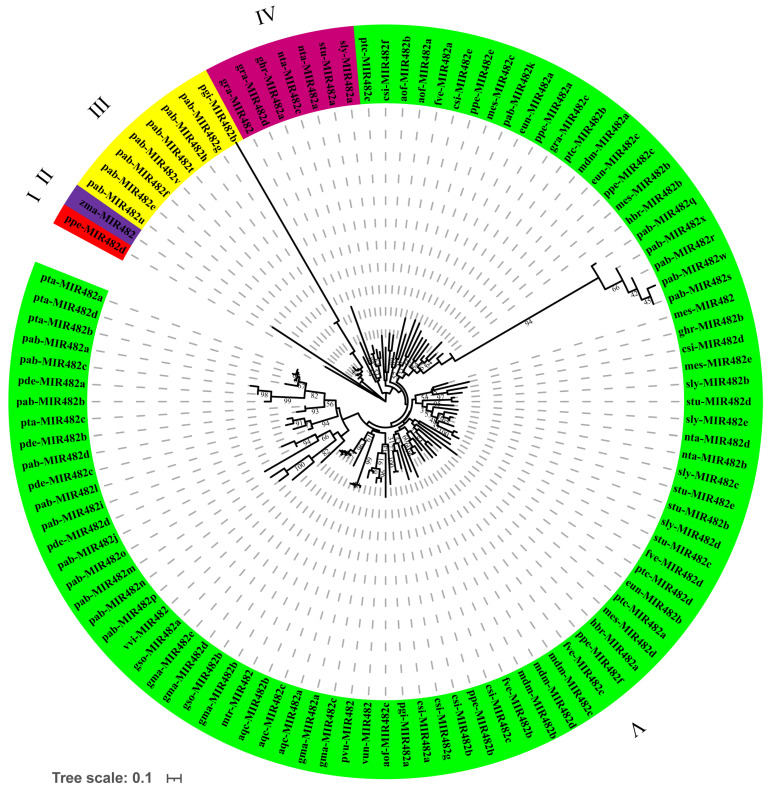
Phylogenetic relationship and classification of the precursor sequences from the plant miR482 family.

Meanwhile, phylogenetic analysis was also performed based on the 106 precursor sequences of the plant miR482 family, and the results are shown in Figure 6. Different from the phylogenetic tree of mature sequences, all the precursors were divided into five groups, from group I to group V. Of these, group V contained the largest number of precursor sequences, including 89 precursor sequences (Figure 6, green branches). Groups III and IV contained eight and seven sequences, respectively. Groups I and II included only one precursor sequence.

### 3.4. Tissue-Specific Expression Analysis of miR482

**Figure 7:**
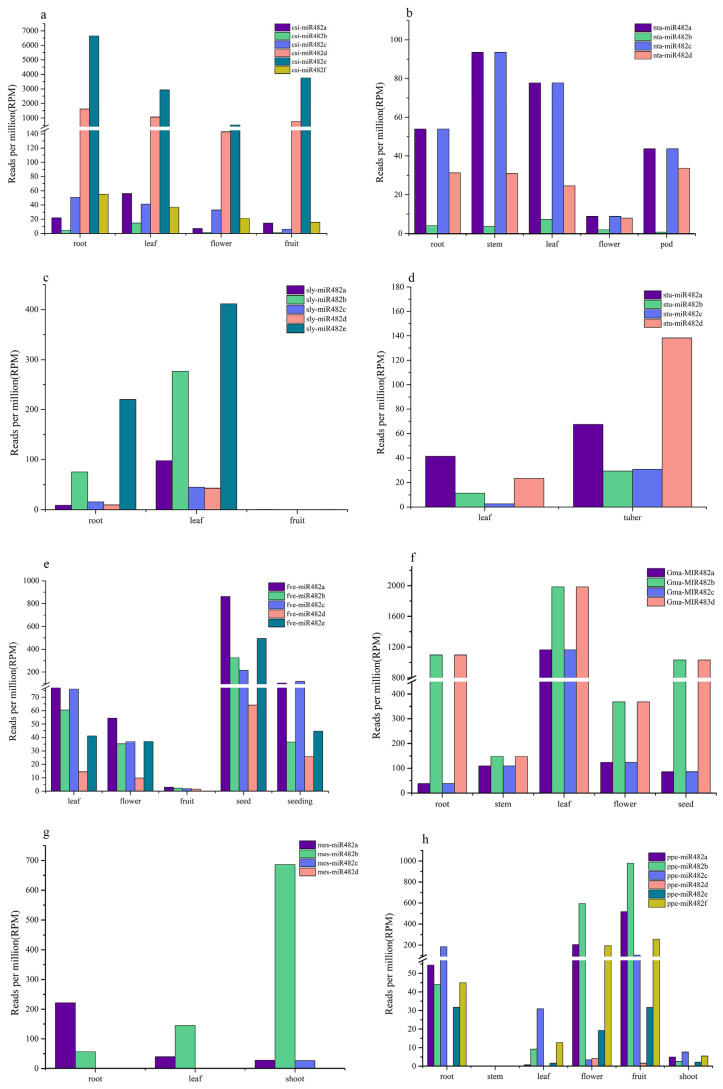
Expression patterns of miR482 in *C. sinensis* (**a**), *N. tabacum* (**b**), *S. lycopersicum* (**c**), *S. tuberosum* (**d**), *F. vesca* (**e**), *G. max* (**f**), *M. esculenta* (**g**), and *P. persica* (**h**). miR482a, miR482b, miR482c, miR482d, miR482e, and miR482f are the members of the miR482 family.

Based on the expression information obtained from PmiREN, the tissue-specific expression patterns of miR482 in *C. sinensis*, *N. tabacum*, *S. lycopersicum*, *S. tuberosum*, *F. vesca*, *G. max*, *M. esculenta*, and *P. persica* were analyzed (Figure 7). As expected, the expression of miR482 varied significantly between different species and tissues. miR482 was highly expressed in *C. sinensis*, *F. vesca*, *G. max*, *M. esculenta*, and *P. persica*. A low expression level of miR482 was found in *N. tabacum*, *S. lycopersicum*, and *S. tuberosum*. And the expression differences among different members of miR482 in the same species were also large. In *C. sinensis*, the expression level of miR482e in root was 6646 reads per million (RPM), while the expression level of miR482b in root was only 4 RPM. Significant differences also existed in other species. Compared to other members, miR482a was highly expressed in *N. tabacum* and *F. vesca*, miR482e was highly expressed in *C. sinensis* and *S. lycopersicum*, miR482d was highly expressed in *S. tuberosum* and *G. max*, while miR482b was highly expressed in *M. esculenta* and *P. persica*.

### 3.5. Prediction and Function Annotation of the Targets

To further understand the function of miR482, target gene prediction was performed (Table S2). Of these 140 mature sequences, 149 targets from 63 mature sequences were found across 16 species. miR482 inhibits target genes mainly by cleavage. And multiple members of the miR482 family could target the same gene; for example, fve-miR482a and fve-miR482c co-targeted *Garcinia cambogia* subspecies sphingosine-like kinase 1 (gene30692-v2.0.a2-hybrid). The 3p and 5p of the miR482 produced from the same precursor also targeted the same gene. The sweet orange csi-miR482f-5p and csi-miR482f-3p co-targeted the citrus α-glucan water bisphosphatase 2 gene (DSWO2A02249).

**Figure 8:**
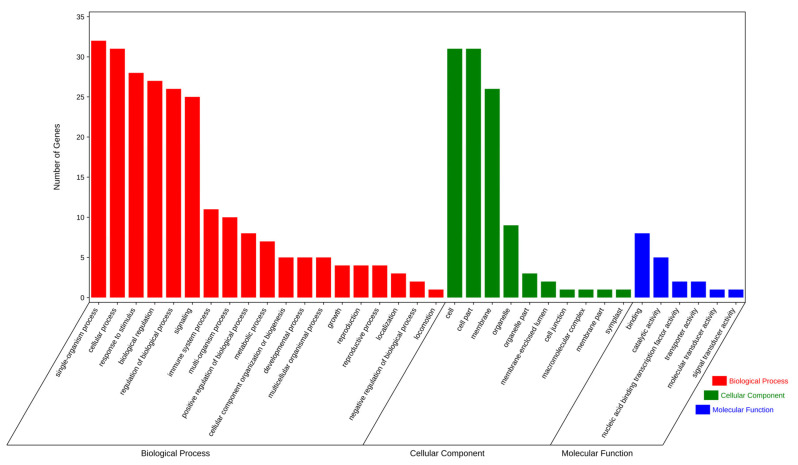
GO enrichment of predicted miR482-targeted genes.

And the functional annotation and GO enrichment analysis of the target genes were conducted using the online website EGGNOG-MAPPER at the same time (Figure 8). The most enriched GO terms of those target genes were in the biological process category, which comprised single-organism process and cellular process. Within the cellular component category, cell and cell part was enriched. And, in the molecular function category, binding was enriched.

### 3.6. CeRNA Network of miR482

**Figure 9:**
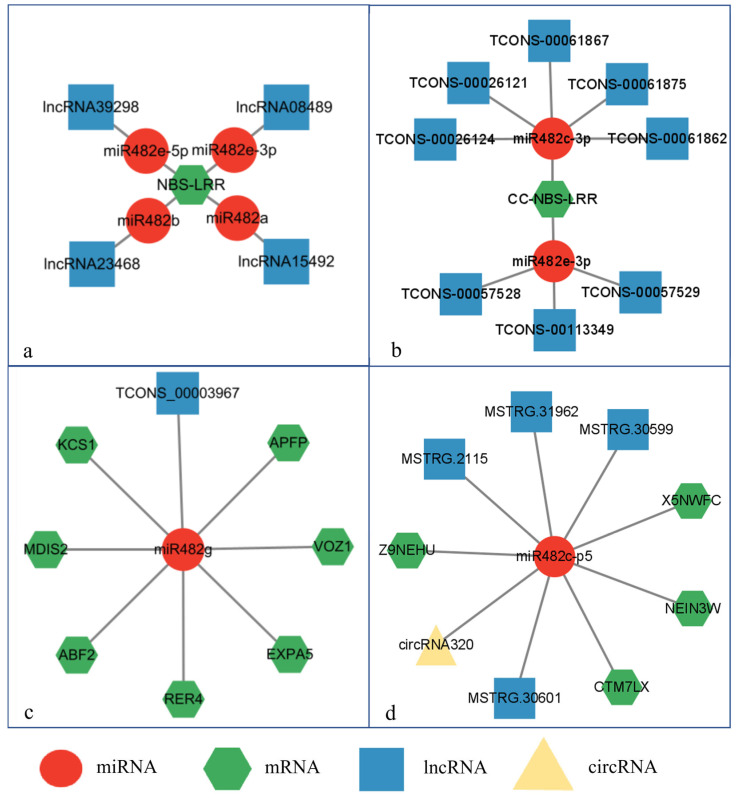
The ceRNAs network of miR482 in plants: (**a**) tomato in response to *P. infestans*; (**b**) tomato in response to *R. solanacearum*; (**c**) cotton in response to drought stress; (**d**) peanut in response to root-knot nematodes.

By integrating the existing related reports, the ceRNA network of miR482 was constructed (Figure 9, Table S3). Tomato sllncRNA15492 and sllncRNA23468 acted as the ceRNAs for miR482a and miR482b, respectively, by regulating *NBS-LRR* in response to *Phytophthora infestans* infection (Figure 9a). In addition, sllncRNA08489 and sllncRNA39298 could also act as ceRNAs to decoy slmiR482e-3p and slmiR482e-5p, respectively, in response to tomato *P. infestans*, which further regulated the expression of *NBS-LRR* (Figure 9a). Furthermore, *CC-NBS-LRR* was found to be co-regulated by miR482c-3p and miR482e-3p, which were the targets of five lncRNAs (TCONS-00026121, TCONS-00061862, TCONS-00026124, TCONS-00061875, TCONS-00061867) and three lncRNAs (TCONS-00057529, TCONS-00113349, TCONS-00057528), respectively, in responding to *Ralstonia solanacearum* (Figure 9b). And the specific lncRNA (TCONS_00003967)–miR482g–mRNA regulatory network was found in cotton response to drought stress (Figure 9c). Additionally, when peanut was infected by root-knot nematodes, four lncRNAs (MSTRG.2115, MSTRG.30601, MSTRG.30599, and MSTRG.31962) and a circRNA (circRNA320) acted as the ceRNAs of miR482c (Figure 9d).

## 4. Discussion

miR482 is widely present in seed plants, and has been reported in a variety of plants including tomato, cotton, and apple [24,39,40]. In this study, 140 mature sequences of the miR482 family from 26 species were obtained and used for analysis. More conserved miRNA families usually have more members [41]. For example, 14 members of the miR482 family were discovered in cotton involved in root-knot nematode *Meloidogyne incognita* infection [42], and there were six members of miR482 in *Chrysanthemums* under salt stress [41]. In this research, 14 out of 26 species had more than three members of the miR482 family, and in particular, *P. abies* contained 25 members, including 24 different submembers ranging from miR482a to miR482x. The expression of miR482 varied significantly between different species and tissues, and the expression of different members of miR482 in the same species also varied widely. For example, miR482e showed a high expression level in *C. sinensis*, while a low expression level in *P. persica*. In *F. vesca*, miR482e had a higher expression level in seed; however, it was not detected in fruit. Many miRNAs are conserved among plants. In our study, 140 mature sequences of the miR482 family were used for analysis. The sequence analysis showed that they were conserved, and the preference for uracil (U) was observed in the start and end bases of the sequences.

According to the phylogenetic tree, all the precursor sequences of the miR482 family were clustered into five groups, and the mature sequences were divided into six groups. The diverse distributional features were independent of the species itself in terms of their affinity and evolutionary proximity (Figure 5 and Figure 6). This is consistent with previous reports of the evolutionary analysis of miR156 [43]. The miR482 precursor sequences of the same species were distributed in different groups. The precursor sequences of miR482 in *P. abies* (pab-MIR482) were clustered in groups III and V, while the mature sequences of miR482 in *P. abies* were clustered in groups IV, V, and VI. This indicates the non-synchronization of the evolutionary rate of different miR482 family member sequences within the same species. All the precursor sequences of miR482 in *A. officinalis* were clustered in group V. Correspondingly, all the mature sequences were gathered in group VI in *A. officinalis*, as in *Pinus taeda*, which indicates that the sequences of miR482 in *A. officinalis* and *P. taeda* have recently diversified. Besides this, csi-miR482b-5p independently formed group III, and the expression levels of csi-miR482b in *C. sinensis* were significantly lower than those of other members. It is hypothesized that there may be some correlation between differences in expression and evolutionary branching.

Previous studies have found that the length of an miRNA mature sequence varies among different species. The lengths of miRNA in poplar [44], ginseng [45], and cotton [46] were at most 21 nt, whereas those of blueberry [47], sweet potato [48], and tea tree [49] were at most 24 nt. And it was reported that the length was related to different functions. miRNAs 21–22 nt in length are mainly associated with mRNA cleavage and gene silencing at the post-transcriptional level [50], while miRNAs 24 nt in length are mainly related to RNA-guided DNA methylation [51]. In this study, the length of miR482 members in plants was at most 21–22 nt, and the mode of regulation between miRNA and target genes was mainly cleavage, which was consistent with our above conclusion.

miR482 is involved in the regulation of stress-related biological activities and mainly regulates *NBS-LRR* genes [52]. For example, apple miR482 targets *NBS-LRR* in response to *Alternaria alternata* f. sp. *mali* infection [40]. In tomato, miR482a, miR482b, miR482e-3p, and miR482e-5p were all involved in the regulation of *P. infestans* infection by targeting *NBS-LRR*, while lncRNA15492, lncRNA23468, lncRNA08489, and lncRNA39298 acted as their target ceRNAs [24,34,35,52]. Additionally, infected with *R. solanacearum*, miR482c-3p and miR482e-3p targeting *CC-NBS-LRR* were both responsive, and eight lncRNAs acted as the target ceRNAs [37]. Then, a ceRNA network was constructed (Figure 9a,b). Of these, the target genes of miR482, *NBS-LRR* genes, are the largest family of resistance genes (R genes) and are widely reported in plants. Also, a similar regulation ceRNA network was found in cotton in response to drought stress, and in peanut after infection with root-knot nematodes (Figure 9c,d). Apart from *NBS-LRR*, the predicted target genes of miR482 were also found to participate in response to stimulus, binding, and cellular anatomical entity. Lipoxygenases (LOXs) are dioxygenases without heme and iron and are involved in the development and adaptation of many plants to the environment. Strawberry *LOX* genes are involved in low-temperature, drought, salt, and other abiotic stress responses [53]. In our research, *LOXs* were targeted by fve-miR482b and fve-miR482c. α-glucan water dikinases are cytosolic enzymes that are important for proper *A. thaliana* seed development and involved in cold tolerance [54,55], which was the predicted target gene of csi-miR482b-5p and csi-miR482b-3p. Taken together, the miR482 gene family plays a critical role in various biological processes. However, the role of the miR482 gene family remains to be further confirmed by experiments.

## 5. Conclusions

In this study, the miR482 gene family members, corresponding predicted target genes, and the ceRNA network in plants were comprehensively analyzed and summarized. This research offers an extensive characterization of miR482 in plants, and also provides valuable references for the study of miR482-mediated plant responses to adversity stress.

## Figures and Tables

**Table 1:** Statistics of the miR482 family in plants.

Families	Species	Name	Number of 5′ End	Number of 3′ End	Evidence
Liliaceae	*Asparagus officinalis*	aof-miR482	0	3	3 (experimental)
Myrtaceae	*Aquilegia caerulea*	aqc-miR482	0	3	0
Rutaceae	*C. sinensis*	csi-miR482	7	7	14 (experimental)
Myrtaceae	*Eugenia uniflora*	eun-miR482	3	3	6 (experimental)
Rosaceae	*F. vesca*	fve-miR482	0	4	4 (experimental)
Malvaceae	*Gossypium hirsutum*	ghr-miR482	0	2	2 (experimental)
Fabaceae	*G. max*	gma-miR482	4	5	9 (experimental)
Malvaceae	*Gossypium raimondii*	gra-miR482	0	3	3 (experimental)
Fabaceae	*Glycine soja*	gso-miR482	0	2	2 (experimental)
Euphorbiaceae	*Hevea brasiliensis*	hbr-miR482	2	0	2 (experimental)
Rosaceae	*Malus domestica*	mdm-miR482	1	4	4 (experimental)
Euphorbiaceae	*M. esculenta*	mes-miR482	0	5	4 (experimental)
Fabaceae	*M. truncatula*	mtr-miR482	1	1	2 (experimental)
Solanaceae	*N. tabacum*	nta-miR482	1	4	3 (experimental)
Pinaceae	*P. abies*	pab-miR482	1	24	25 (experimental)
Pinaceae	*Pinus densata*	pde-miR482	0	4	4 (experimental)
Araliaceae	*Panax ginseng*	pgi-miR482	1	1	2 (experimental)
Rosaceae	*P. persica*	ppe-miR482	4	6	10 (experimental)
Pinaceae	*Pinus taeda*	pta-miR482	0	4	4 (by similarity)
Salicaceae	*P. trichocarpa*	ptc-miR482	3	5	8 (experimental)
Fabaceae	*P. vulgaris*	pvu-miR482	1	1	2 (experimental)
Solanaceae	*S. lycopersicum*	sly-miR482	2	5	5 (experimental)
Solanaceae	*Solanum tuberosum*	stu-miR482	4	5	8 (experimental)
Fabaceae	*V. unguiculata*	vun-miR482	0	1	0
Vitaceae	*V. vinifera*	vvi-miR482	0	1	1 (experimental)
Poaceae	*Z. mays*	zma-miR482	1	1	0

## Data Availability

The original contributions presented in the study are included in the article/Supplementary Materials. Further inquiries can be directed to the corresponding author.

## Supplementary Materials

The following supporting information can be downloaded at https://www.mdpi.com/article/10.3390/genes15081043/s1. Figure S1: Secondary structure of miR482 precursor sequences in plants. Table S1: The mature and precursor sequences of the miR482 family in plants. Table S2: Target genes of miR482 and function annotation. Table S3: ceRNA network of miR482.
