# Supplementary material for: Analysis of the miR482 Gene Family in Plants
Source: Genes (Basel). 2024 Aug 8;15(8):1043. doi: 10.3390/genes15081043 (PMC11353999; doi:10.3390/genes15081043)
Supplement: Supplementary file 1 [file genes-15-01043-s001.zip › Supplementary Materials/Supplementary Materials-Figure S1.pdf]

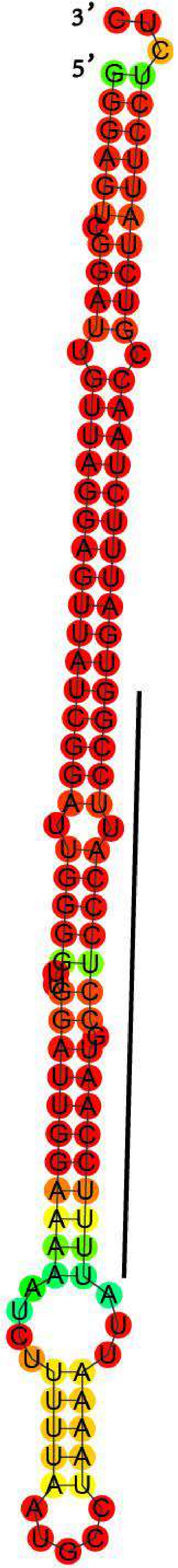

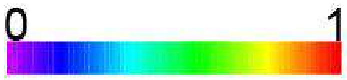

aof-MIR482a

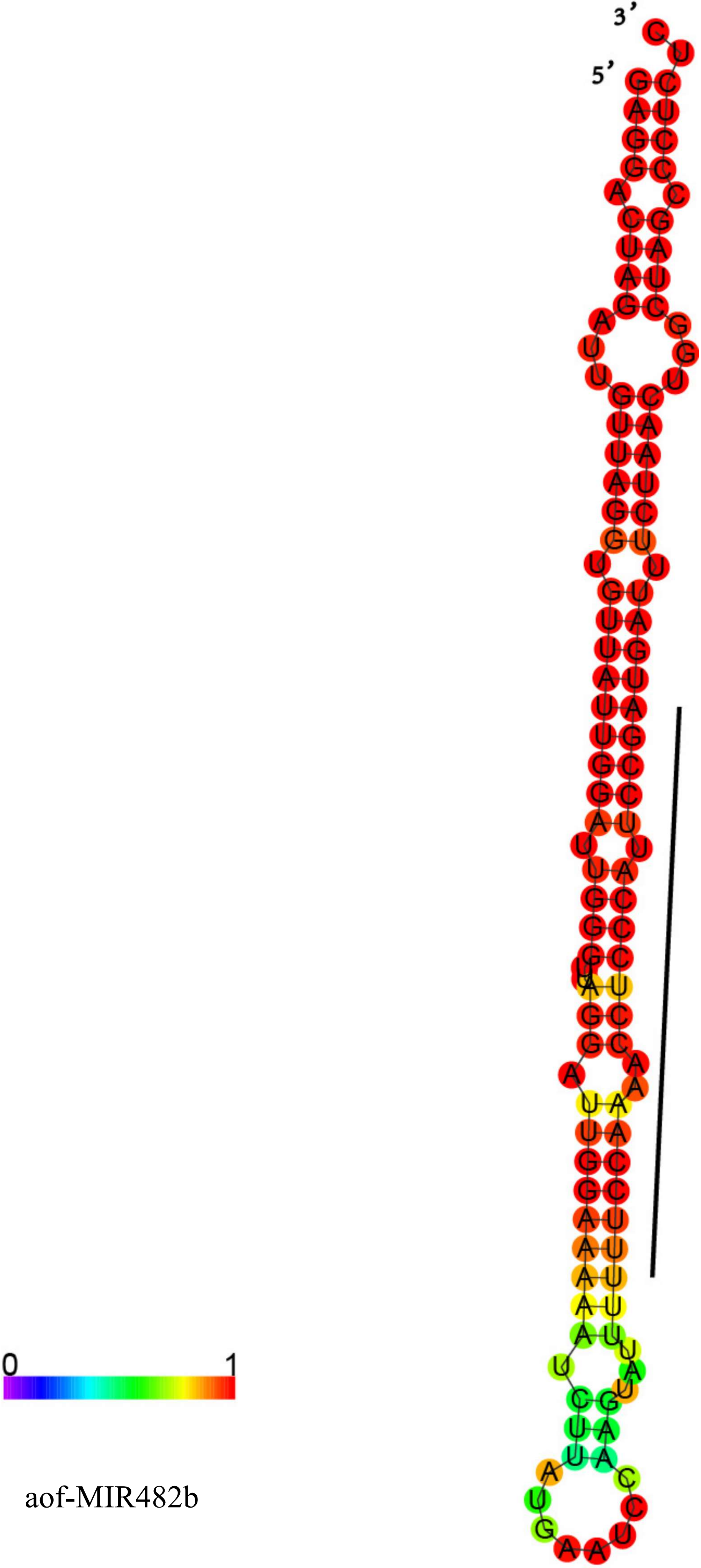

aof-MIR482b

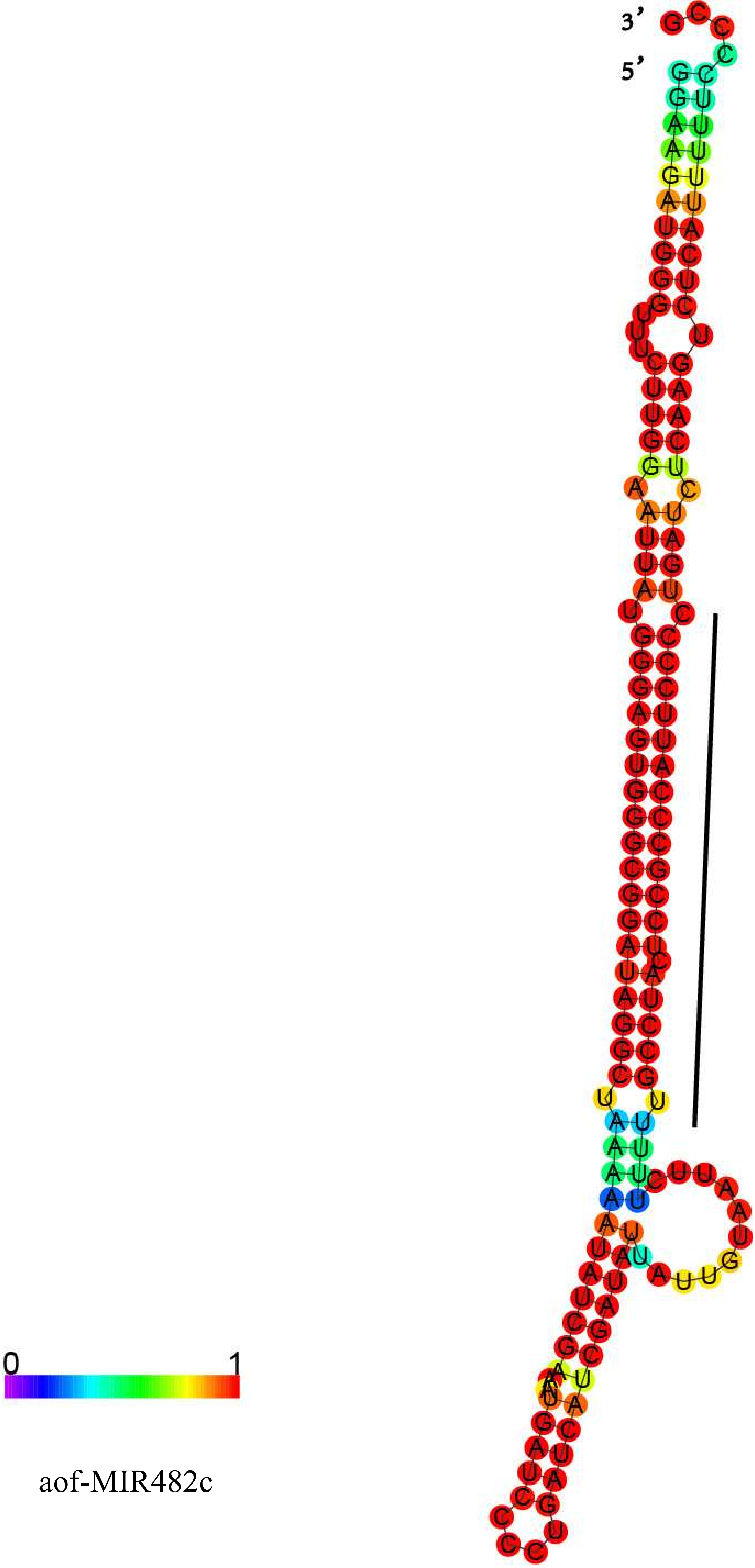

aof-MIR482c

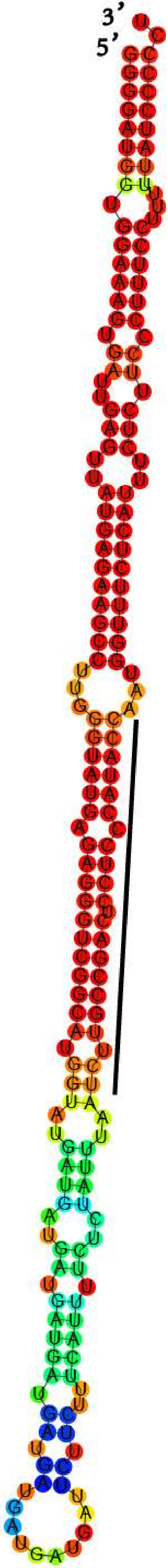

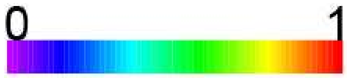

aqc-MIR482a

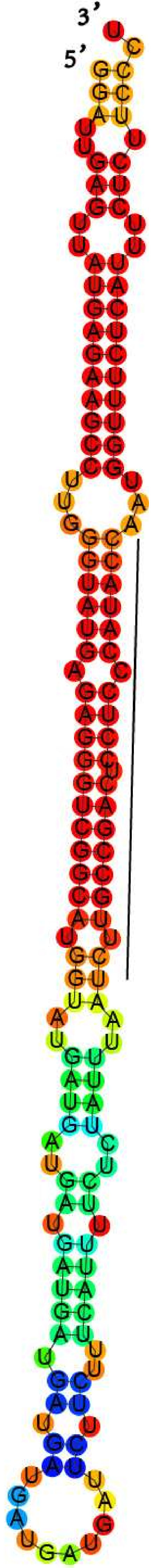

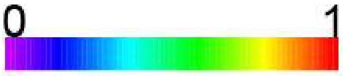

aqc-MIR482b

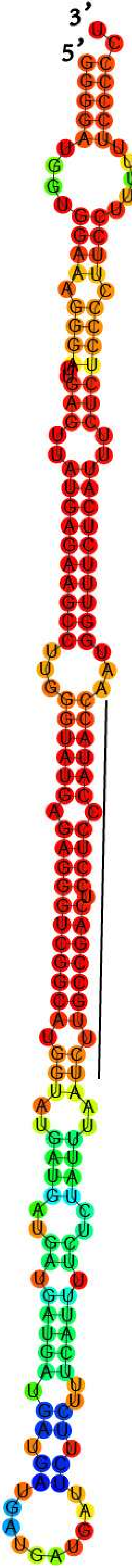

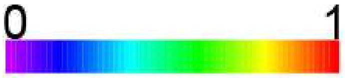

aqc-MIR482c

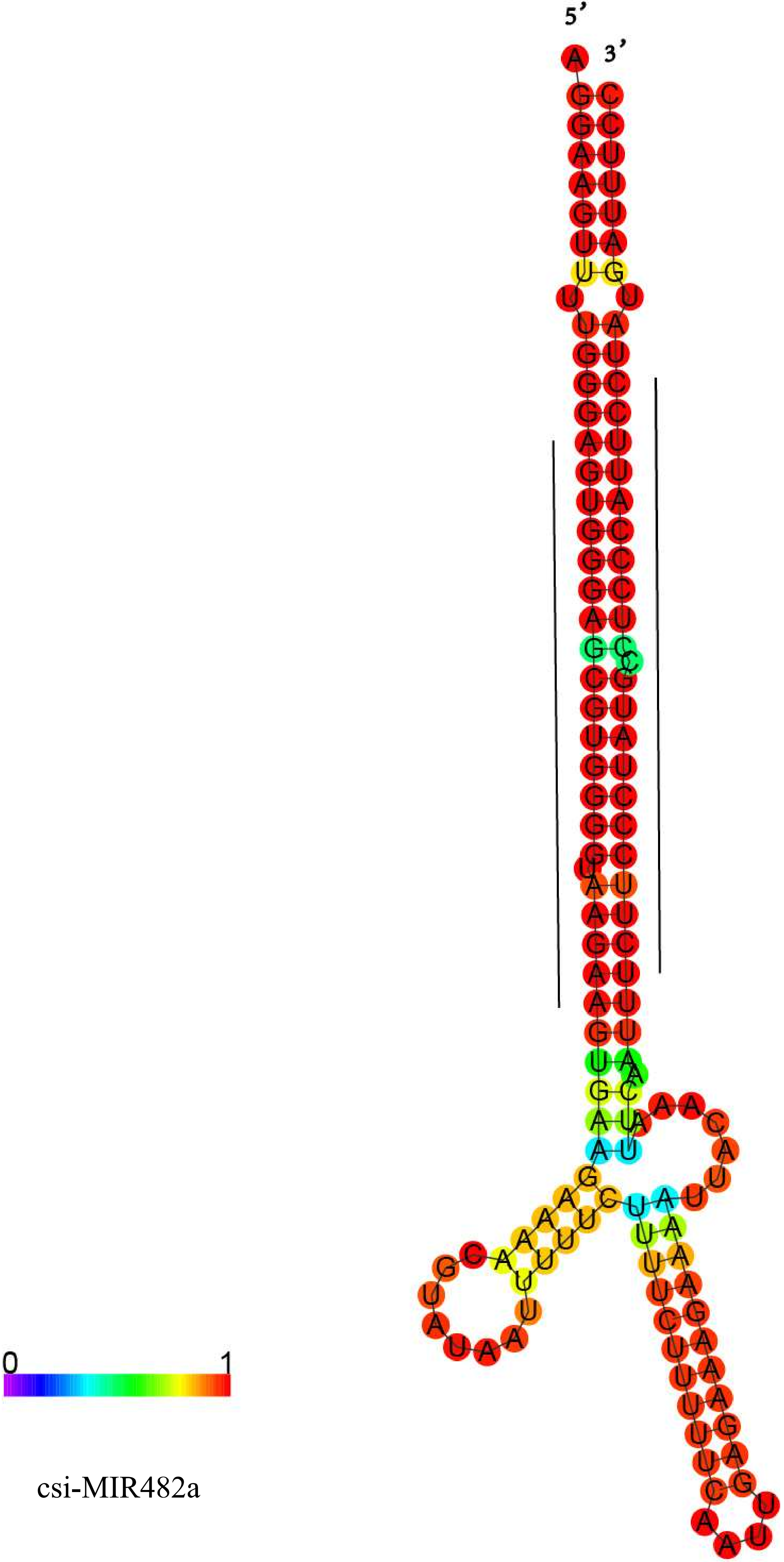

csi-MIR482a

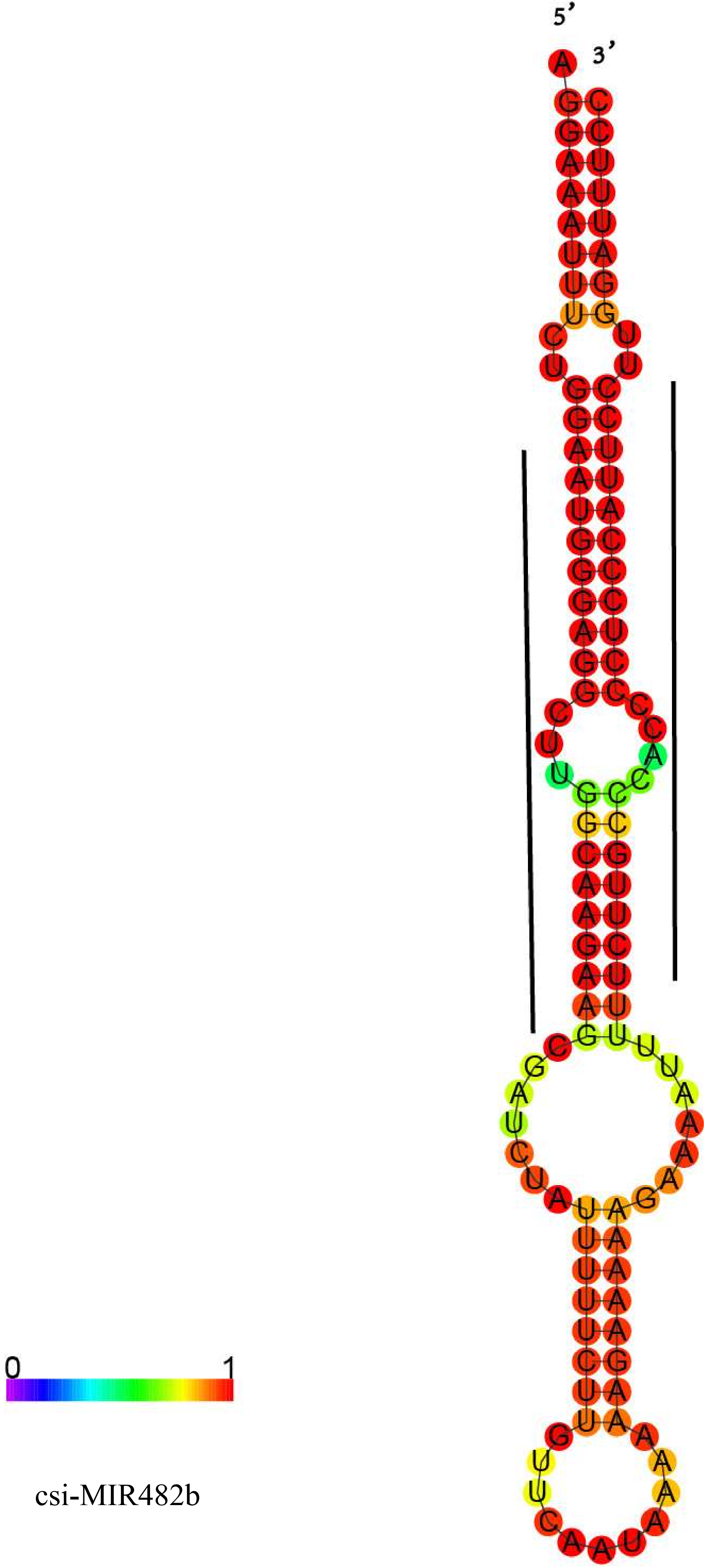

csi-MIR482b

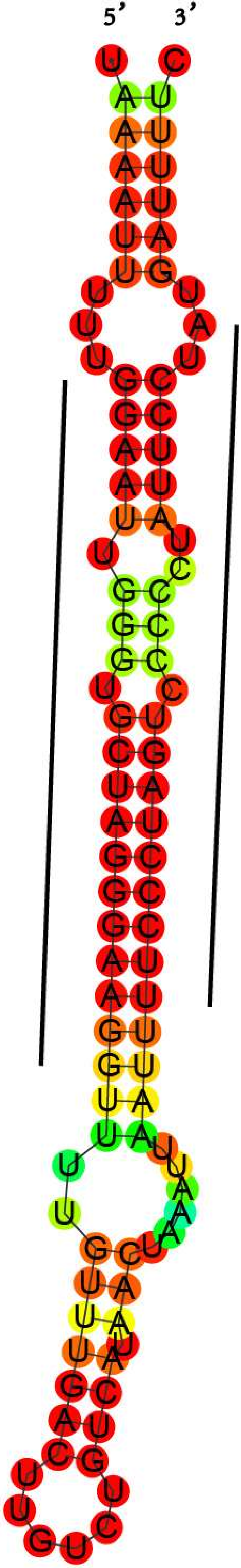

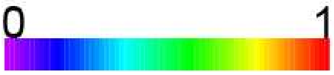

csi-MIR482c

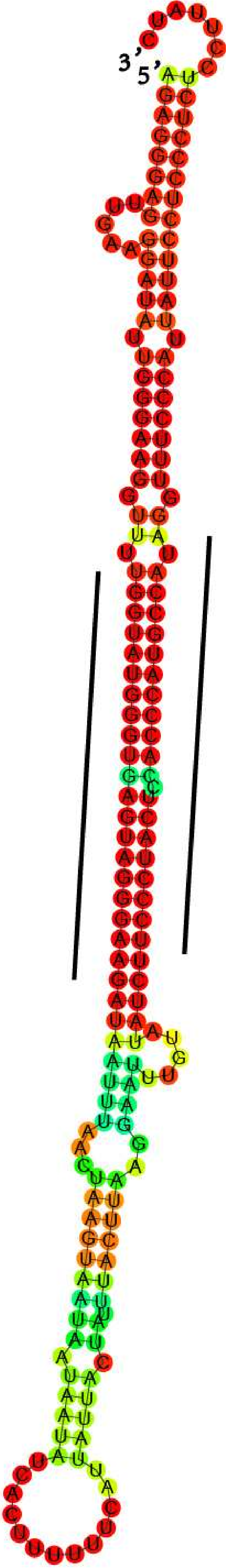

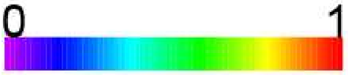

csi-MIR482d

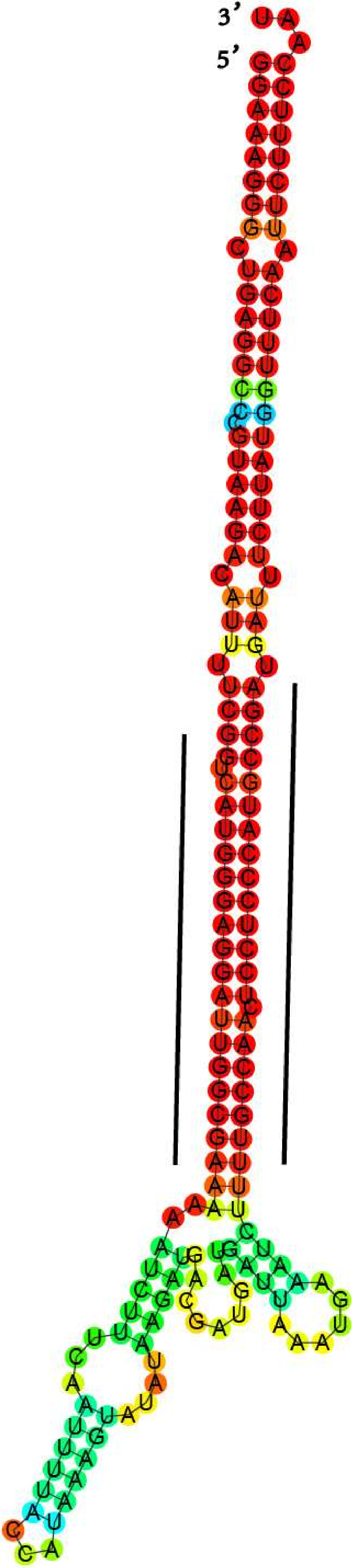

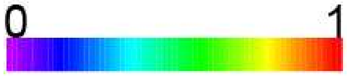

csi-MIR482e

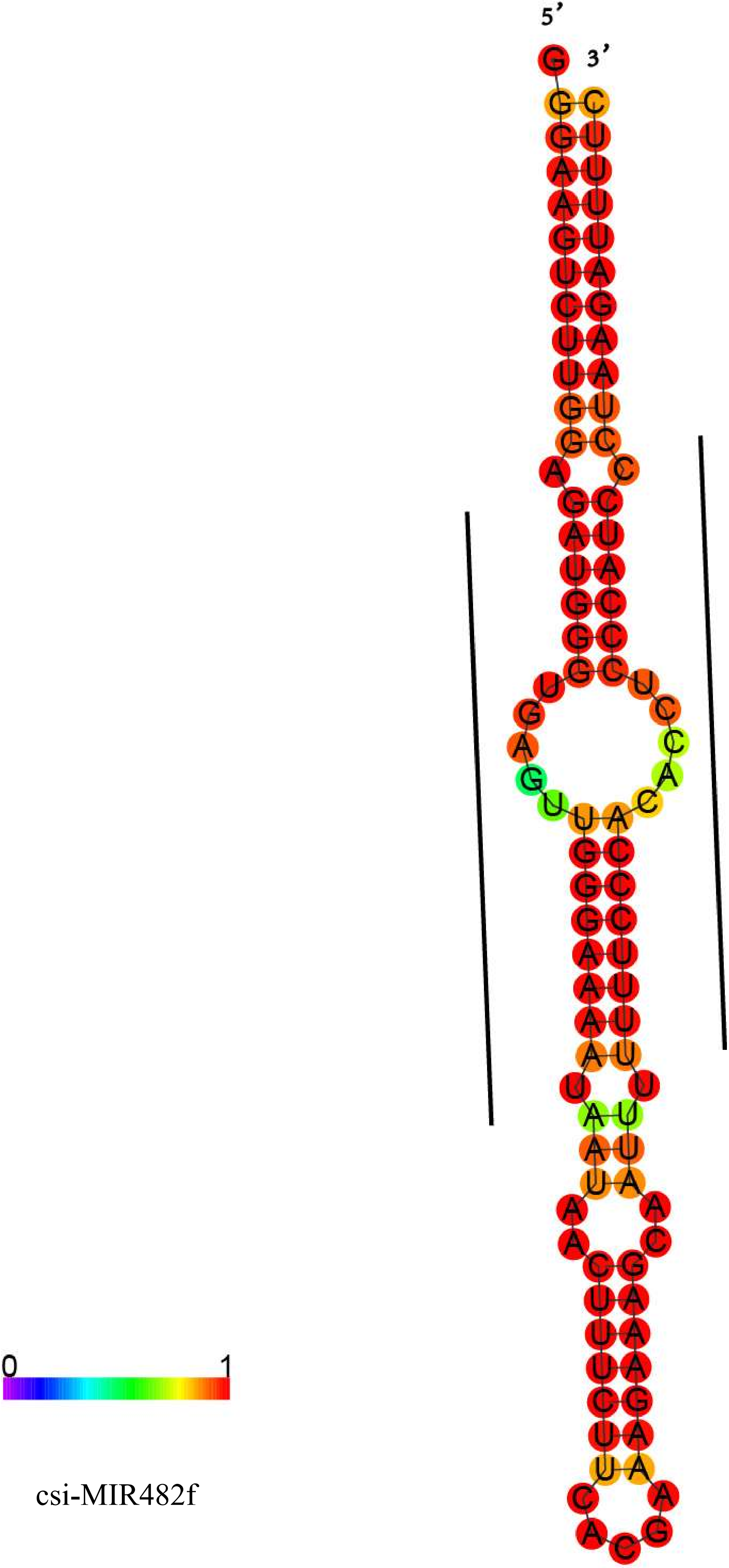

csi-MIR482f

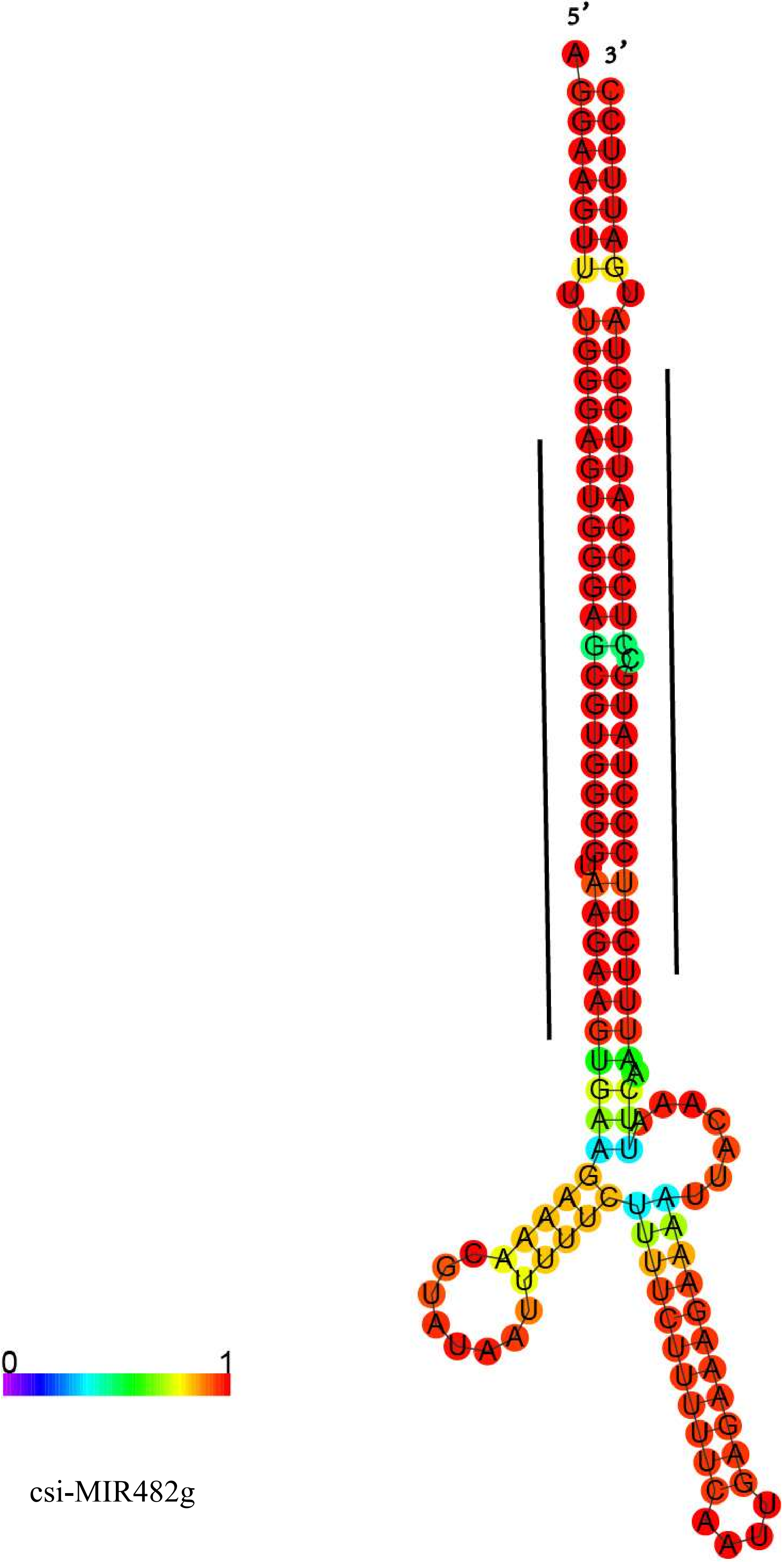

csi-MIR482g

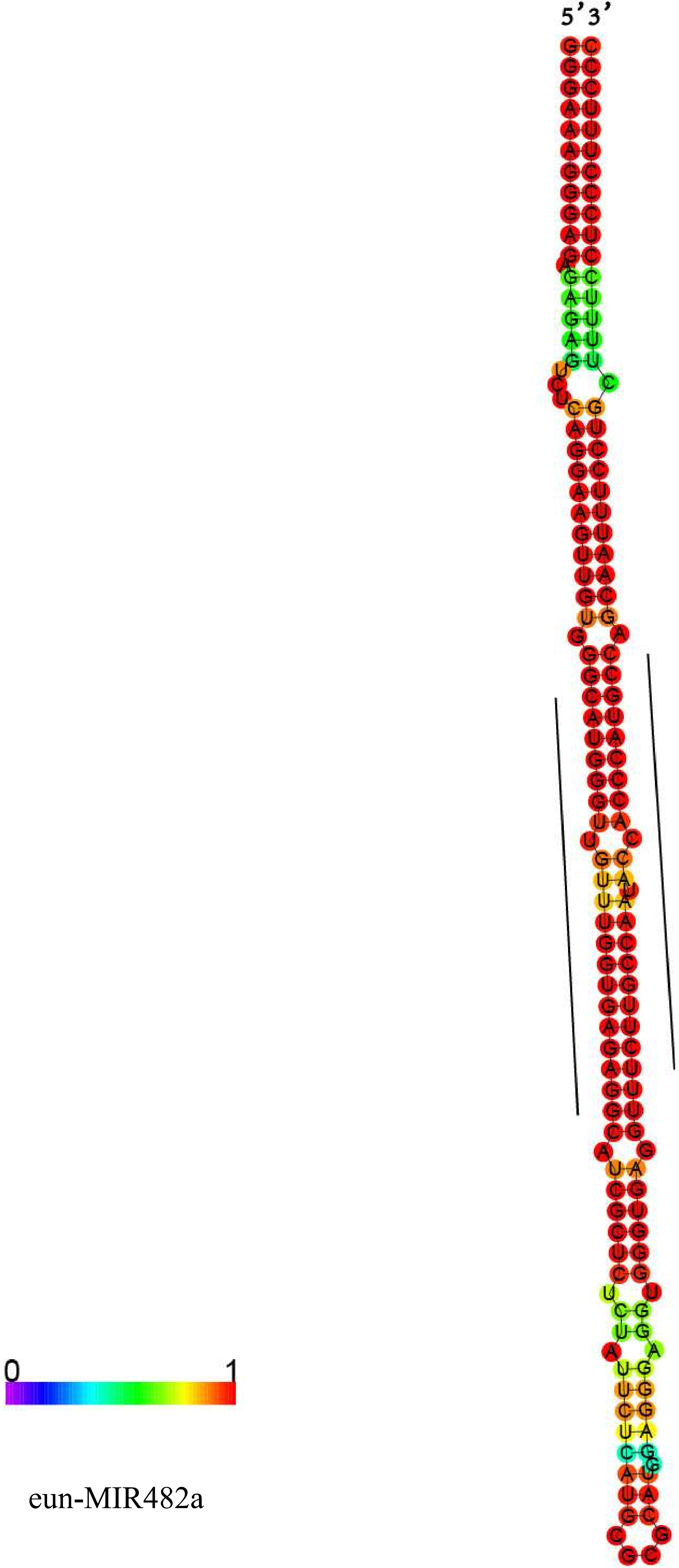

eun-MIR482a

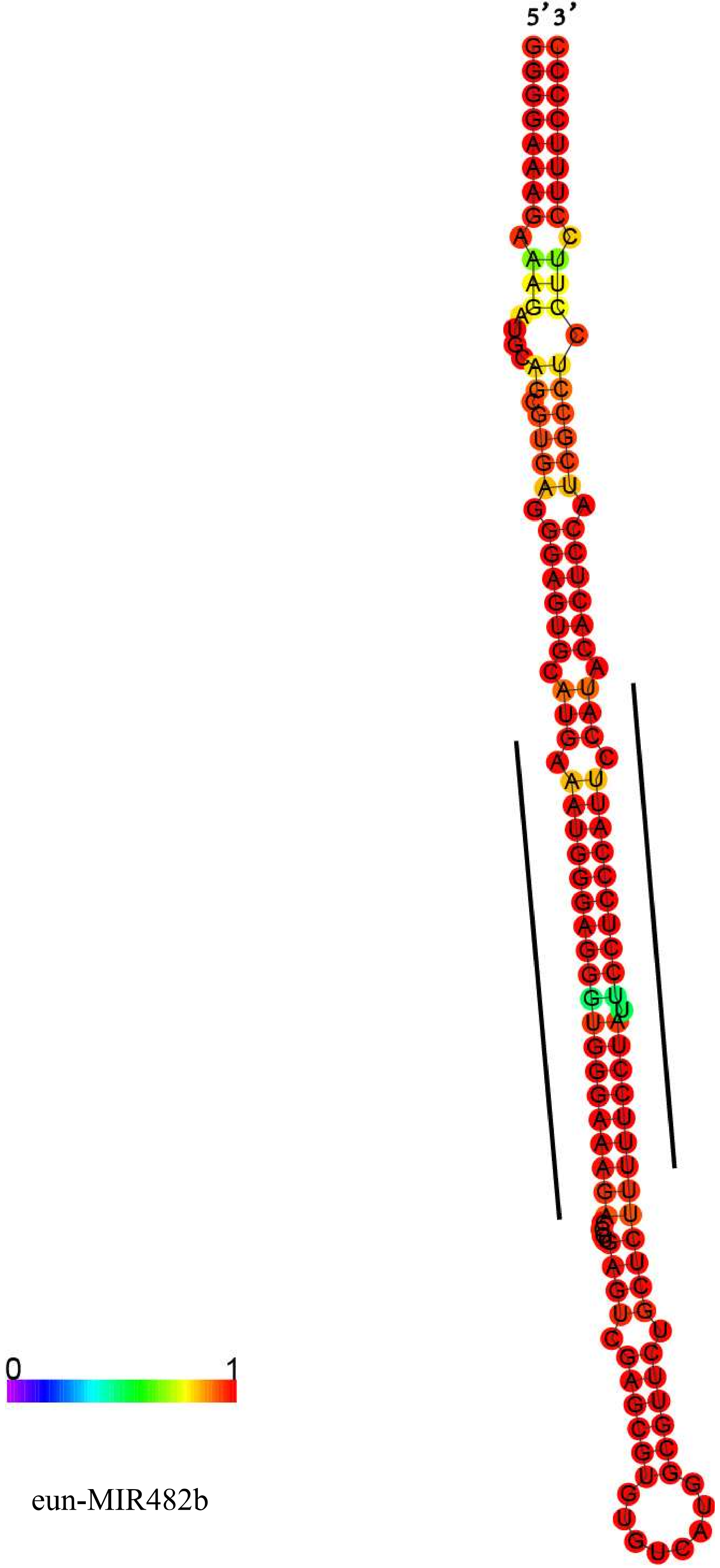

eun-MIR482b

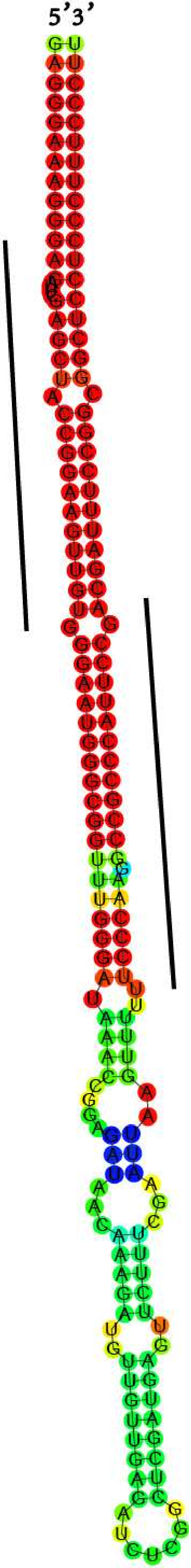

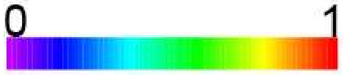

eun-MIR482c

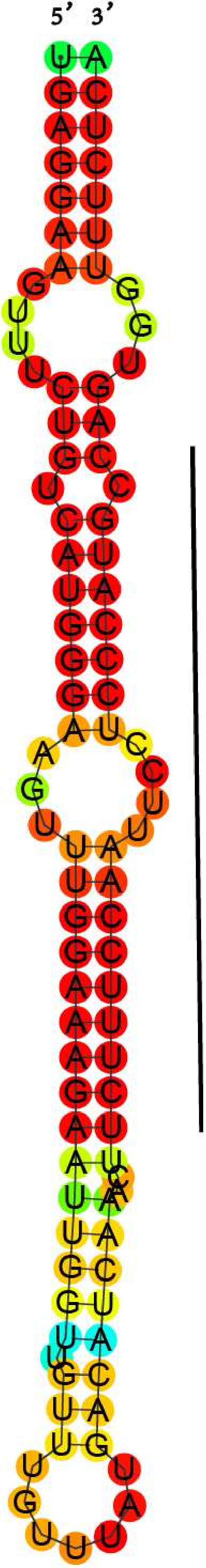

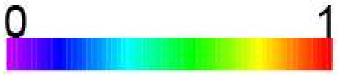

fve-MIR482a

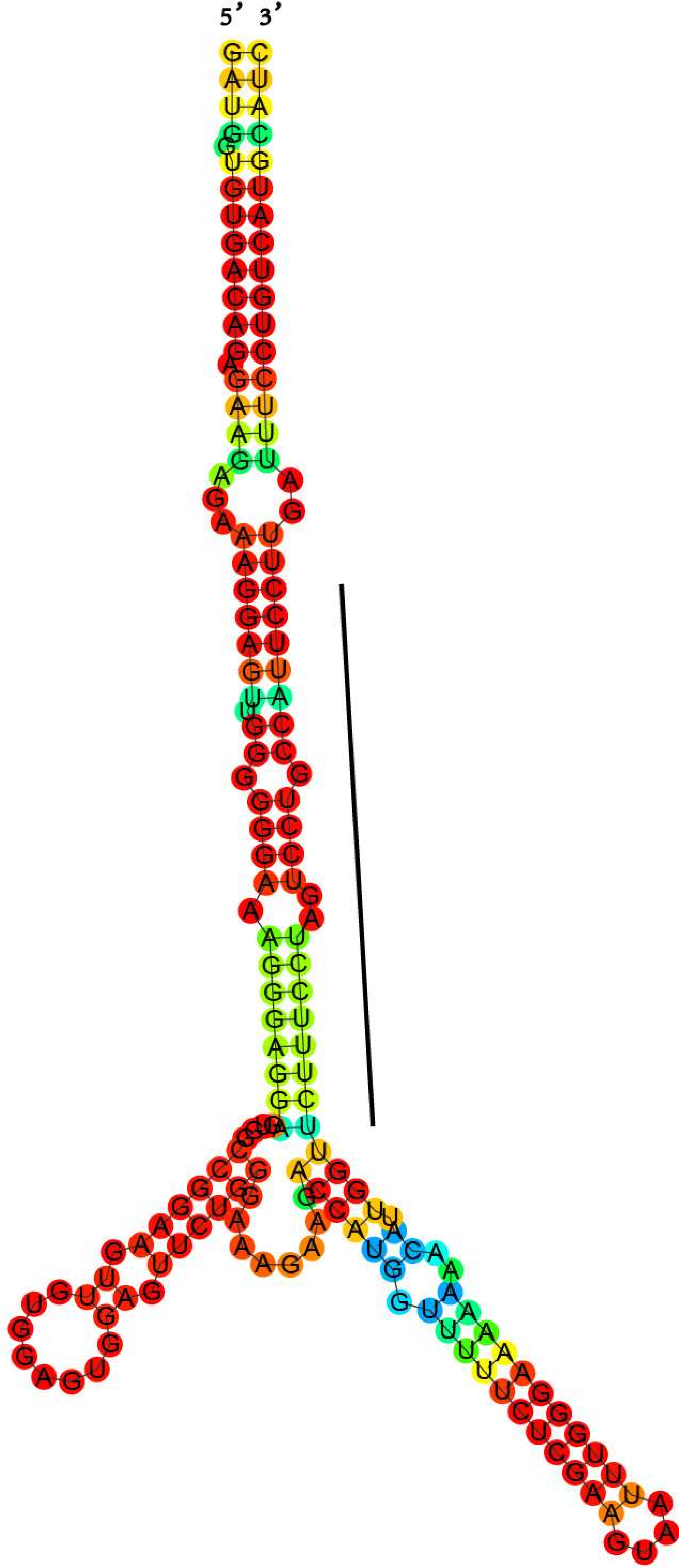

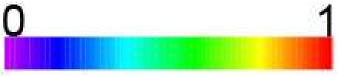

fve-MIR482b

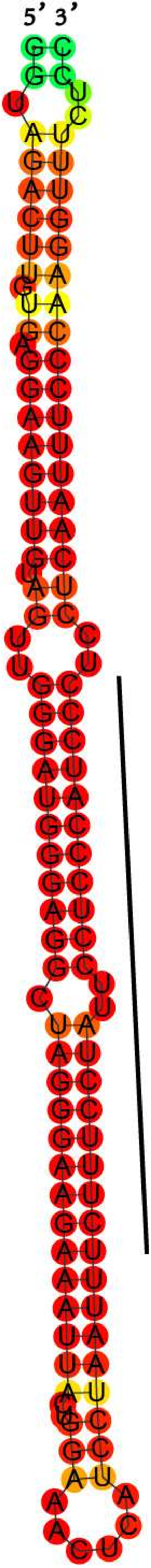

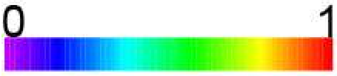

fve-MIR482c

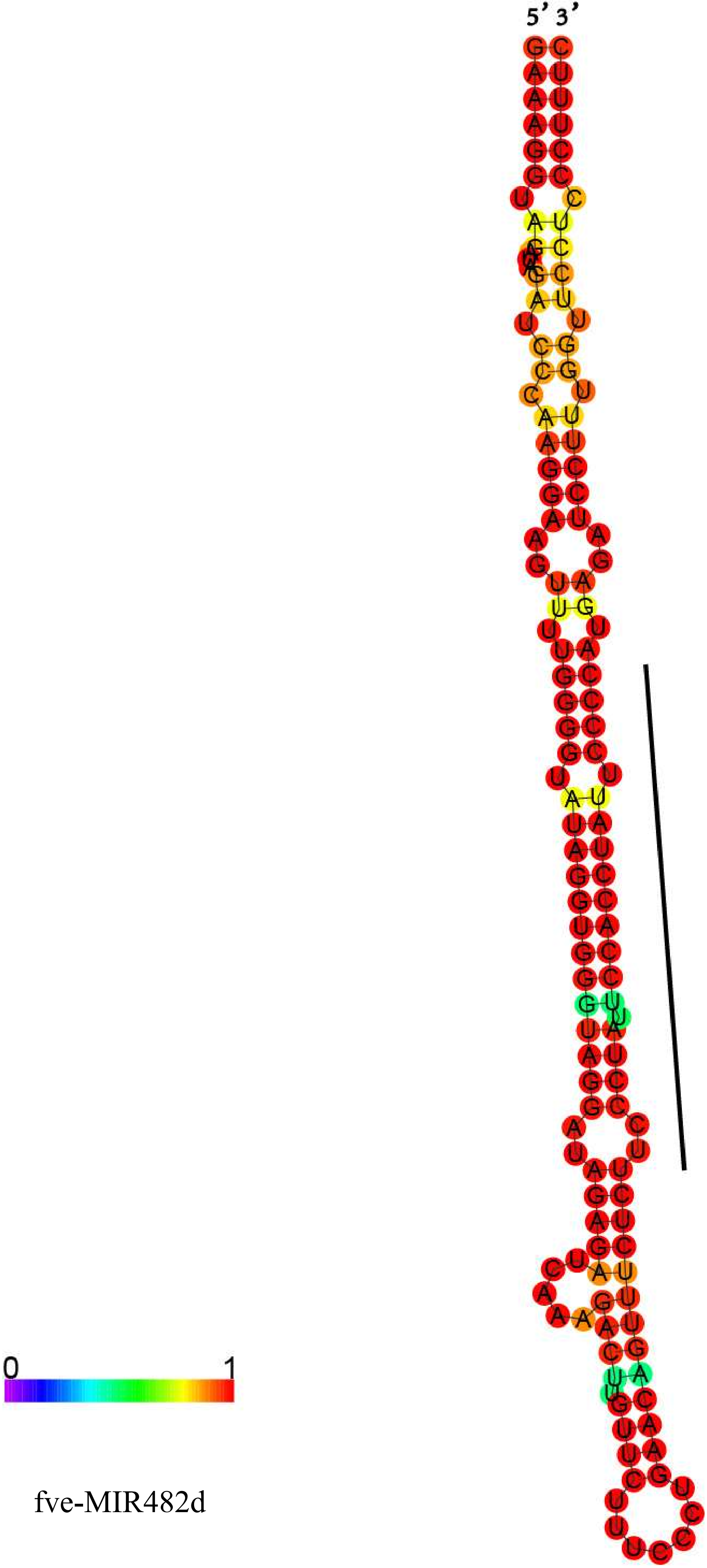

fve-MIR482d

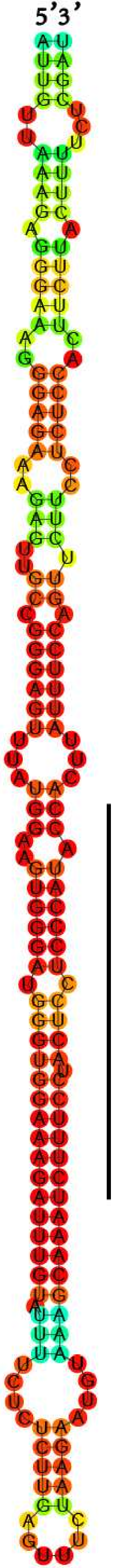

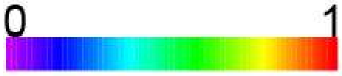

ghr-MIR482a

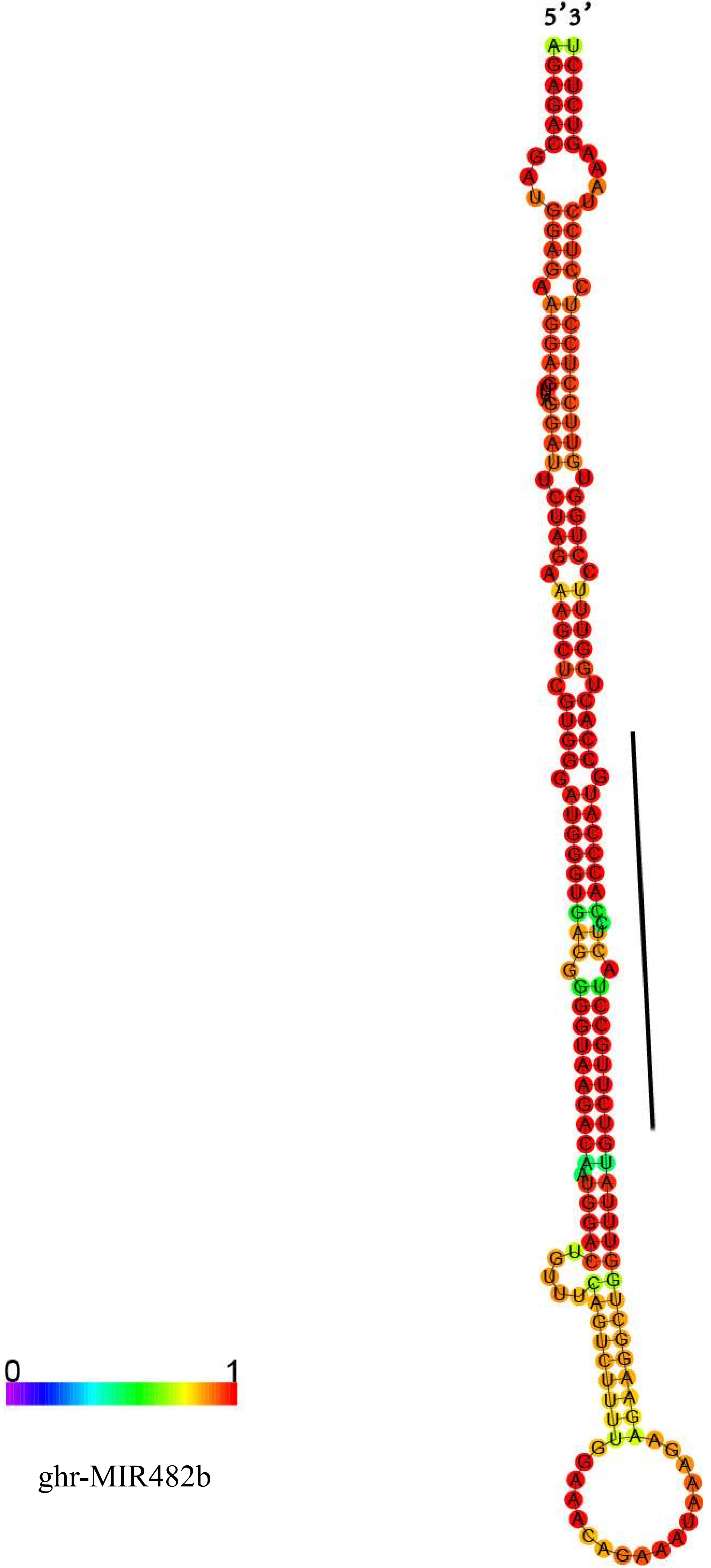

ghr-MIR482b

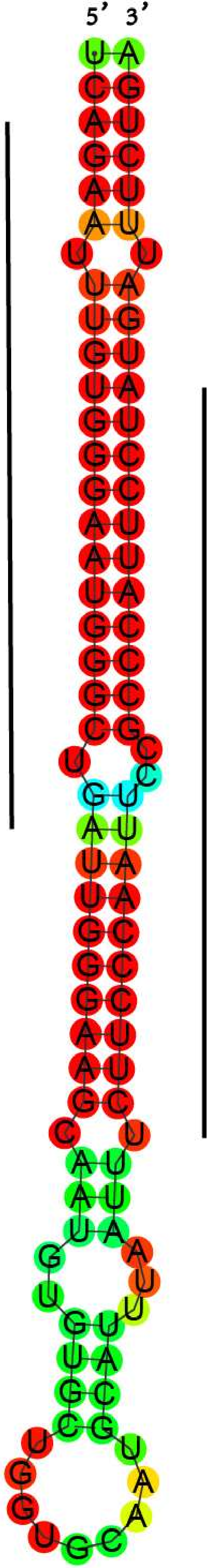

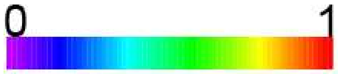

gma-MIR482a

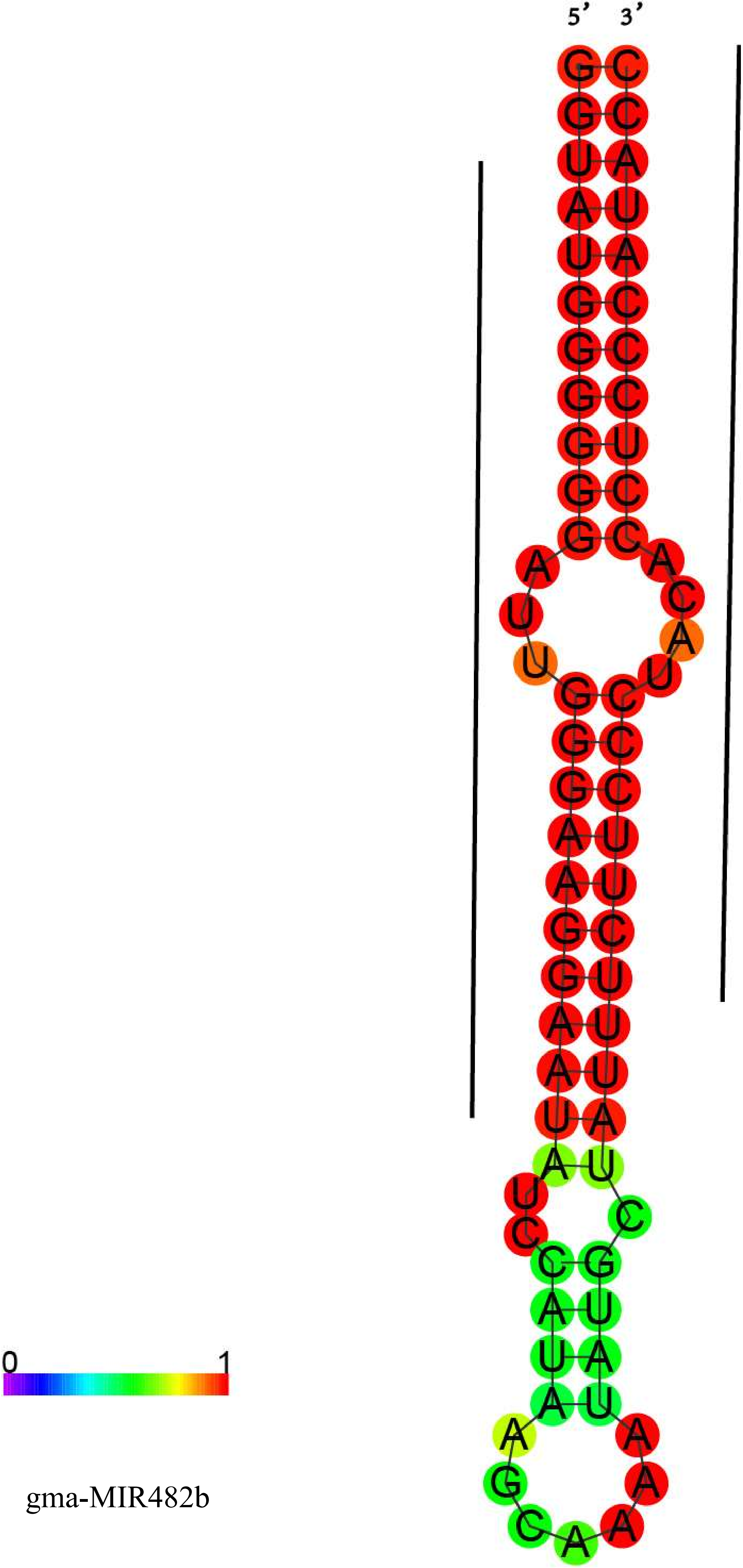

gma-MIR482b

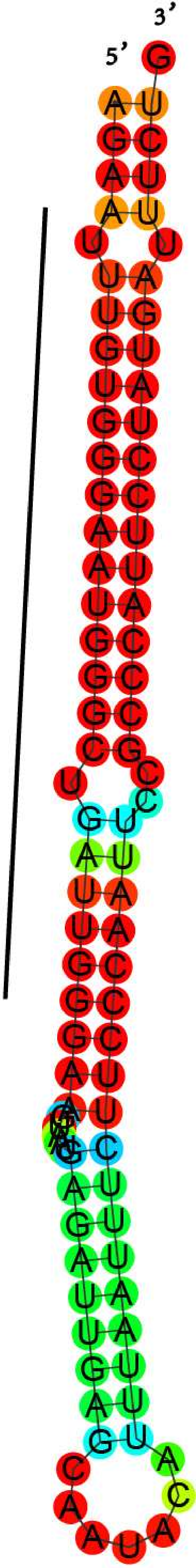

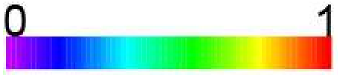

gma-MIR482c

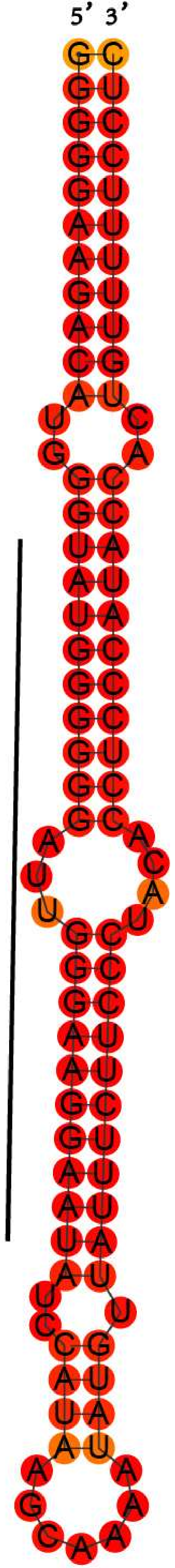

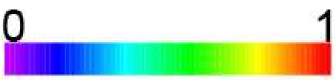

gma-MIR482d

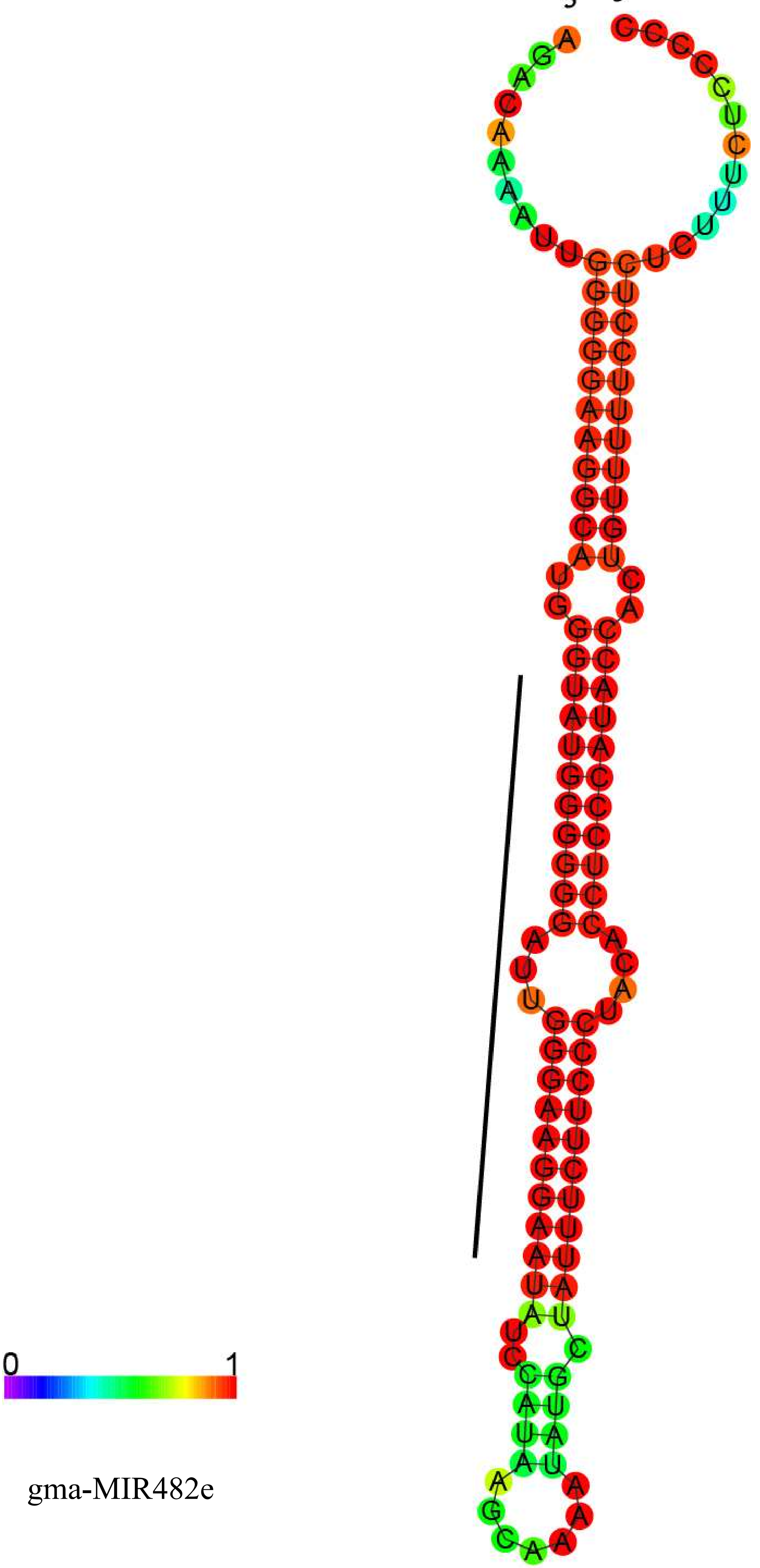

gma-MIR482e

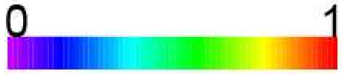

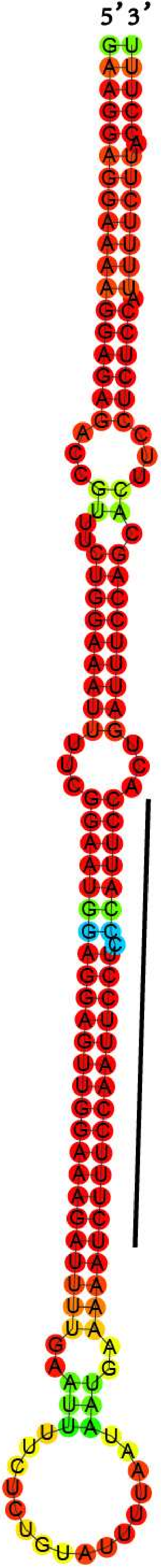

gra-MIR482

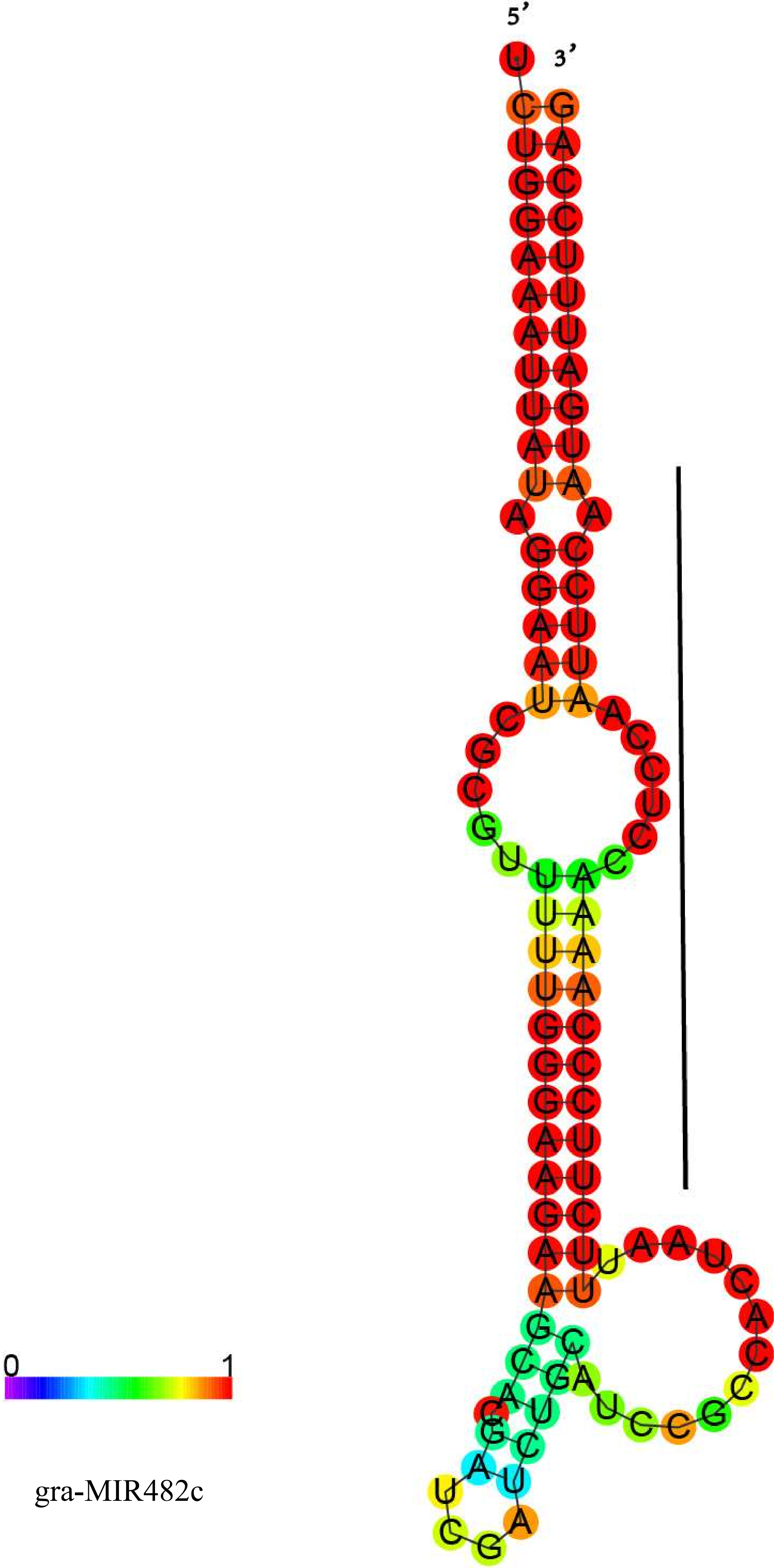

gra-MIR482c

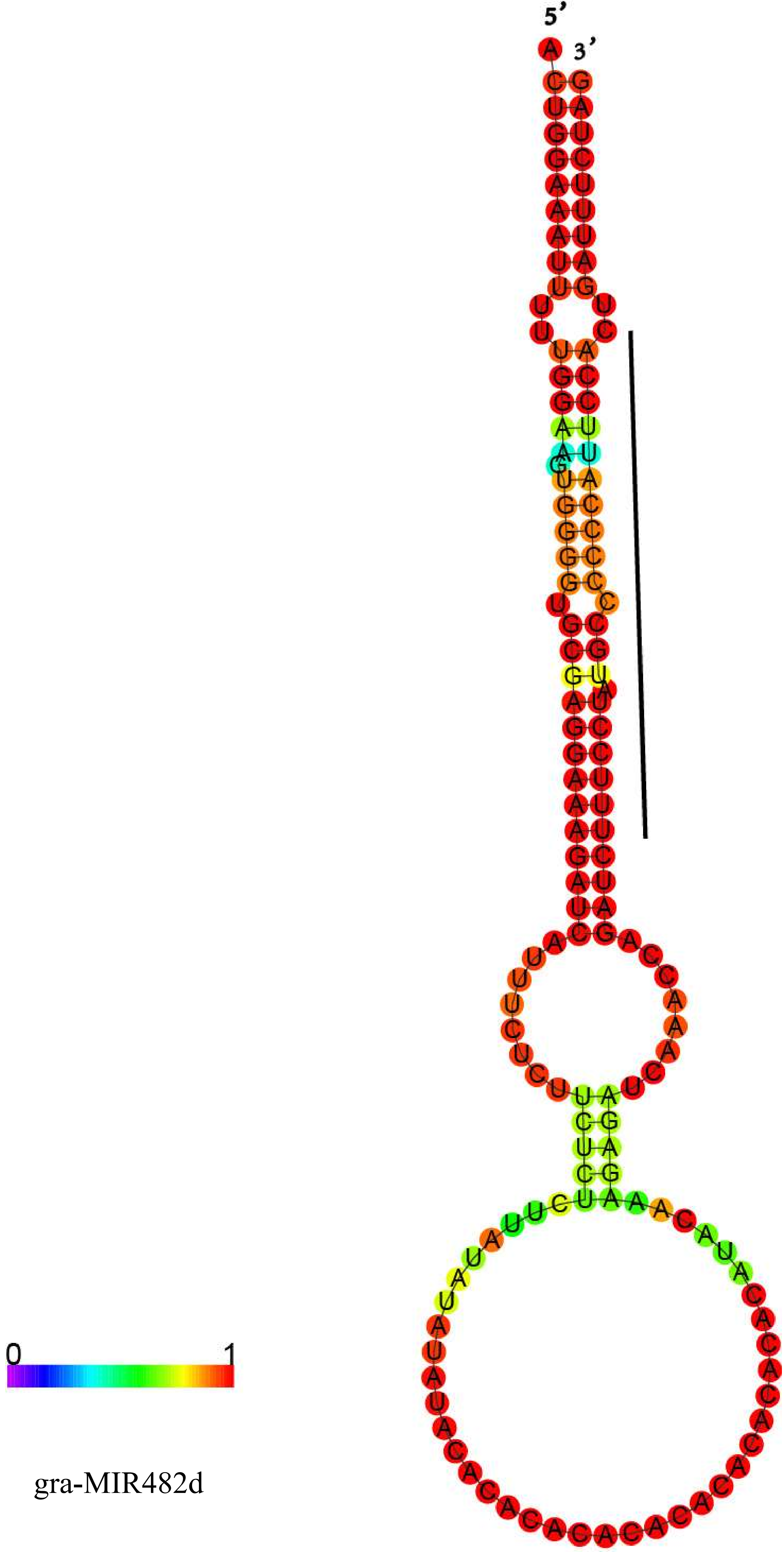

gra-MIR482d

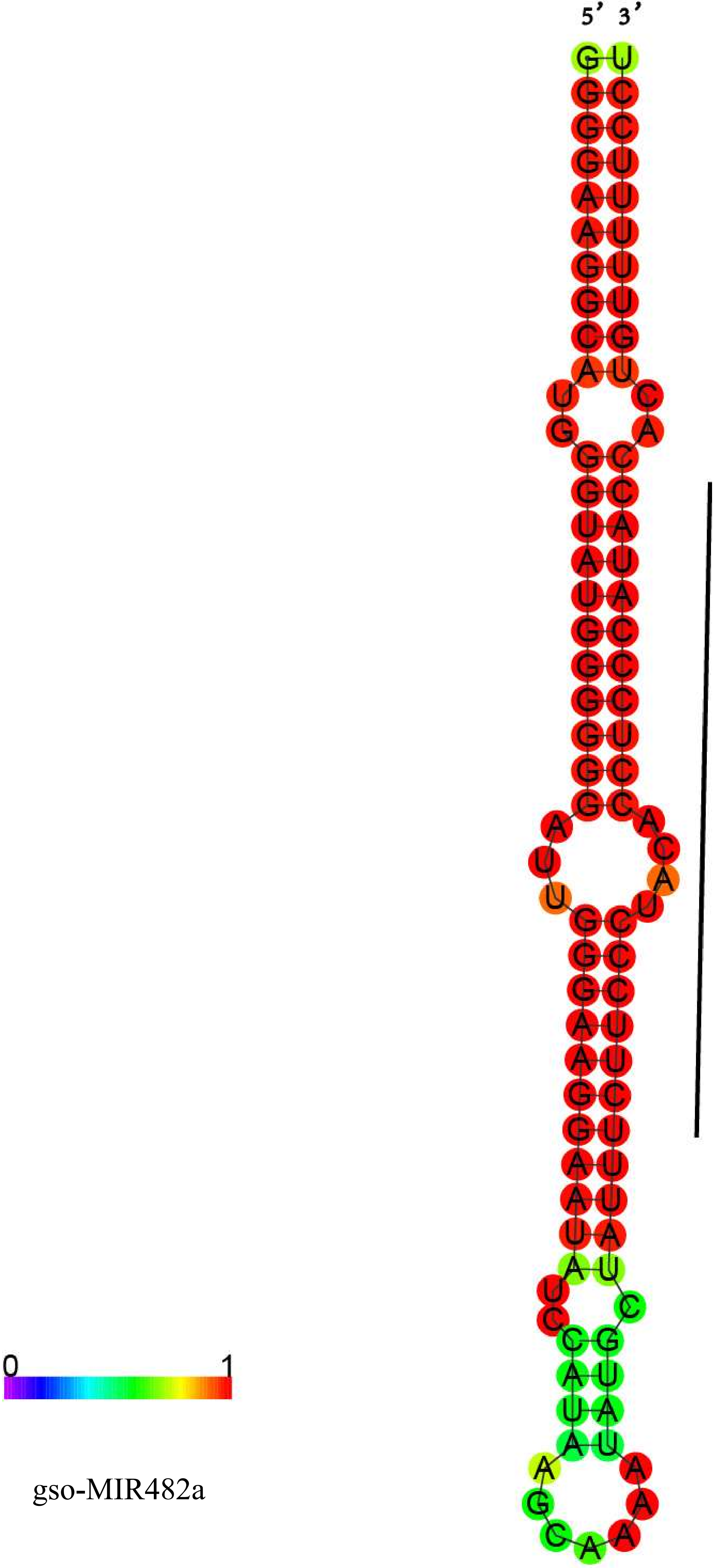

gso-MIR482a

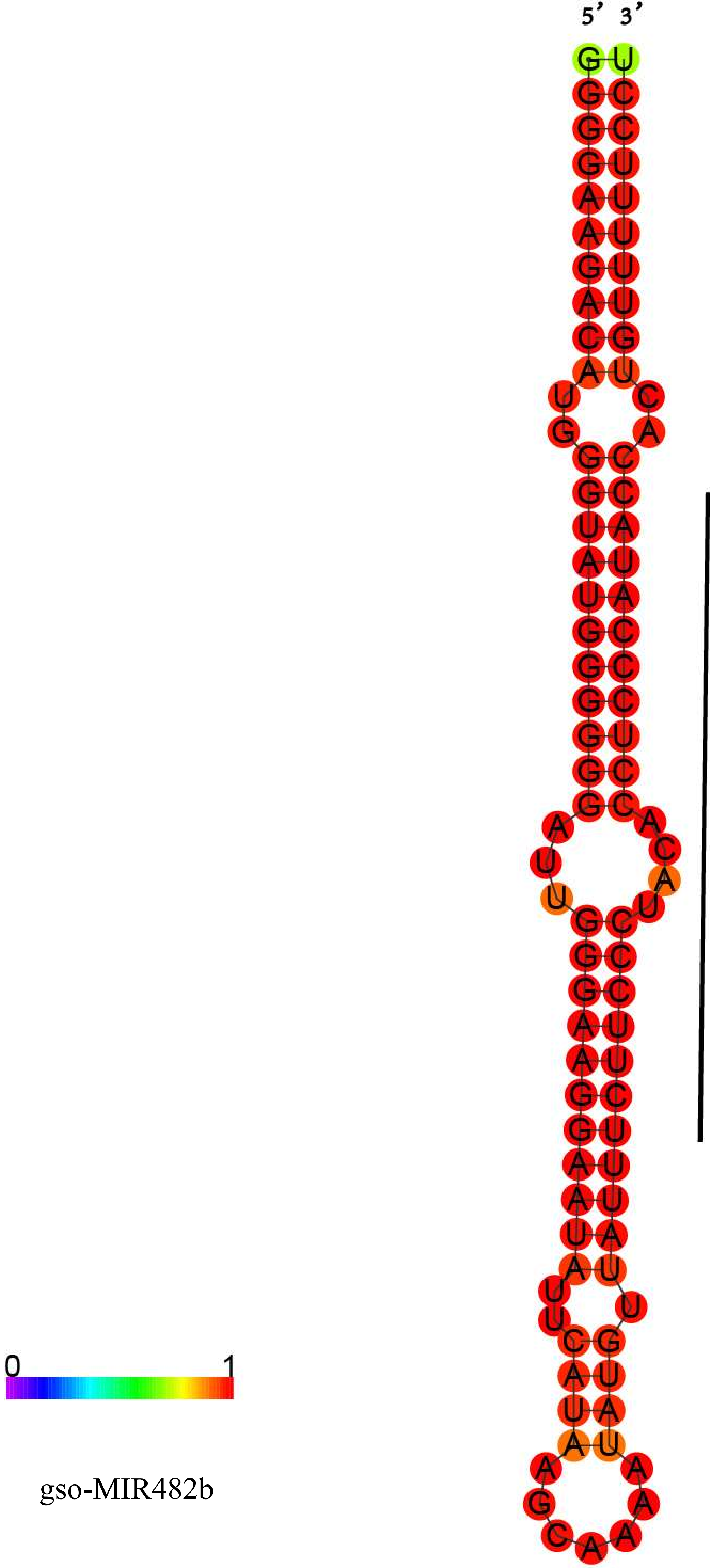

gso-MIR482b

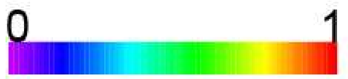

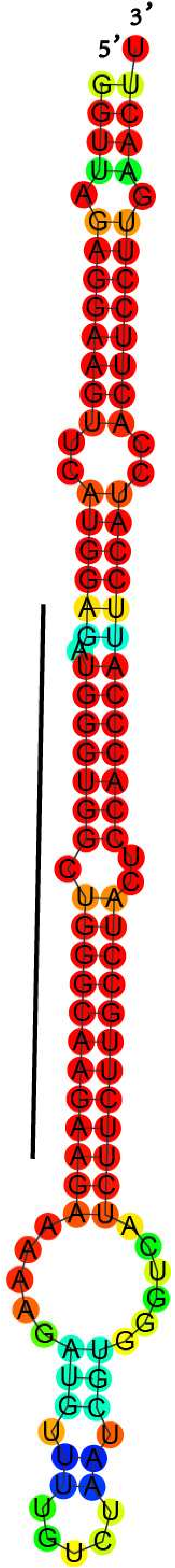

hbr-MIR482a

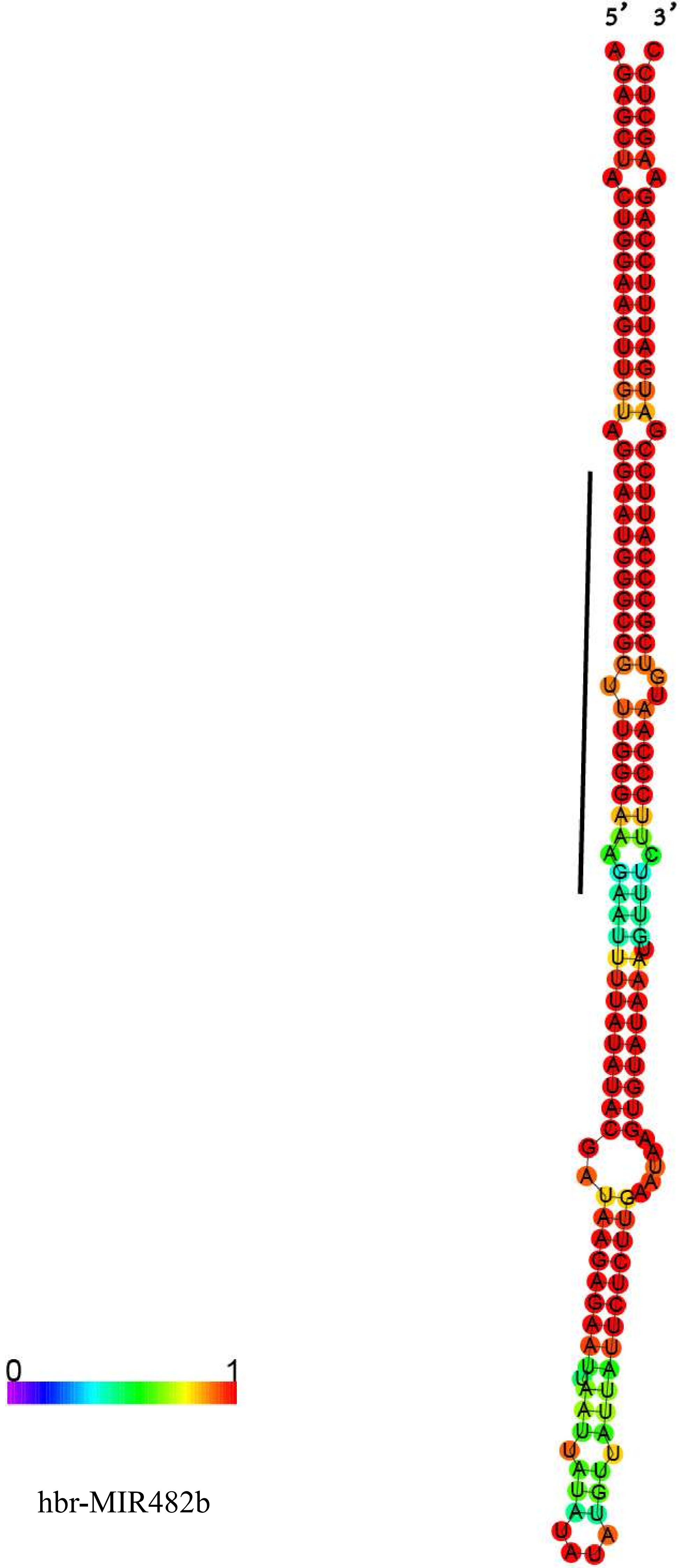

hbr-MIR482b

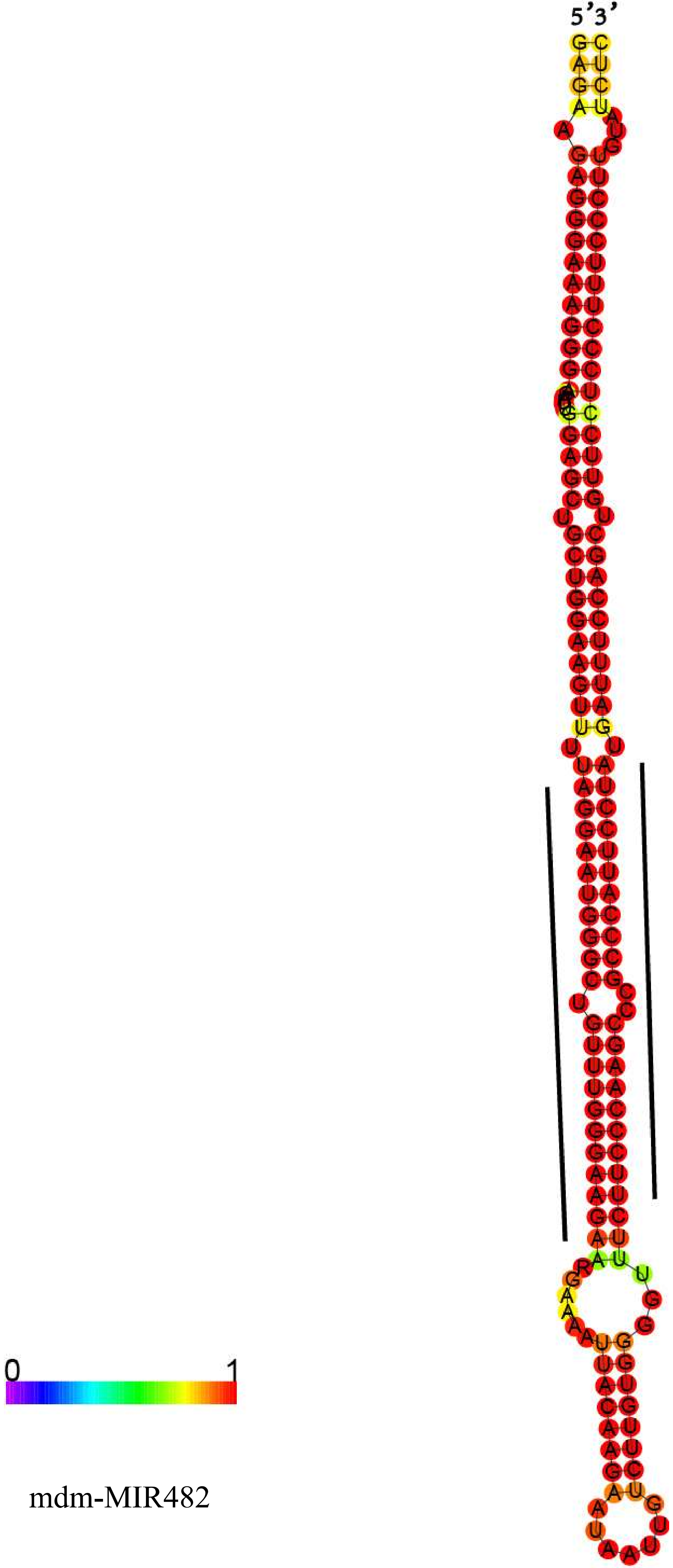

mdm-MIR482

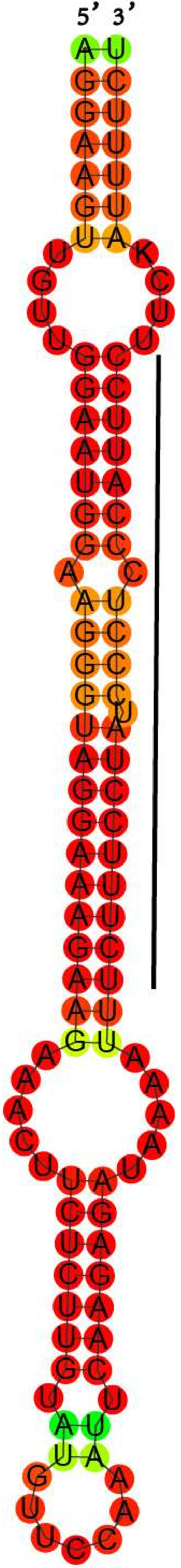

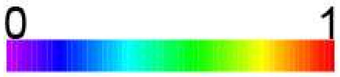

mdm-MIR482b

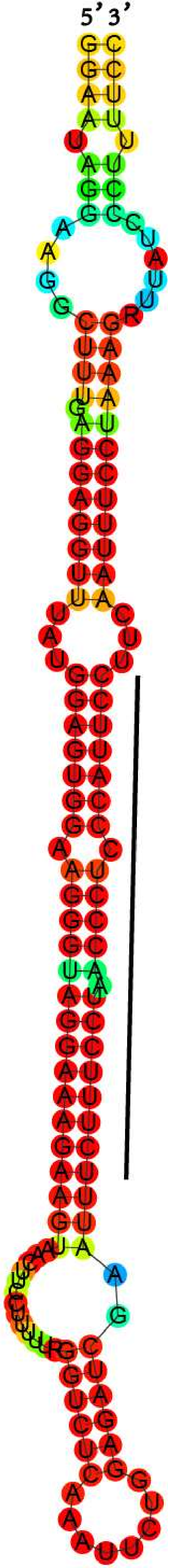

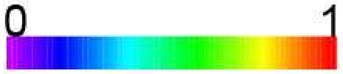

mdm-MIR482c

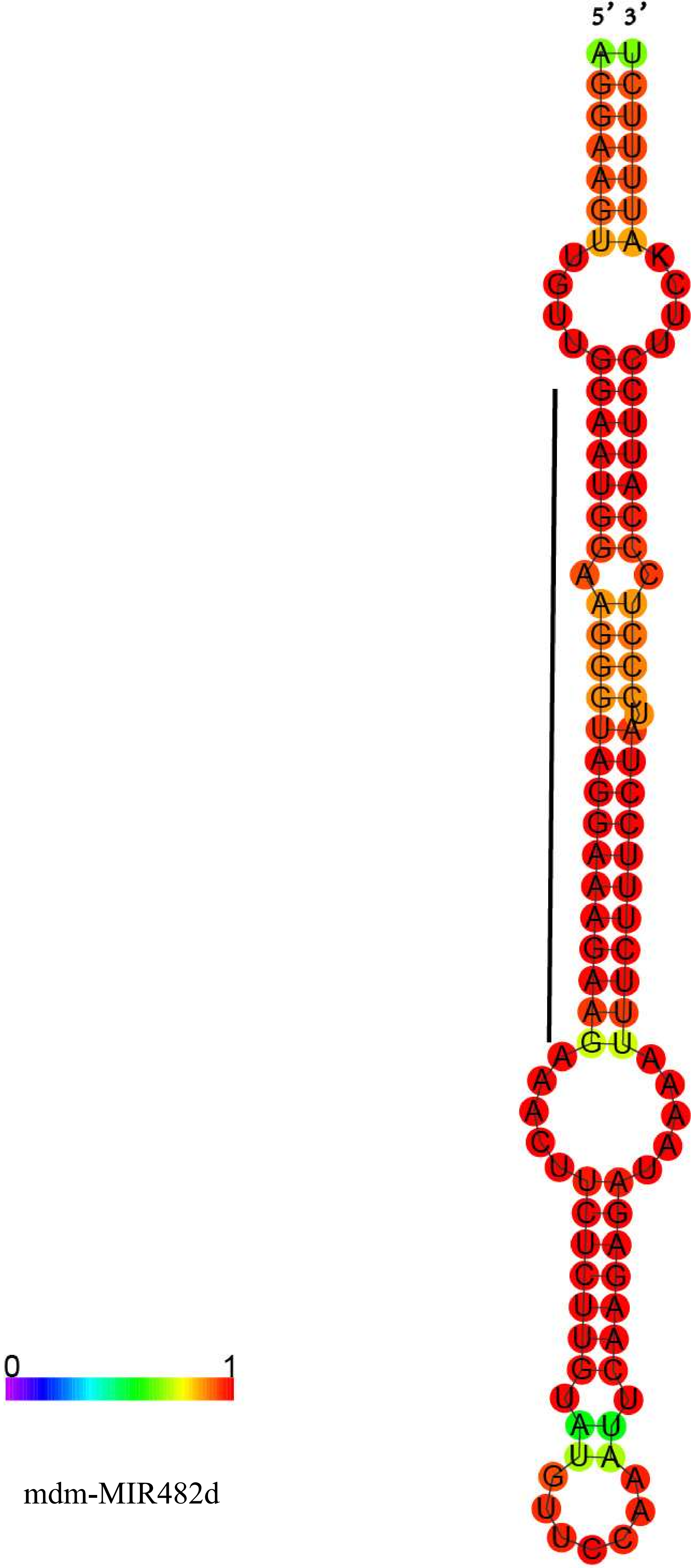

mdm-MIR482d

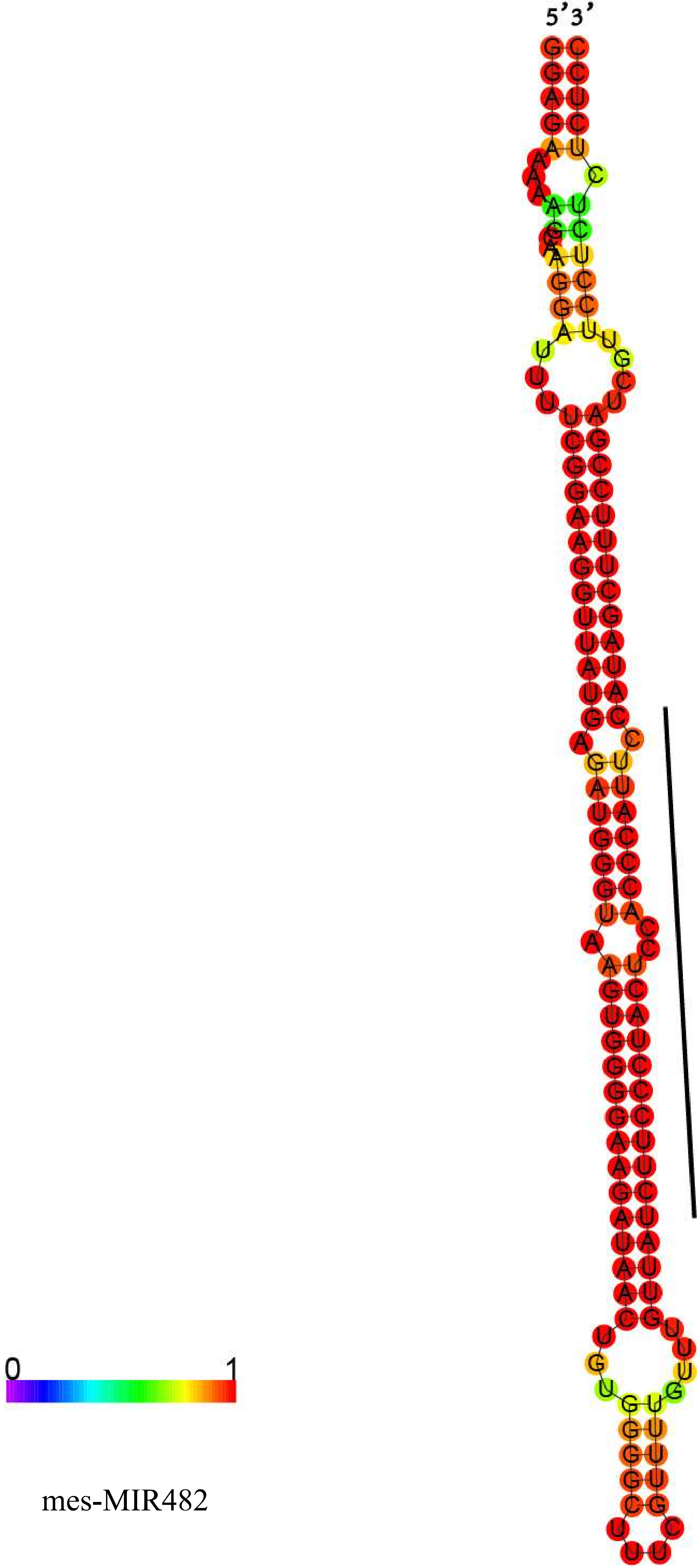

mes-MIR482

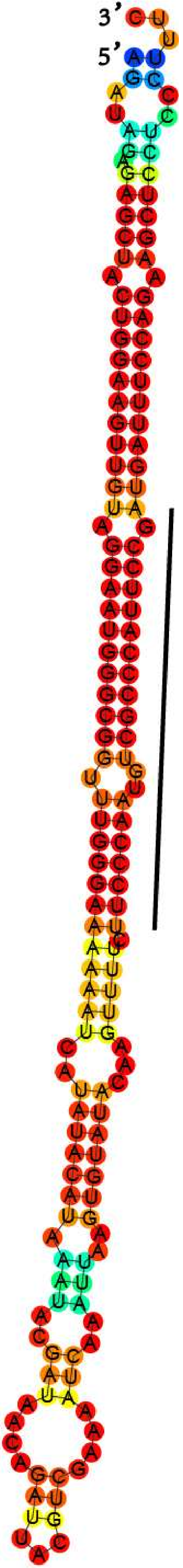

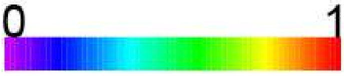

mes-MIR482b

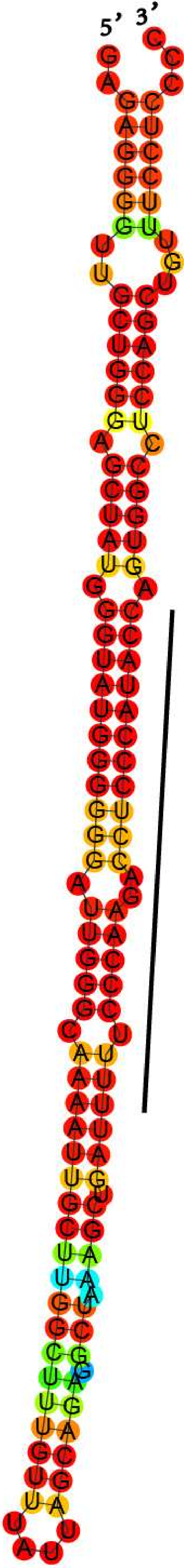

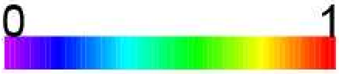

mes-MIR482c

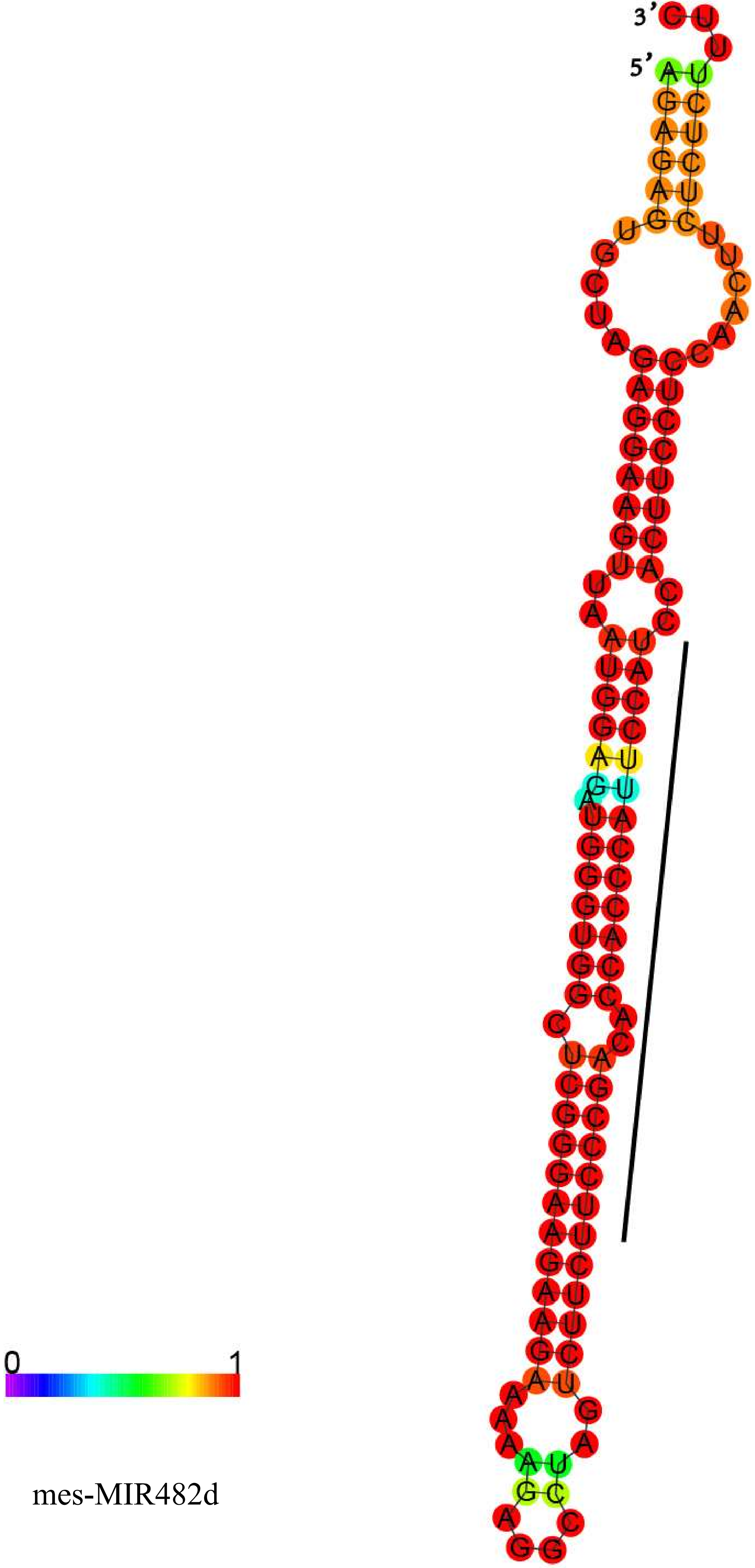

mes-MIR482d

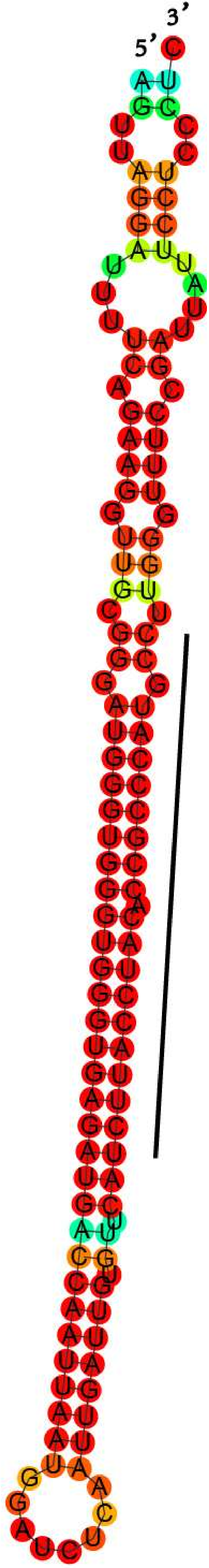

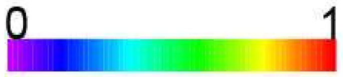

mes-MIR482e

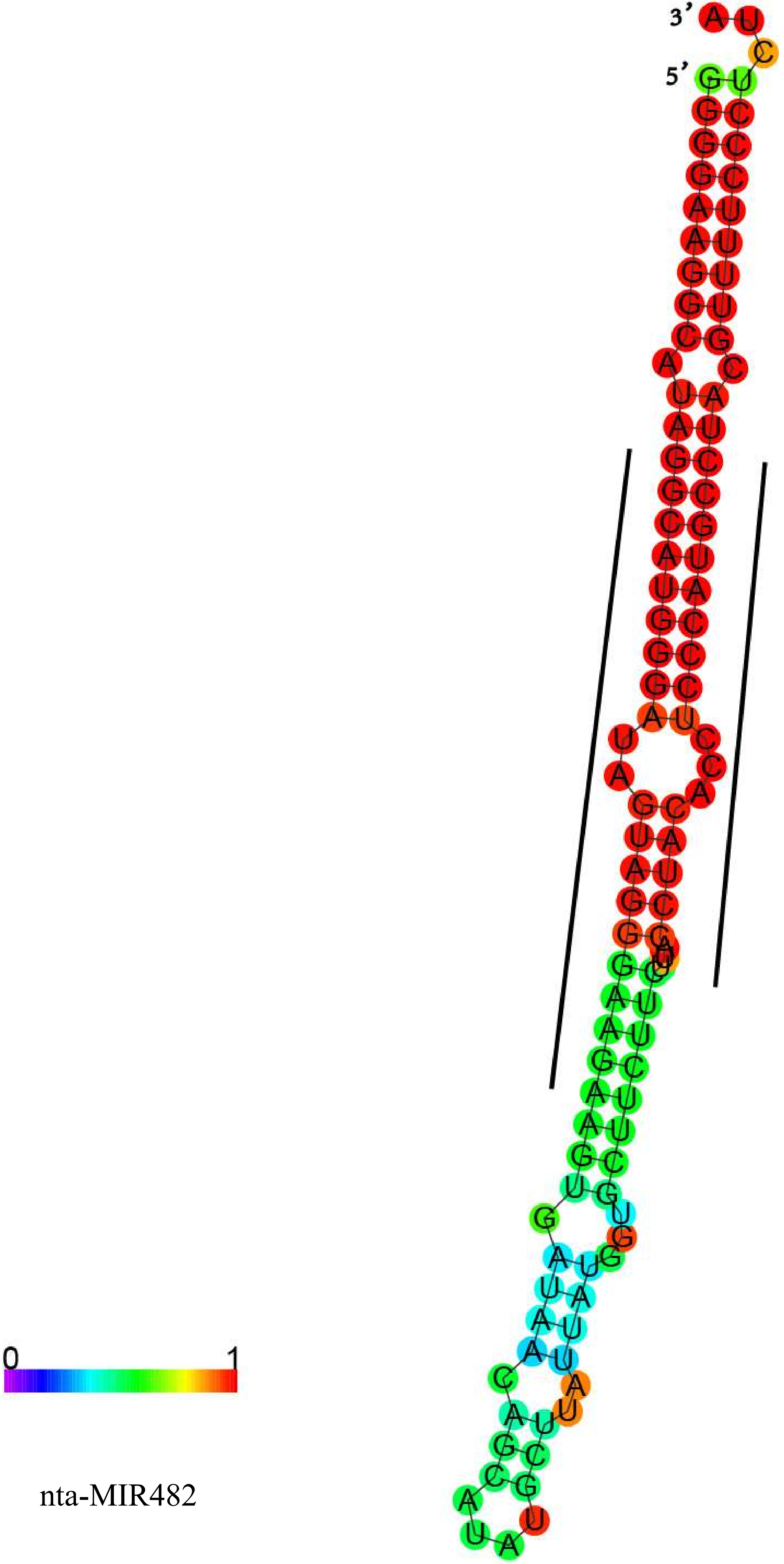

nta-MIR482

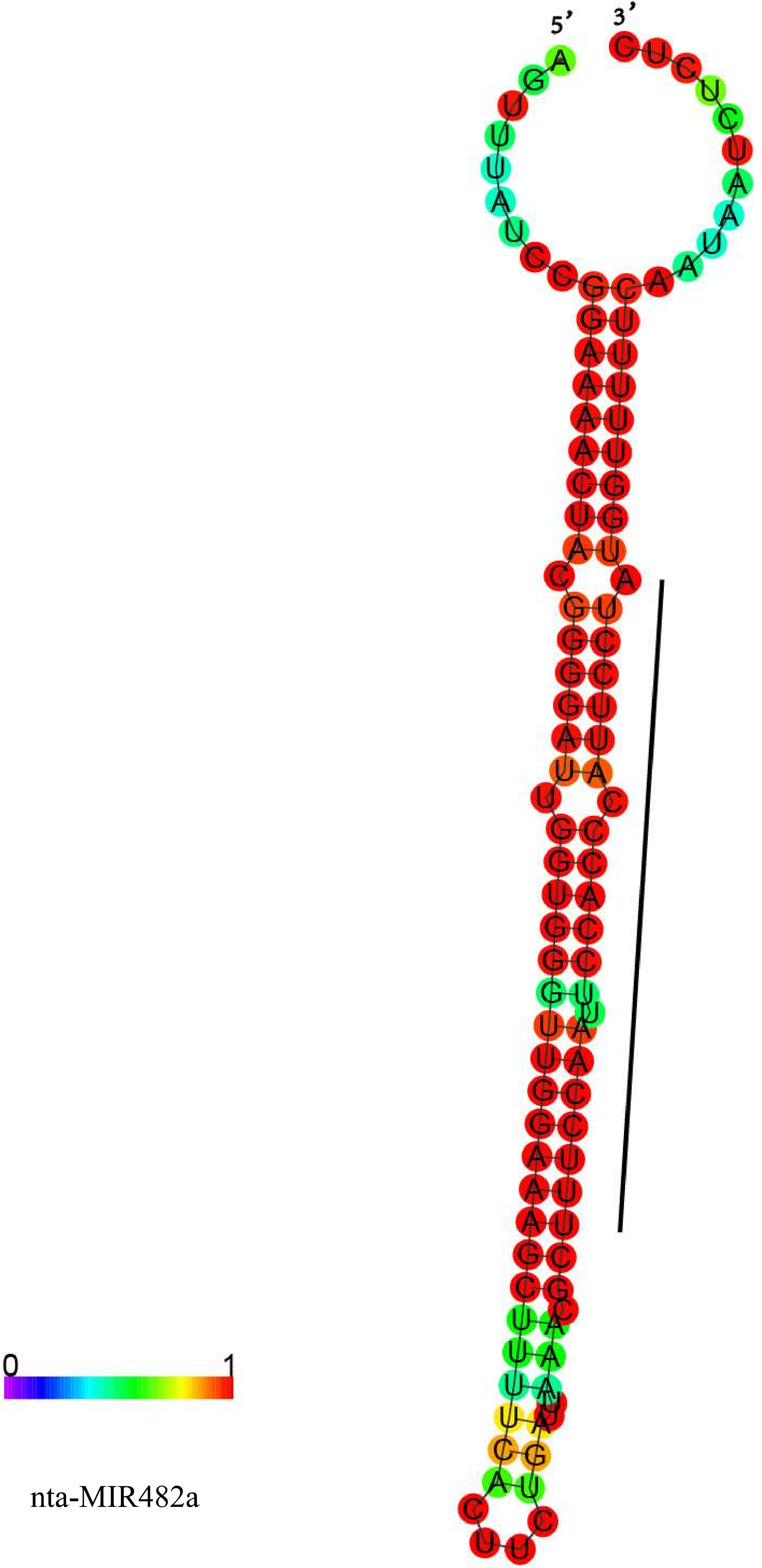

nta-MIR482a

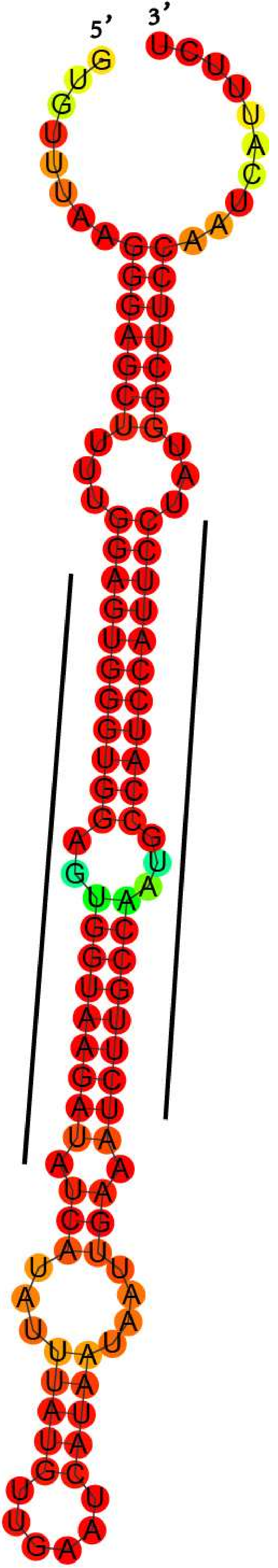

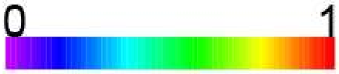

nta-MIR482b

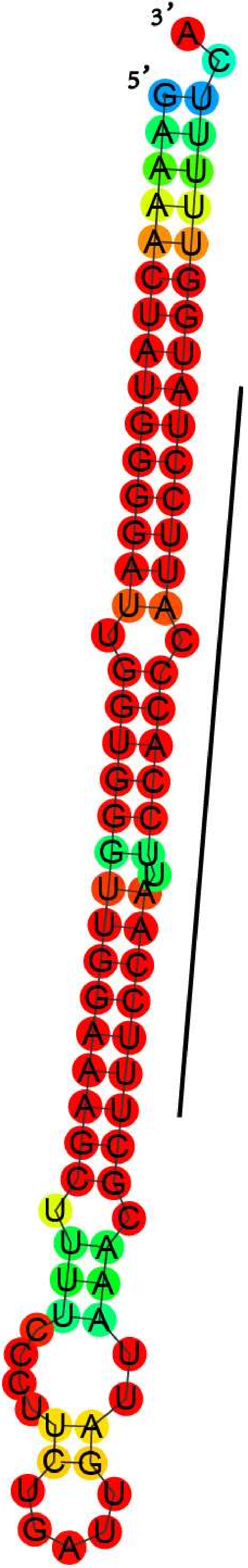

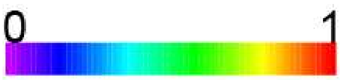

nta-MIR482c

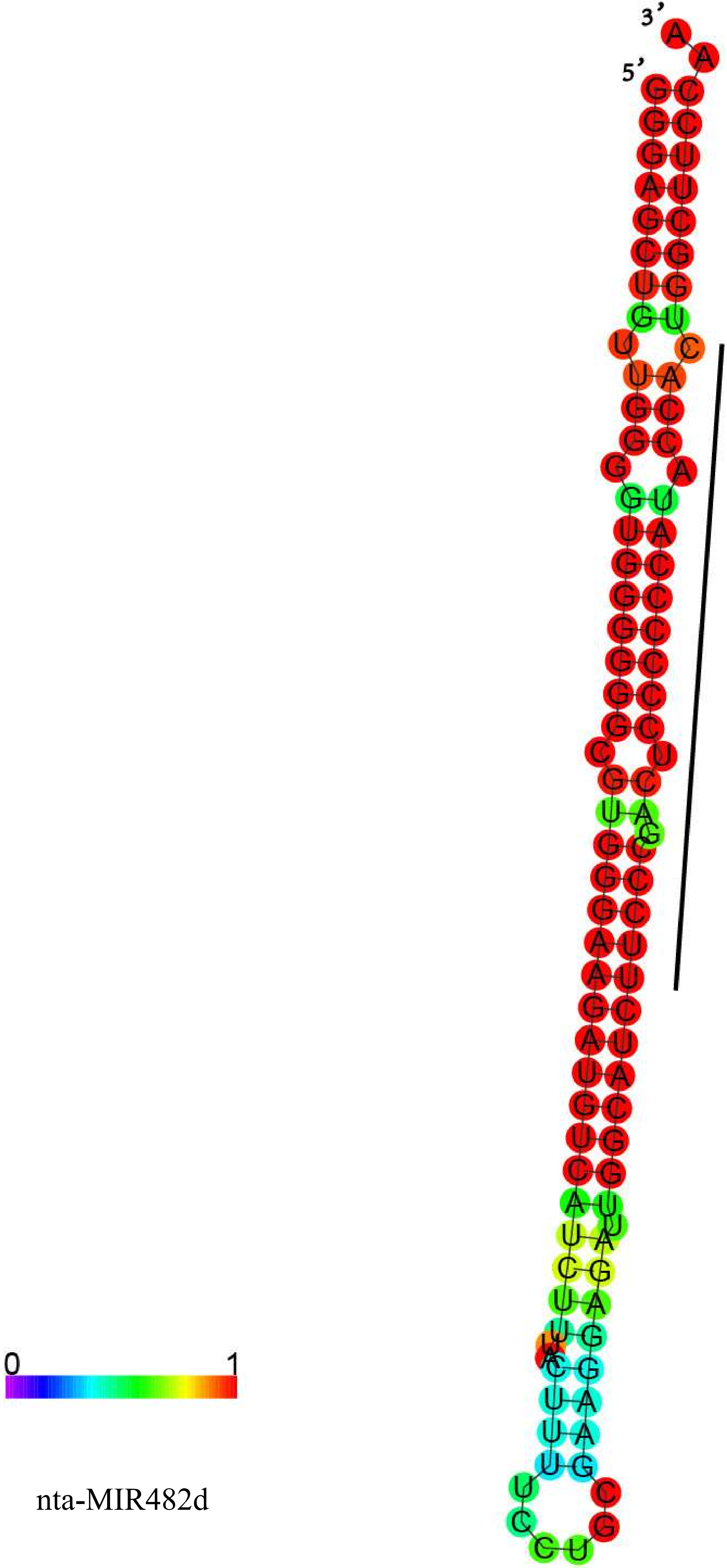

nta-MIR482d

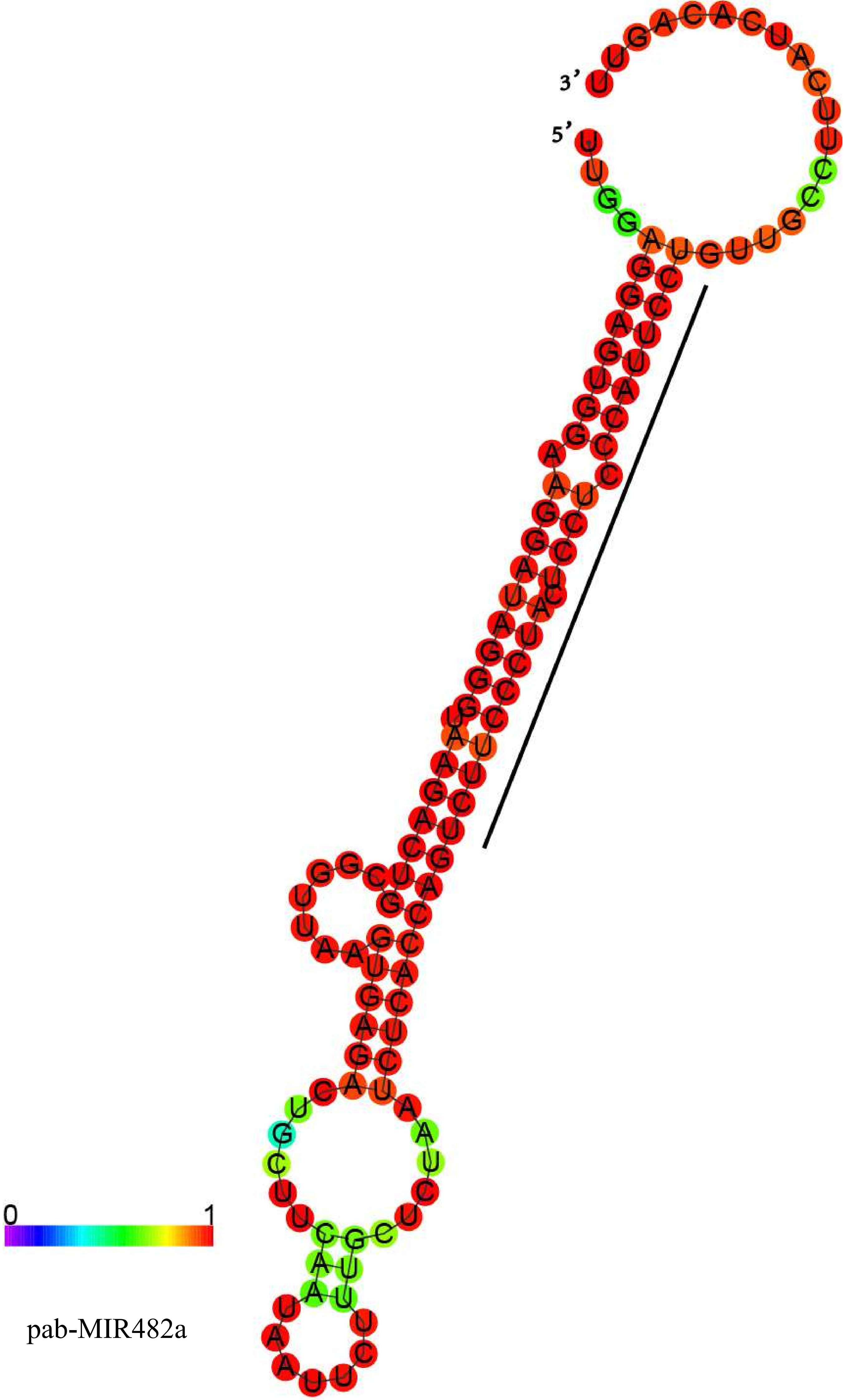

pab-MIR482a

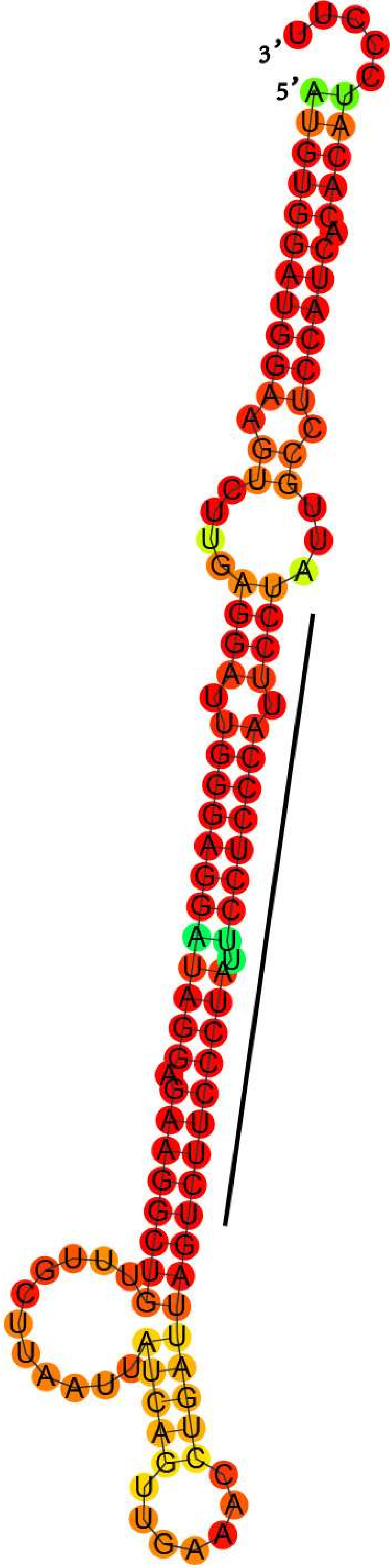

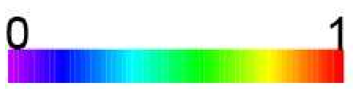

pab-MIR482b

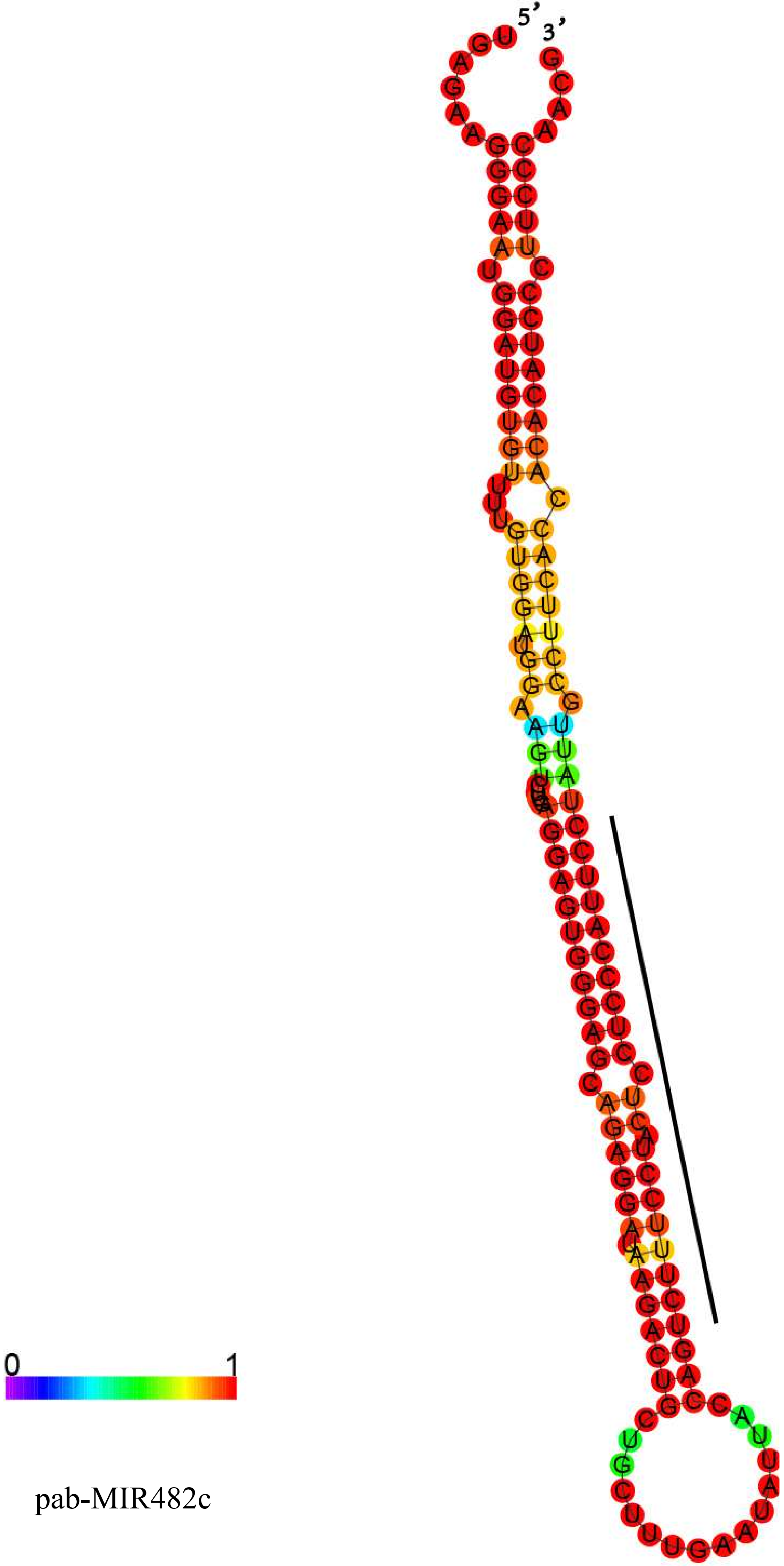

pab-MIR482c

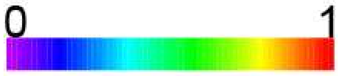

pab-MIR482d

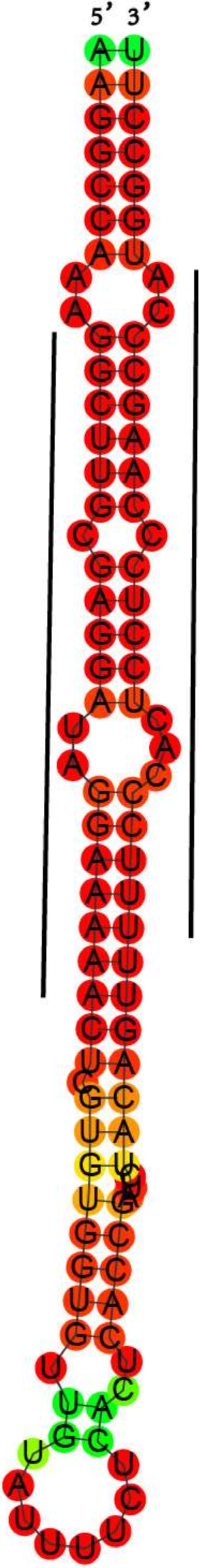

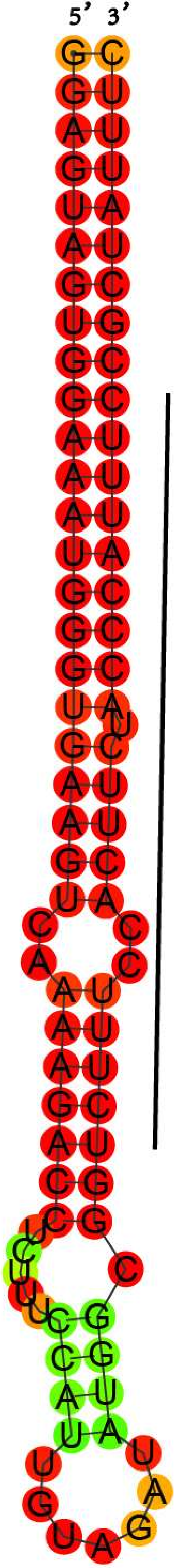

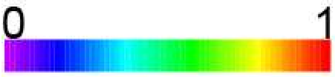

pab-MIR482e

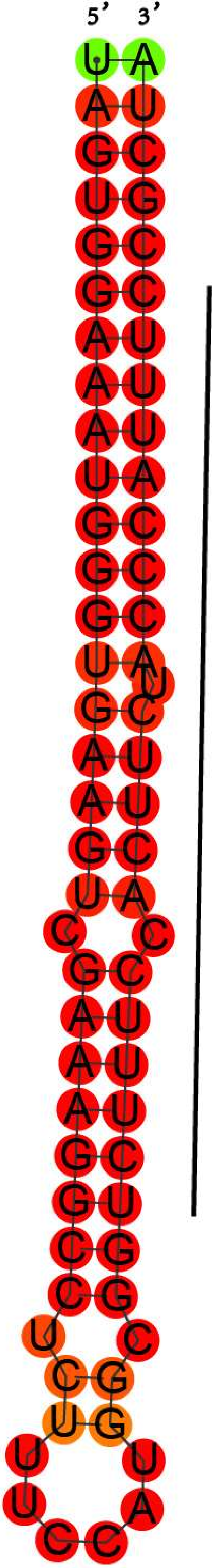

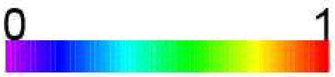

pab-MIR482f

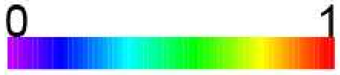

pab-MIR482g

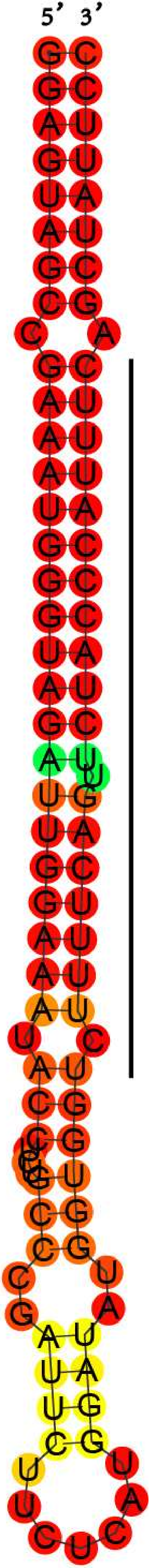

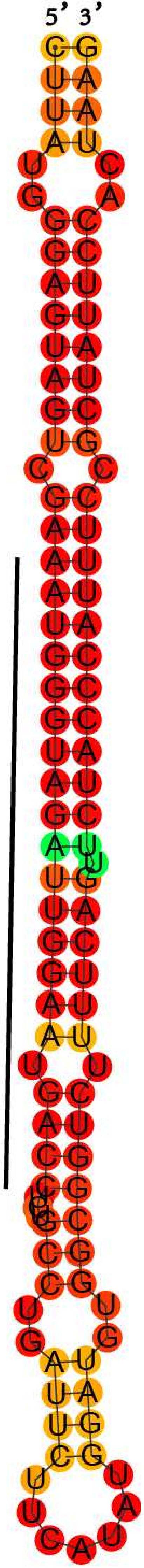

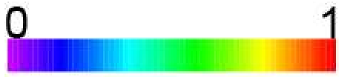

pab-MIR482h

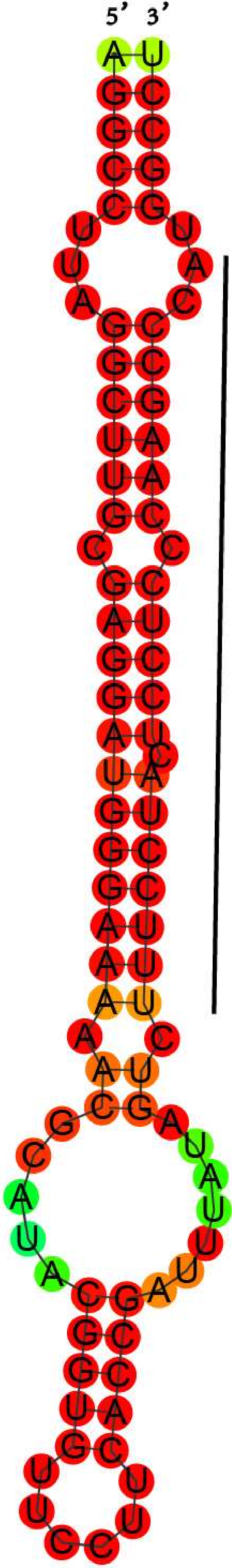

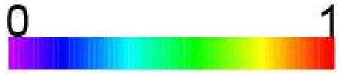

pab-MIR482i

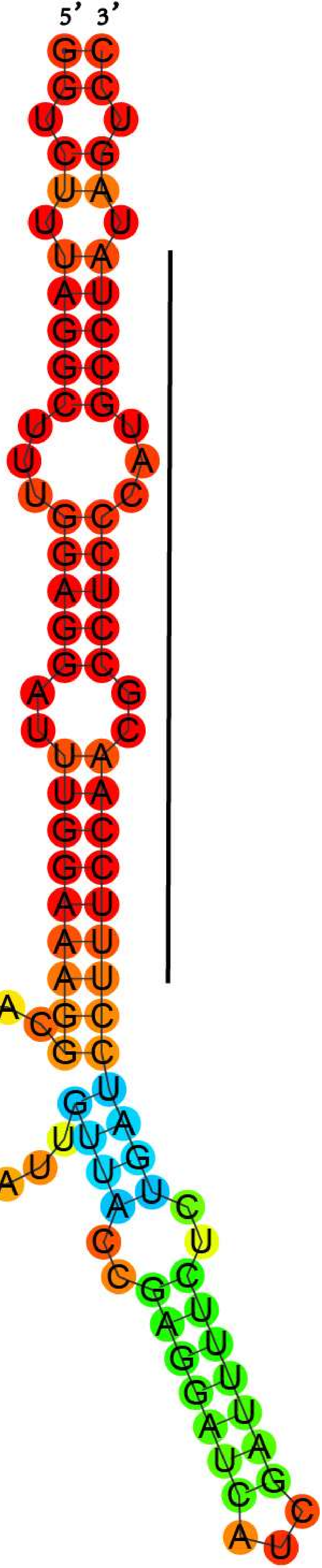

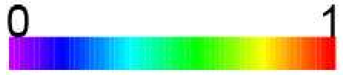

pab-MIR482j

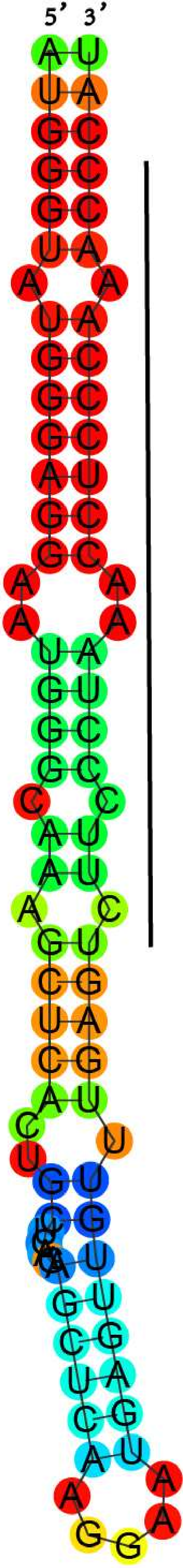

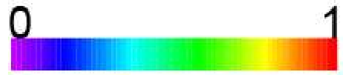

pab-MIR482k

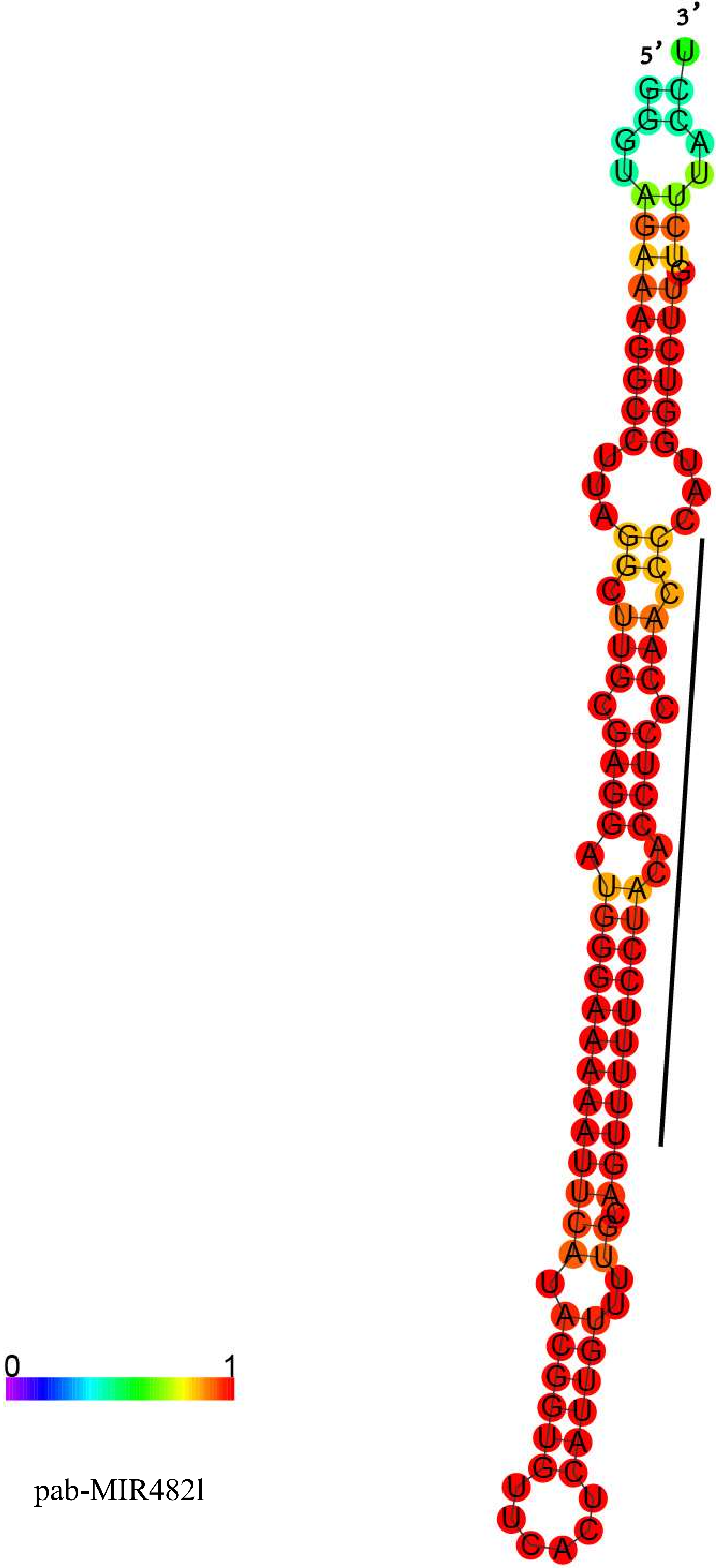

pab-MIR4821

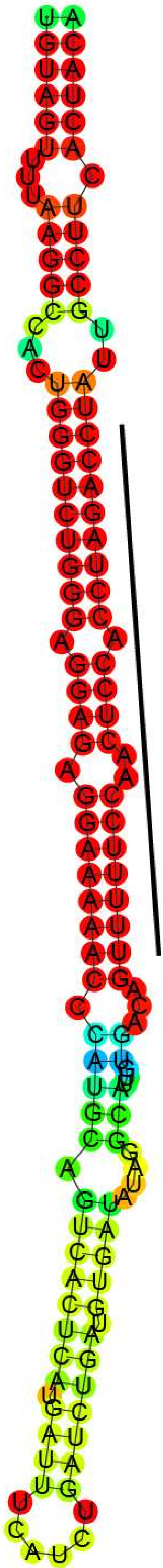

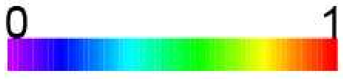

pab-MIR482m

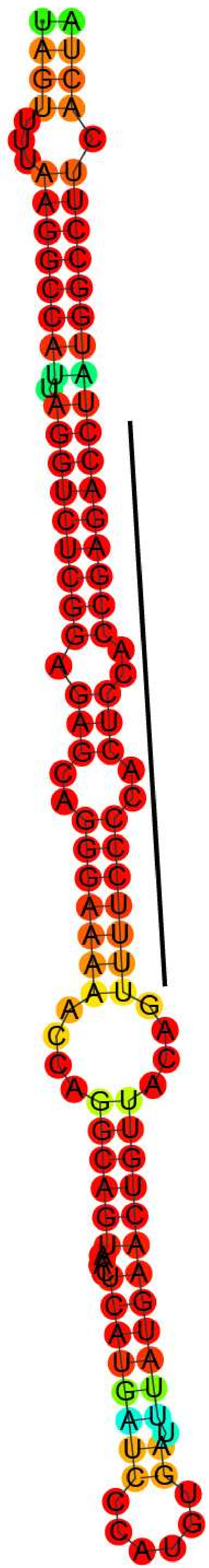

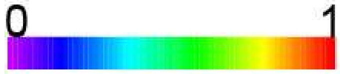

pab-MIR482n

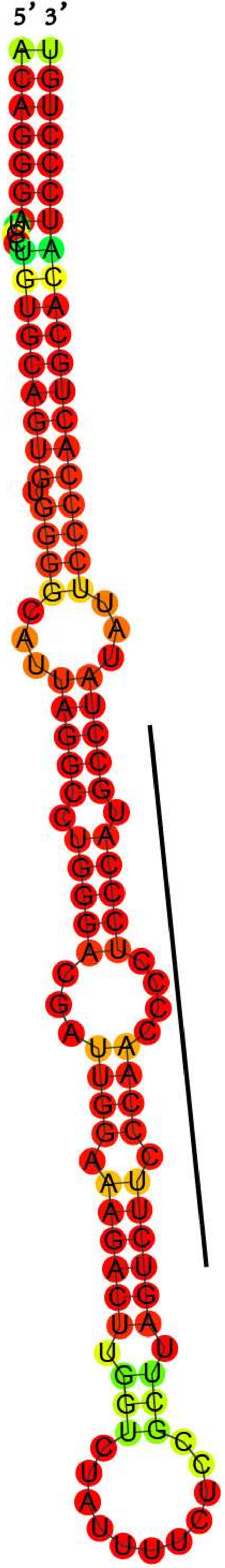

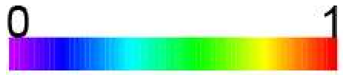

pab-MIR482o

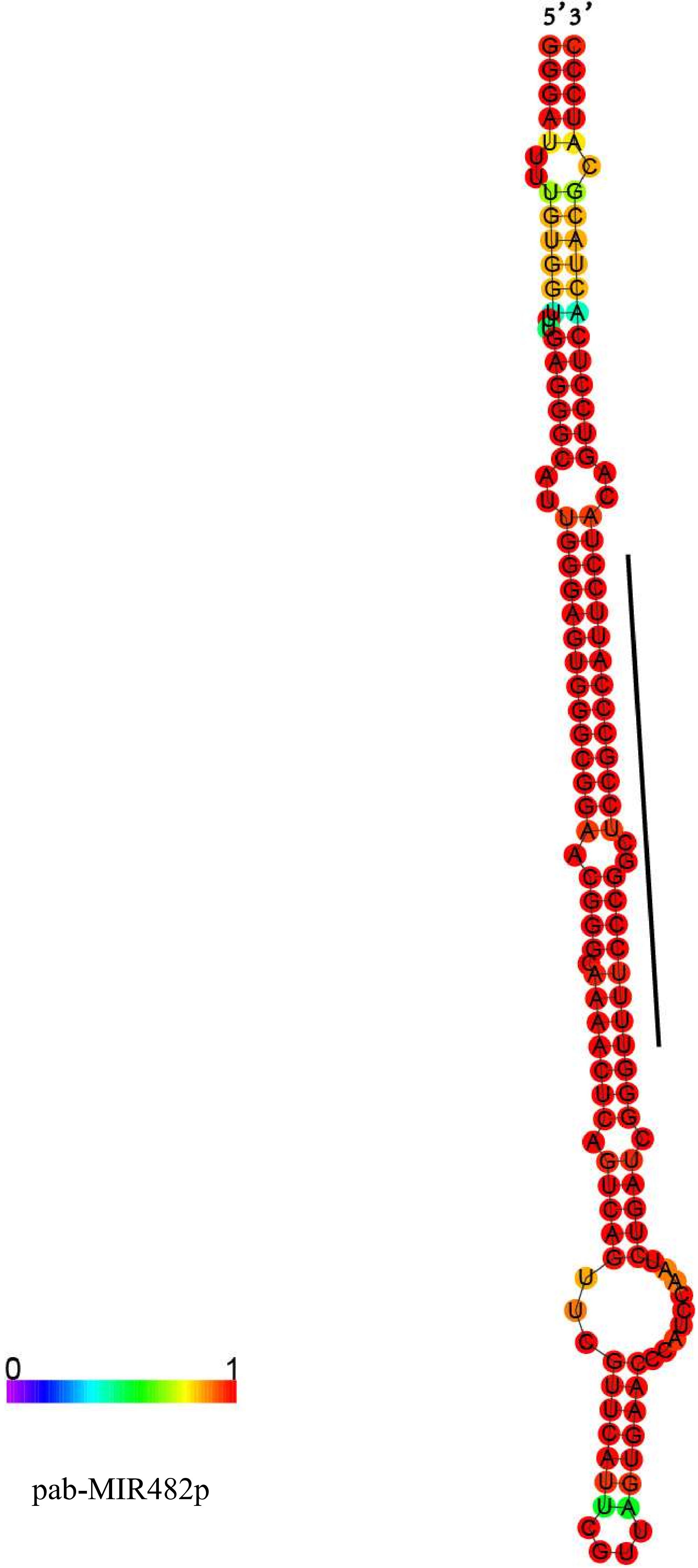

pab-MIR482p

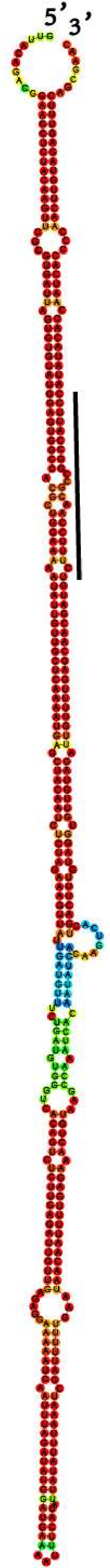

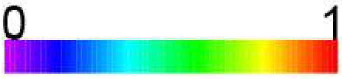

pab-MIR482q

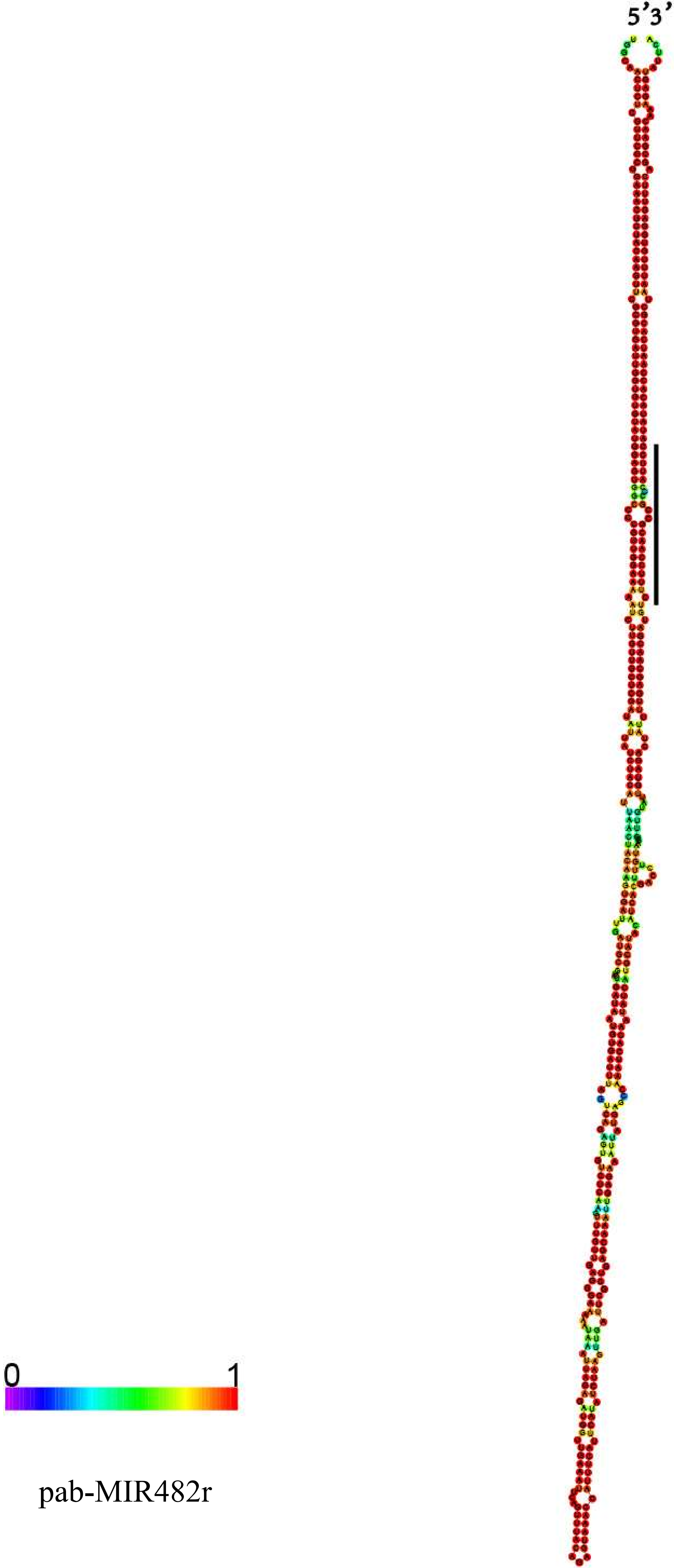

pab-MIR482r

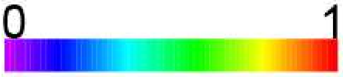

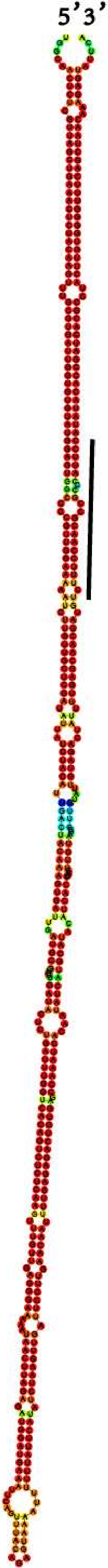

pab-MIR482s

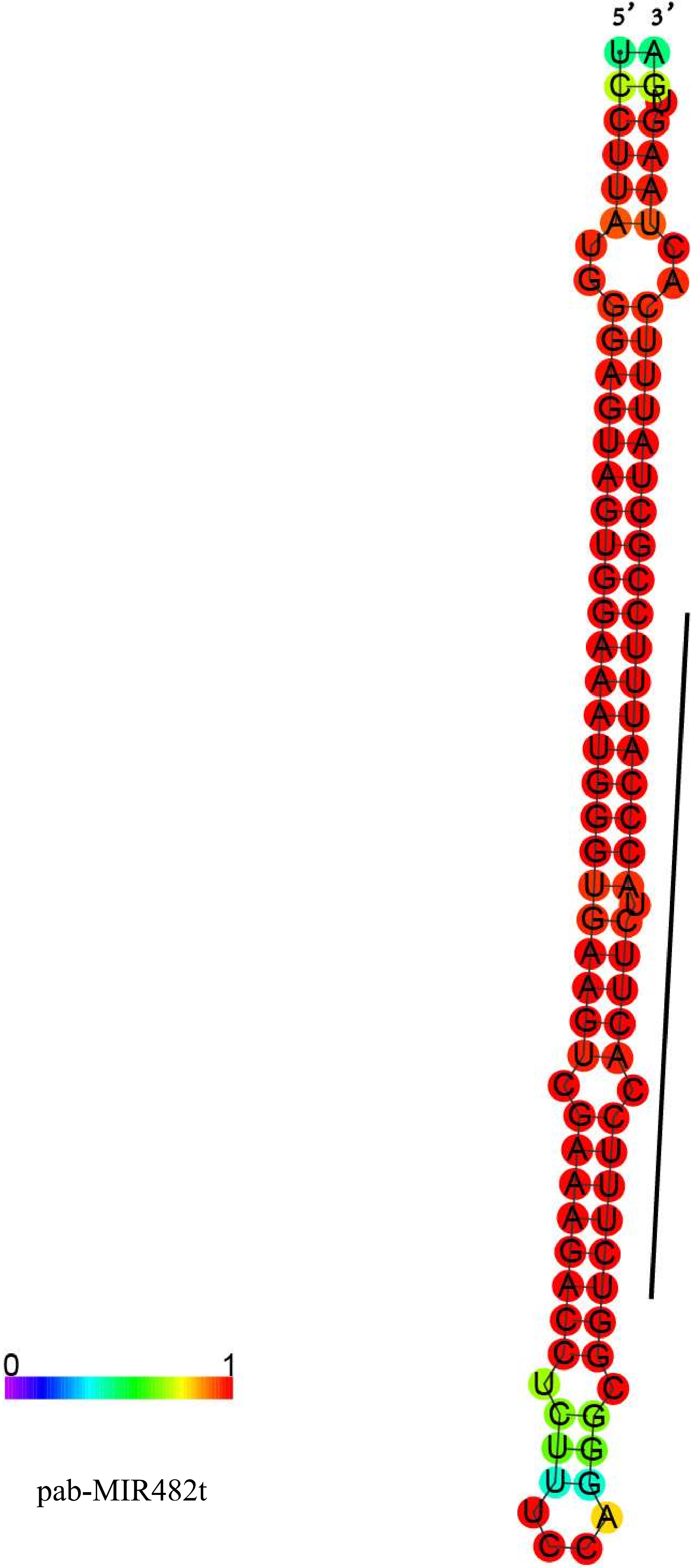

pab-MIR482t

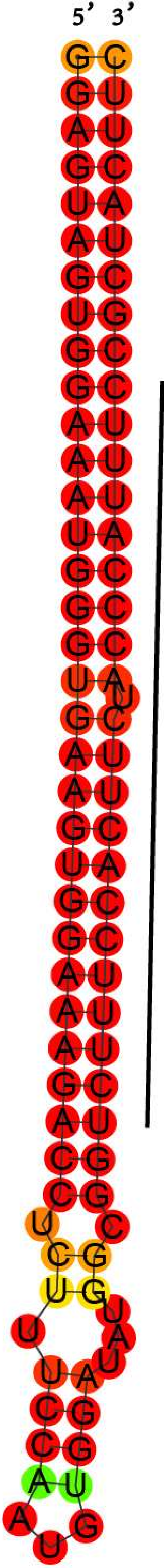

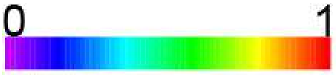

pab-MIR482u

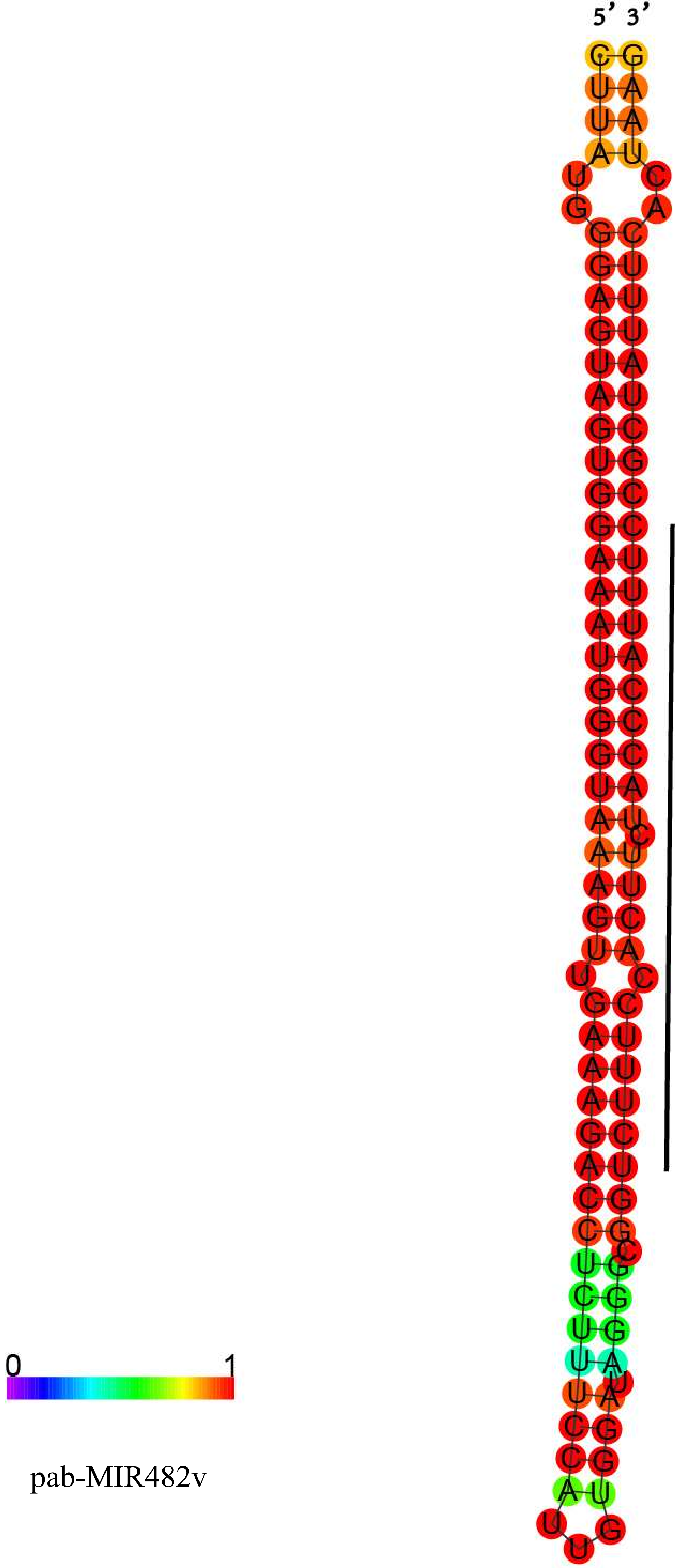

pab-MIR482v

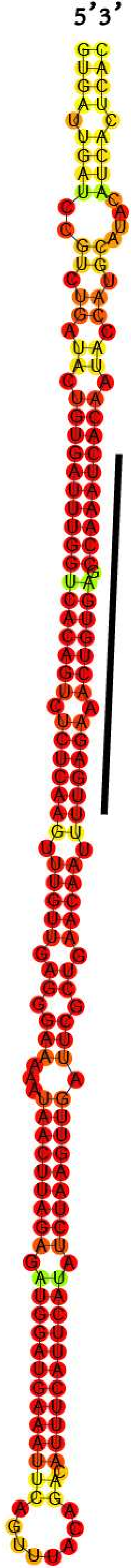

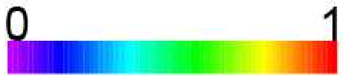

pab-MIR482w

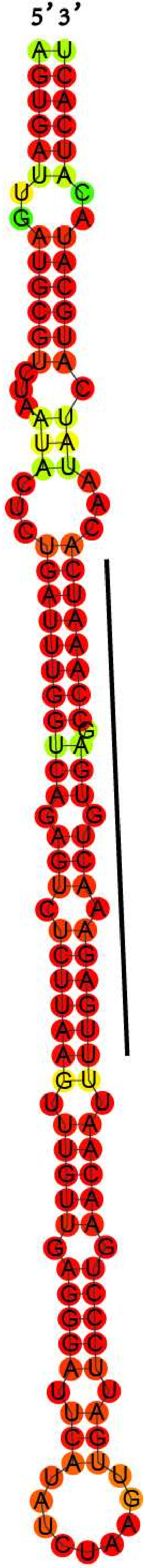

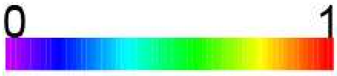

pab-MIR482x

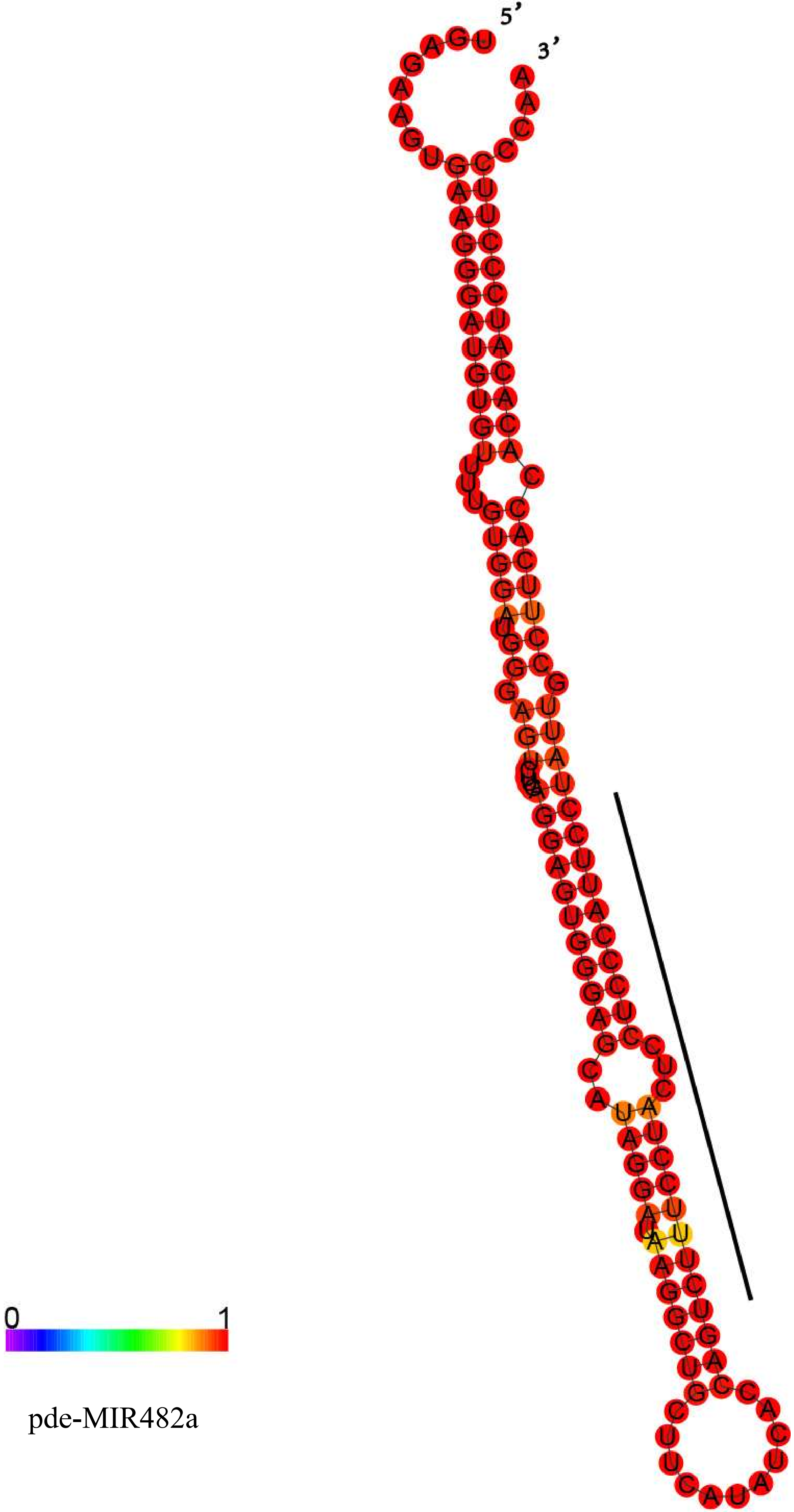

pde-MIR482a

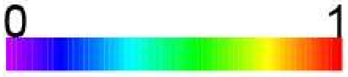

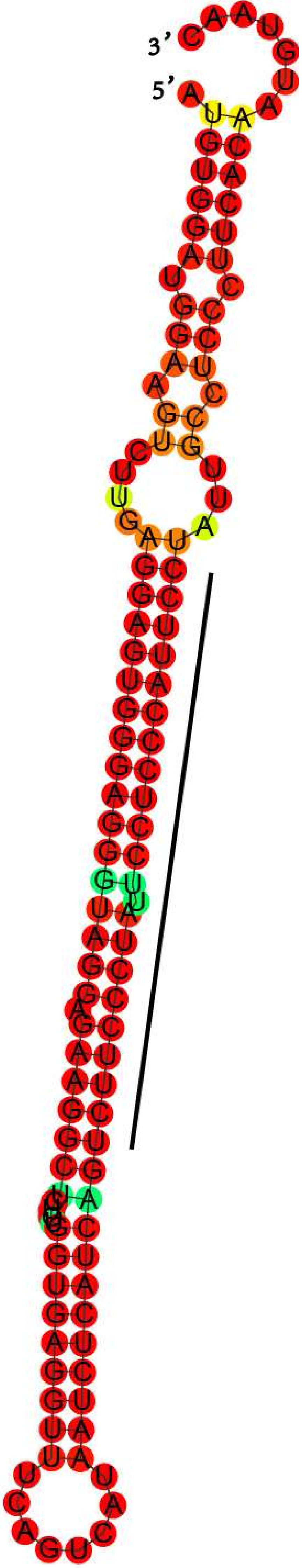

pde-MIR482b

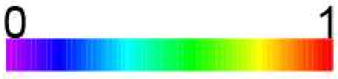

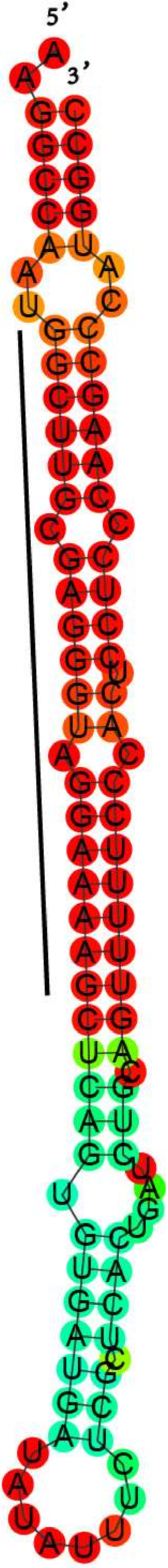

pde-MIR482c

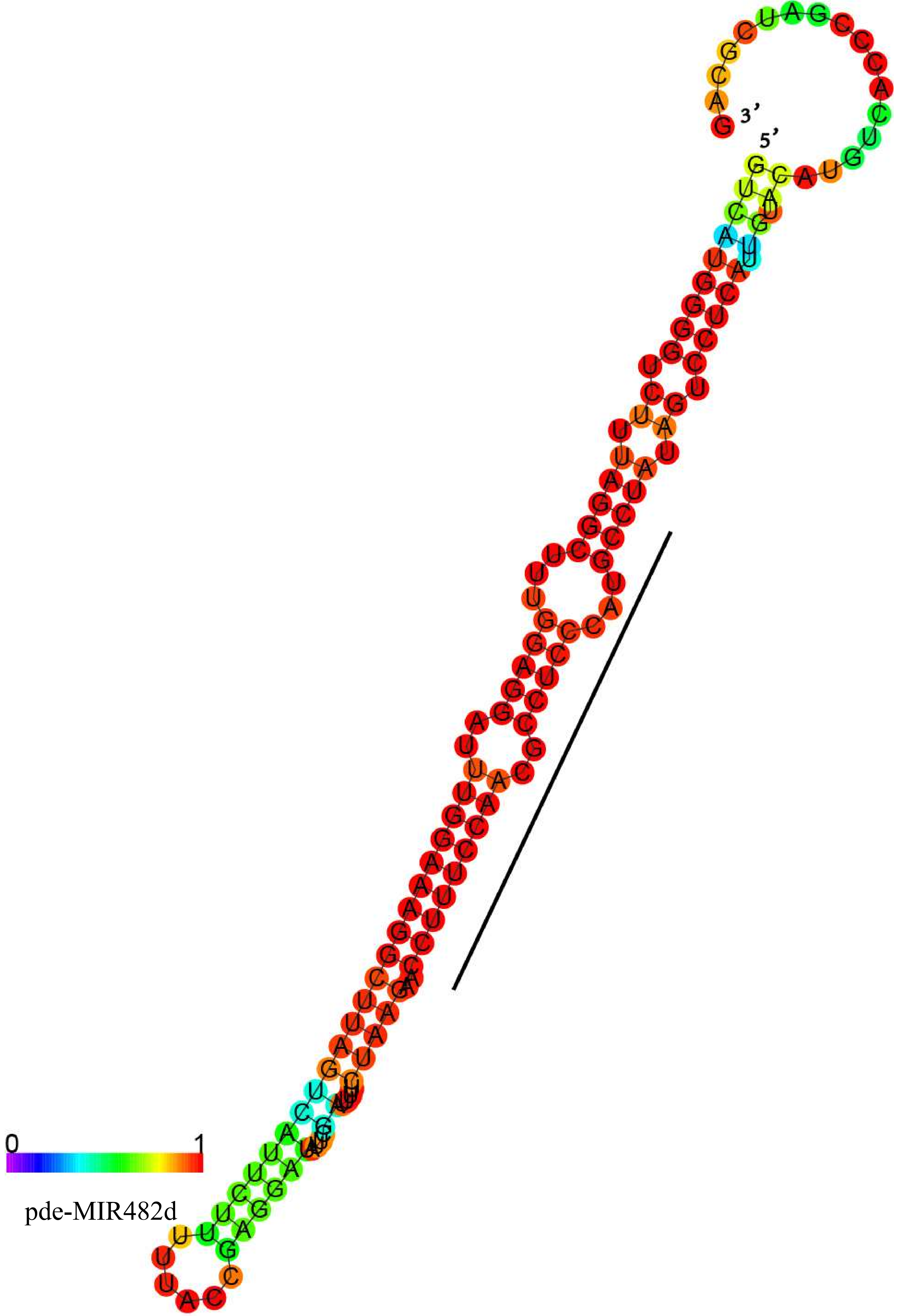

pde-MIR482d

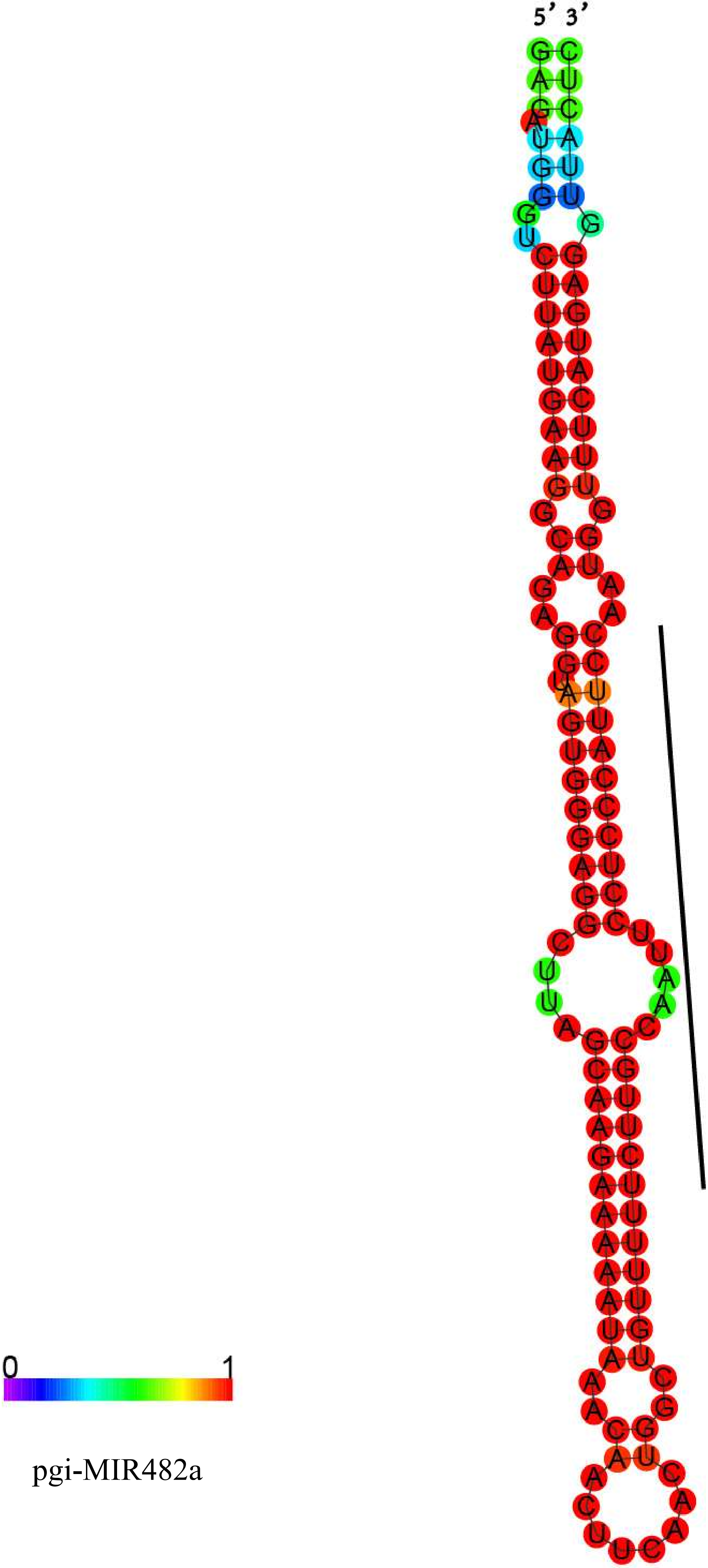

pgi-MIR482a

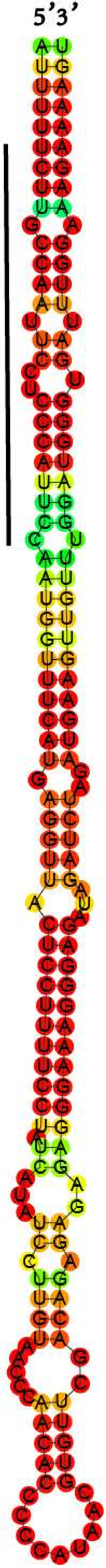

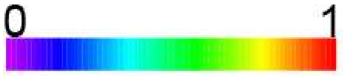

pgi-MIR482b

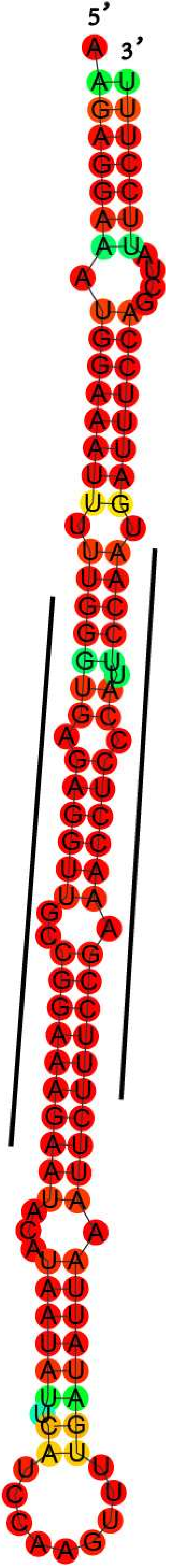

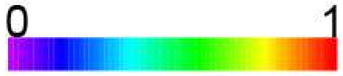

ppe-MIR482a

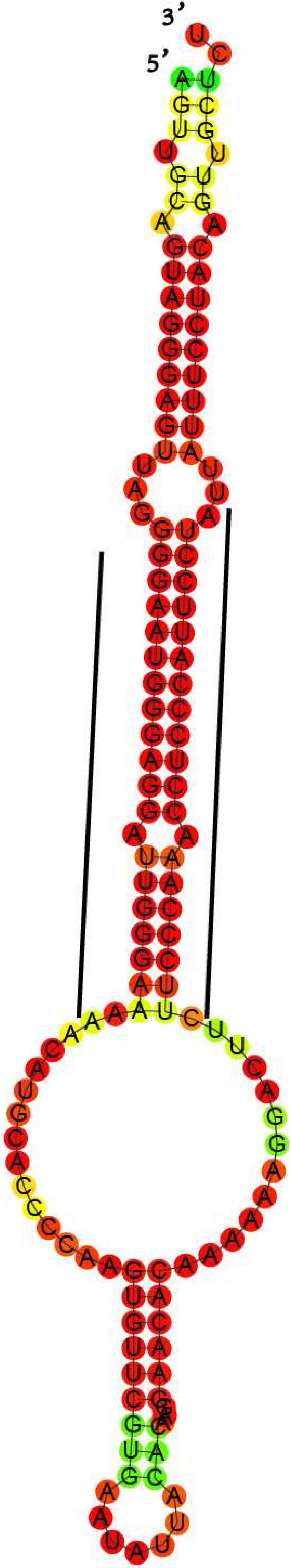

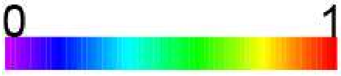

ppe-MIR482b

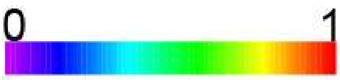

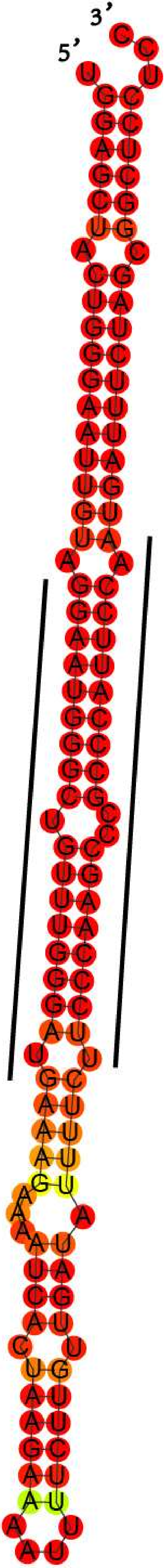

ppe-MIR482c

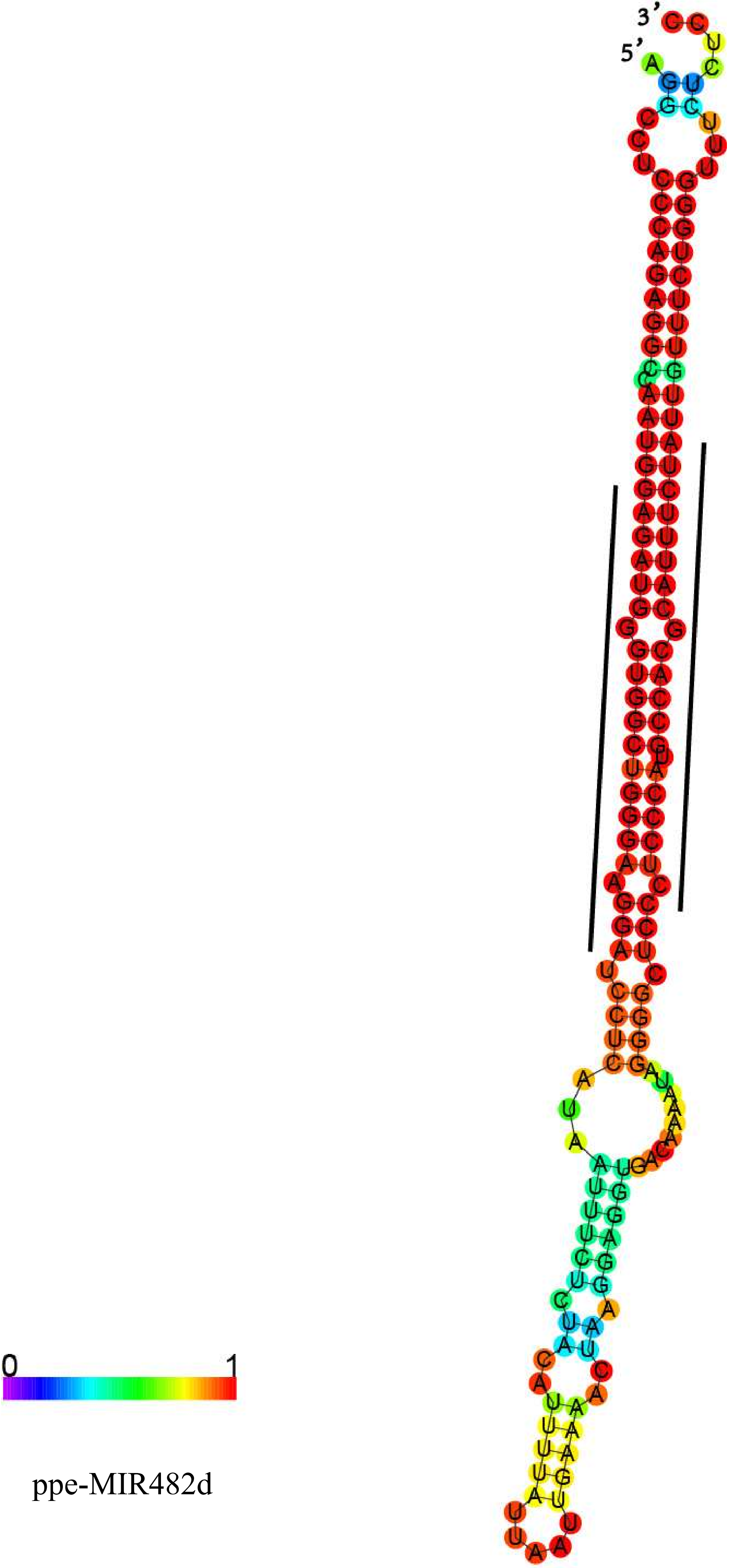

ppe-MIR482d

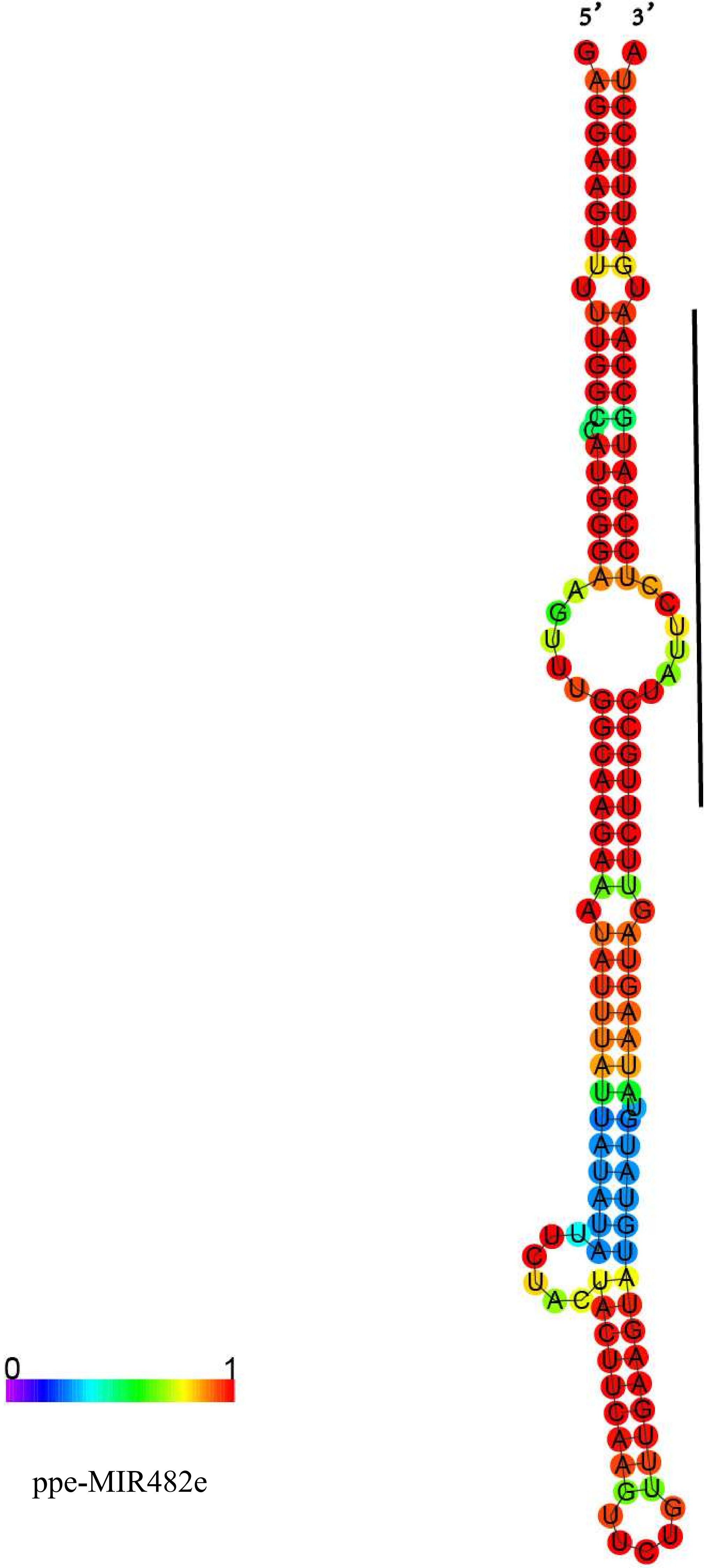

ppe-MIR482e

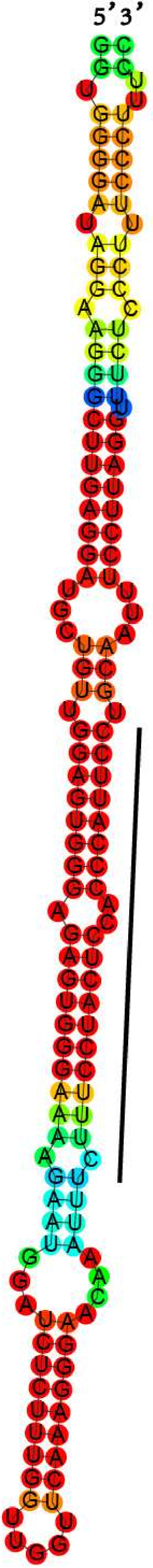

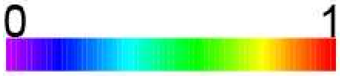

ppe-MIR482f

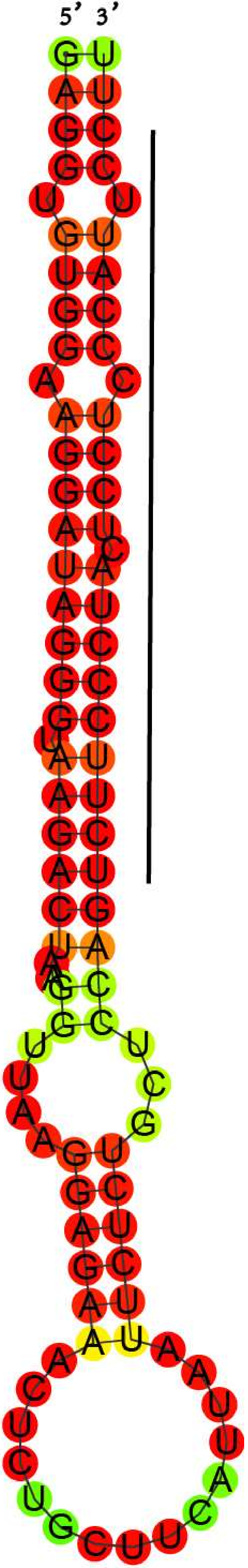

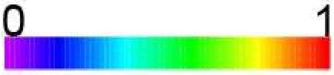

pta-MIR482a

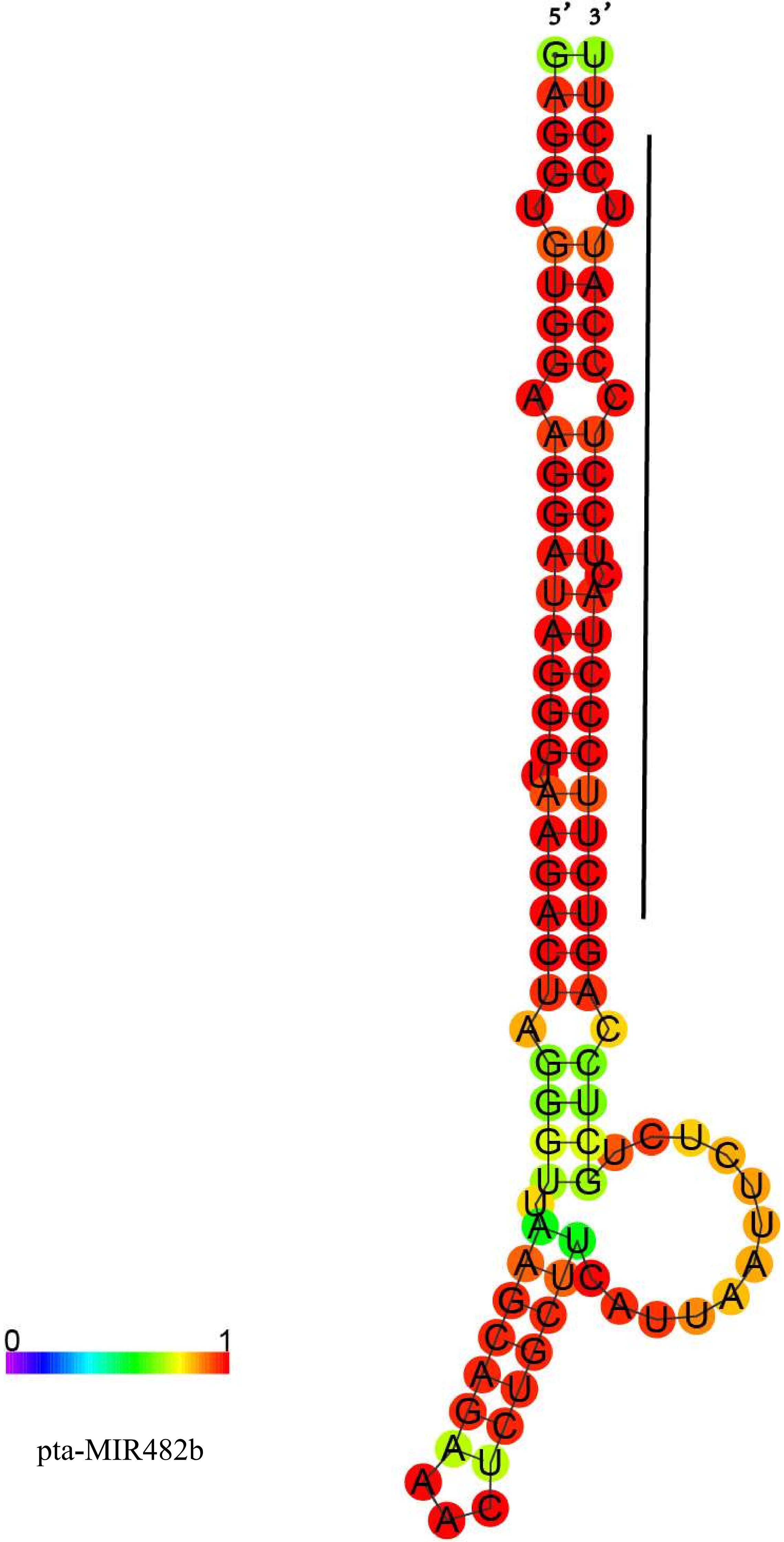

pta-MIR482b

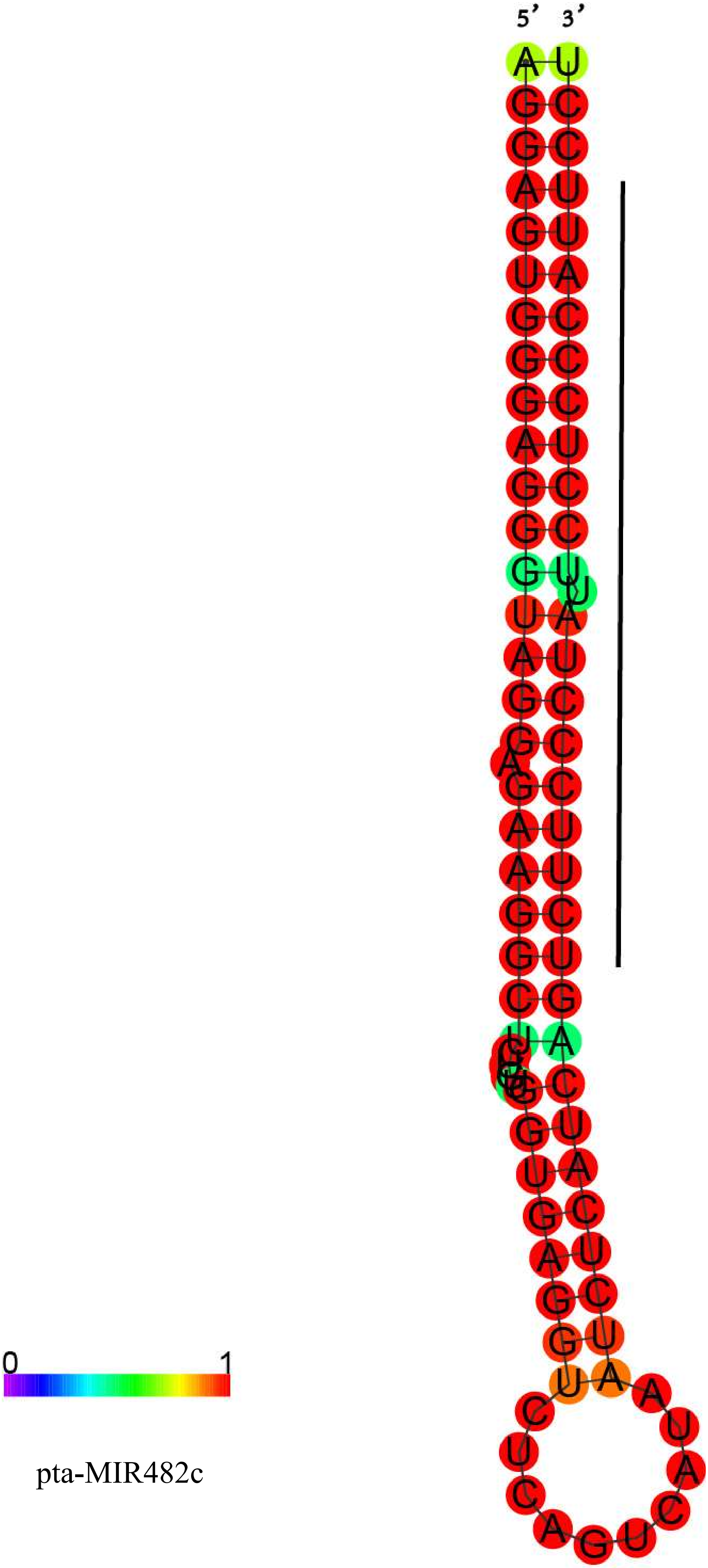

pta-MIR482c

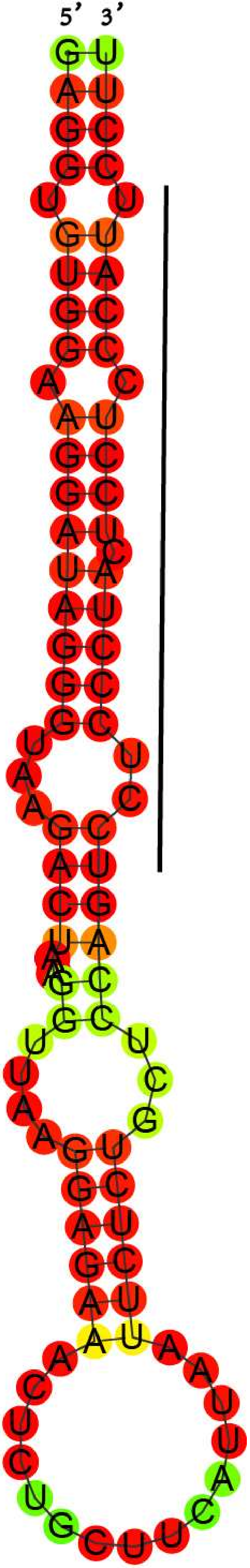

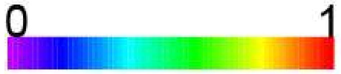

pta-MIR482d

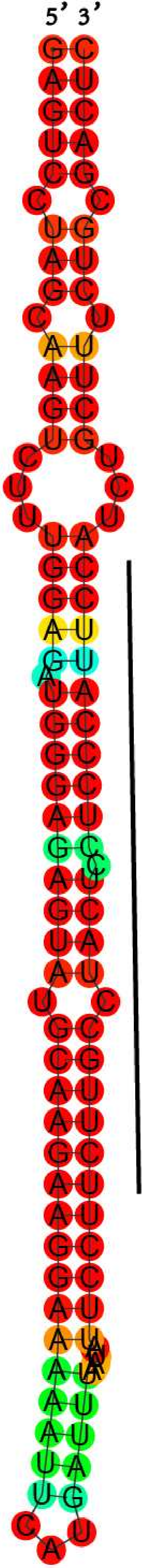

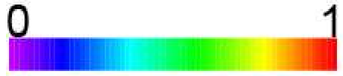

ptc-MIR482a

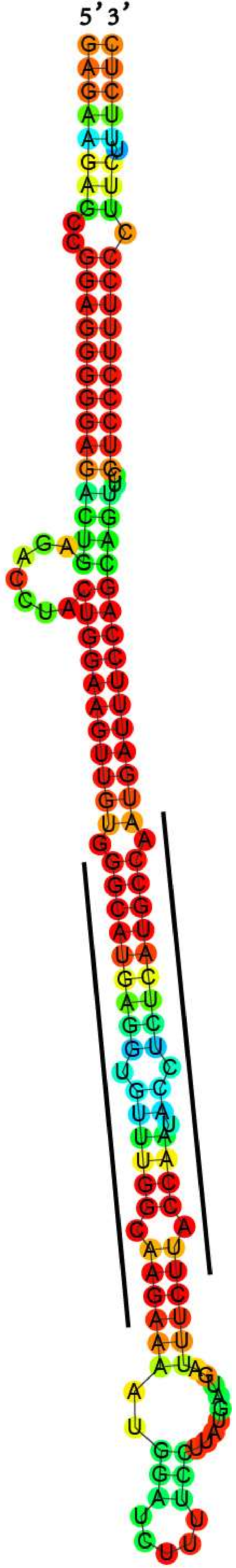

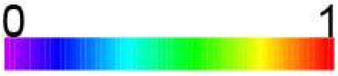

ptc-MIR482b

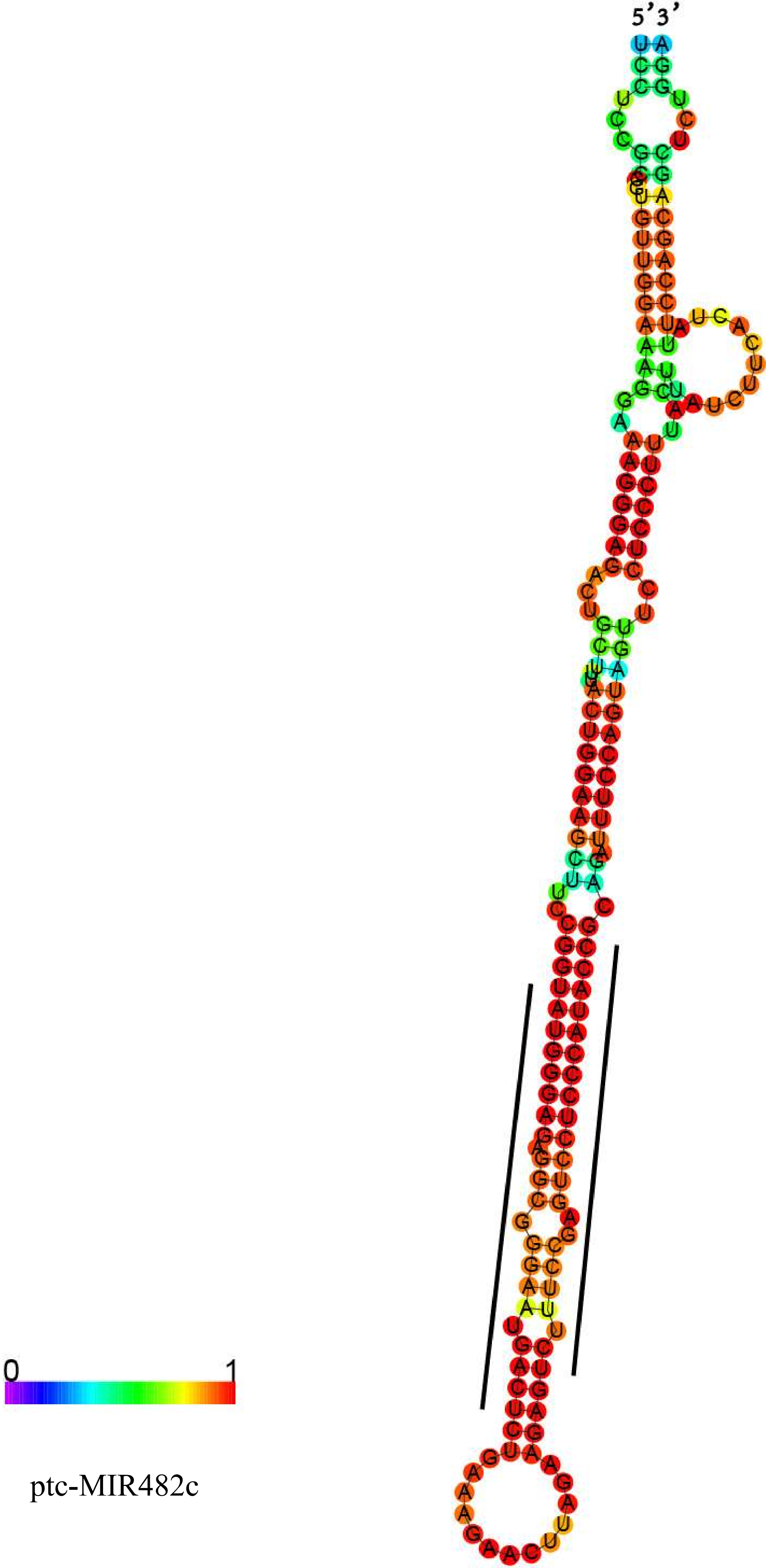

ptc-MIR482c

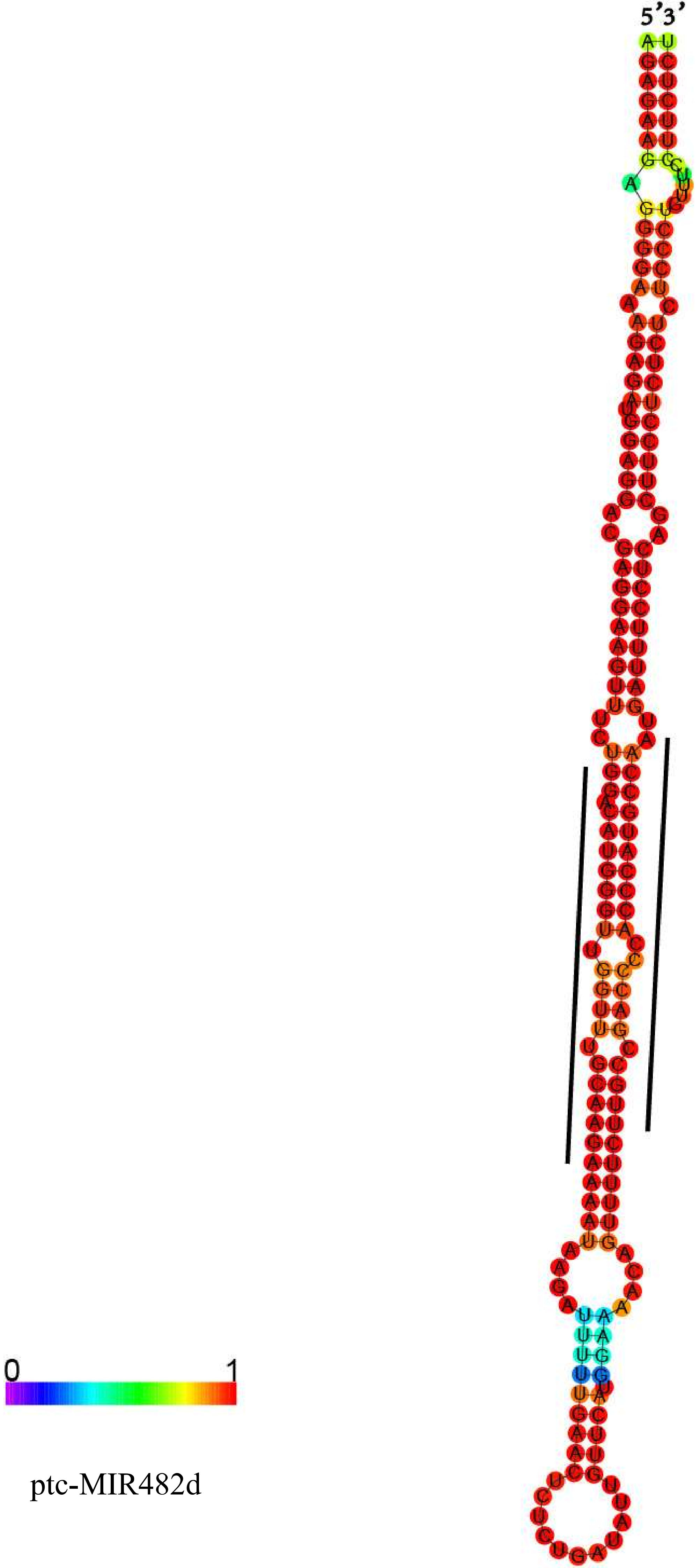

ptc-MIR482d

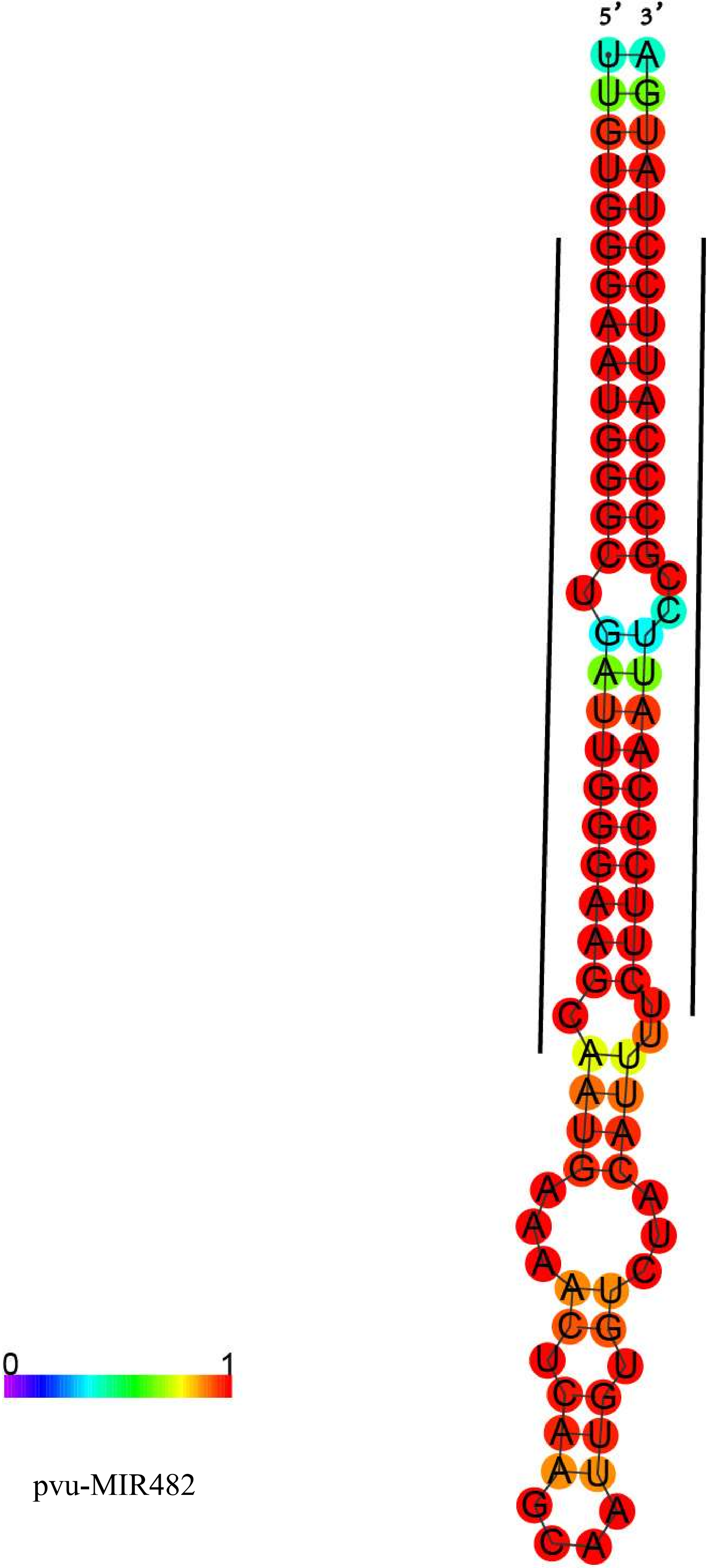

pvu-MIR482

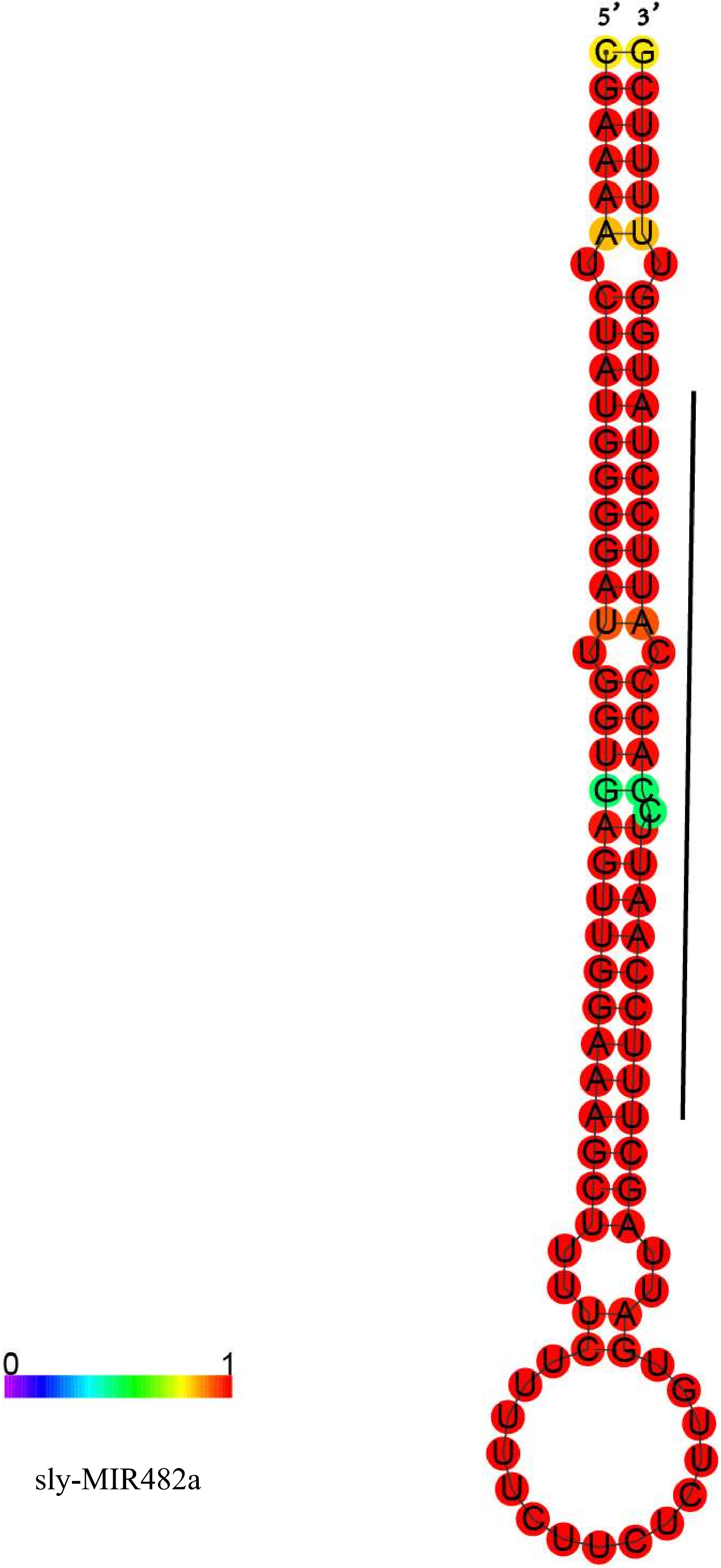

sly-MIR482a

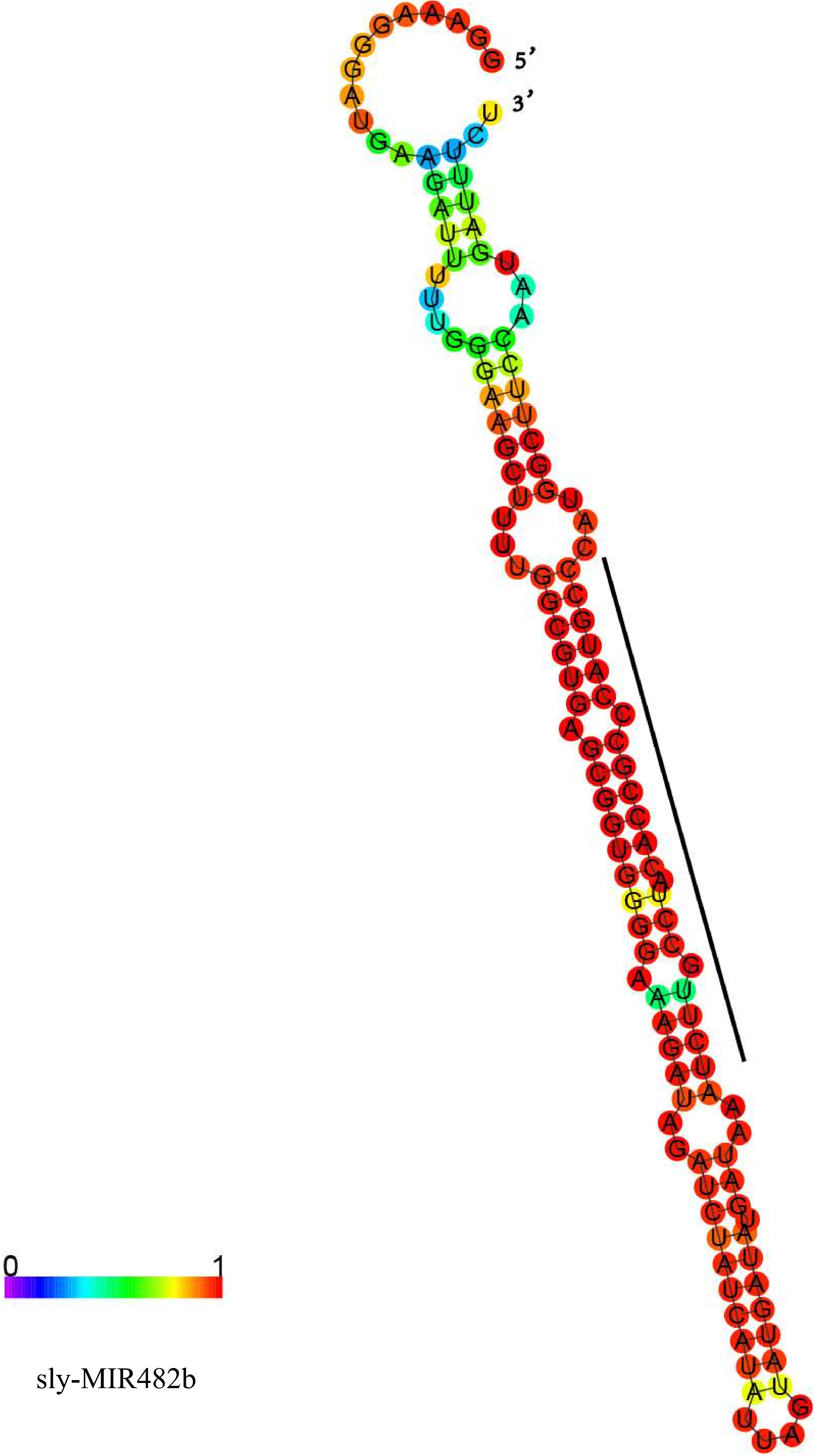

sly-MIR482b

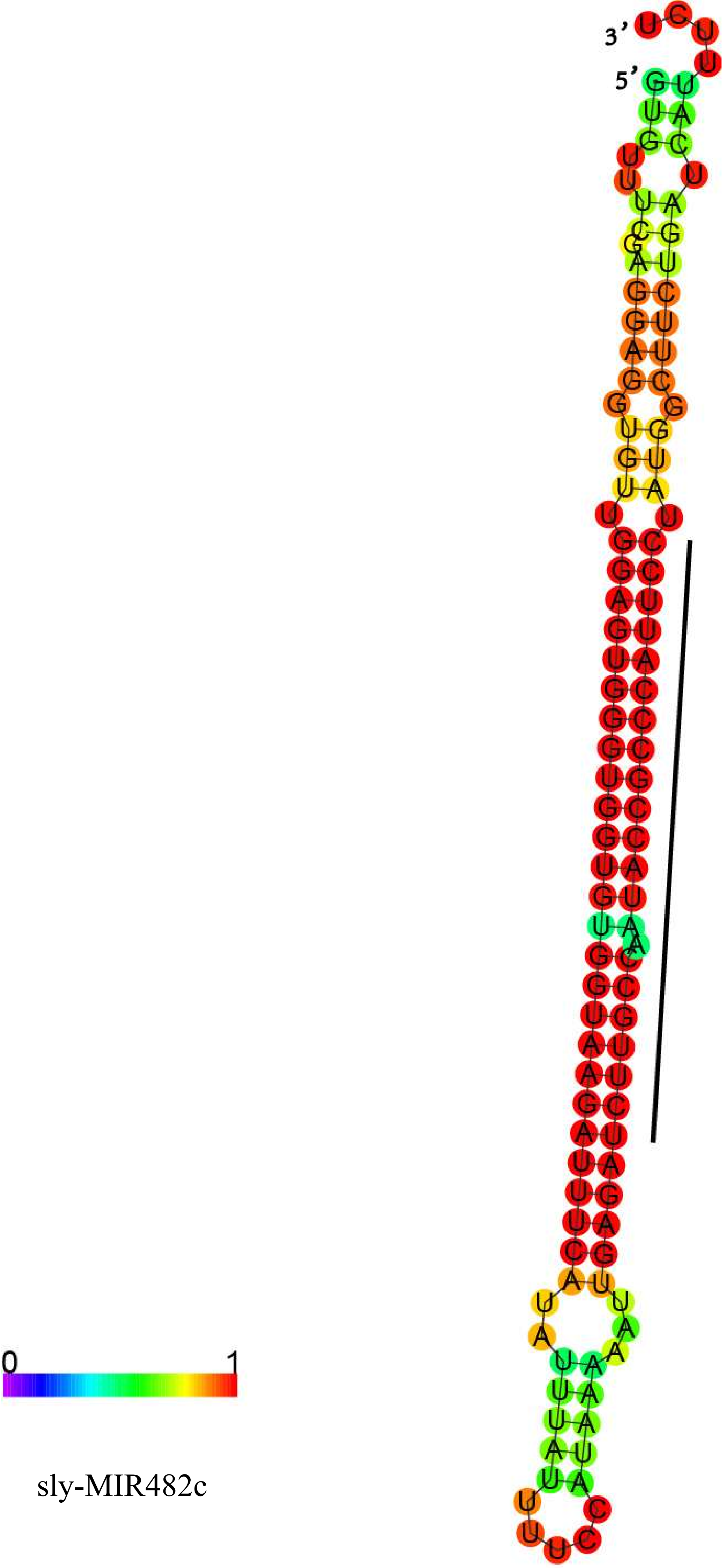

sly-MIR482c

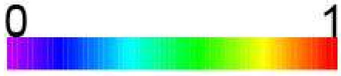

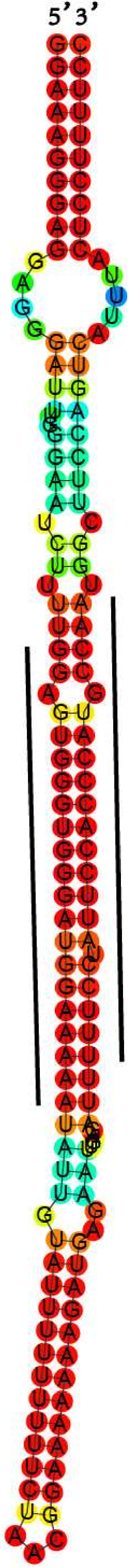

sly-MIR482d

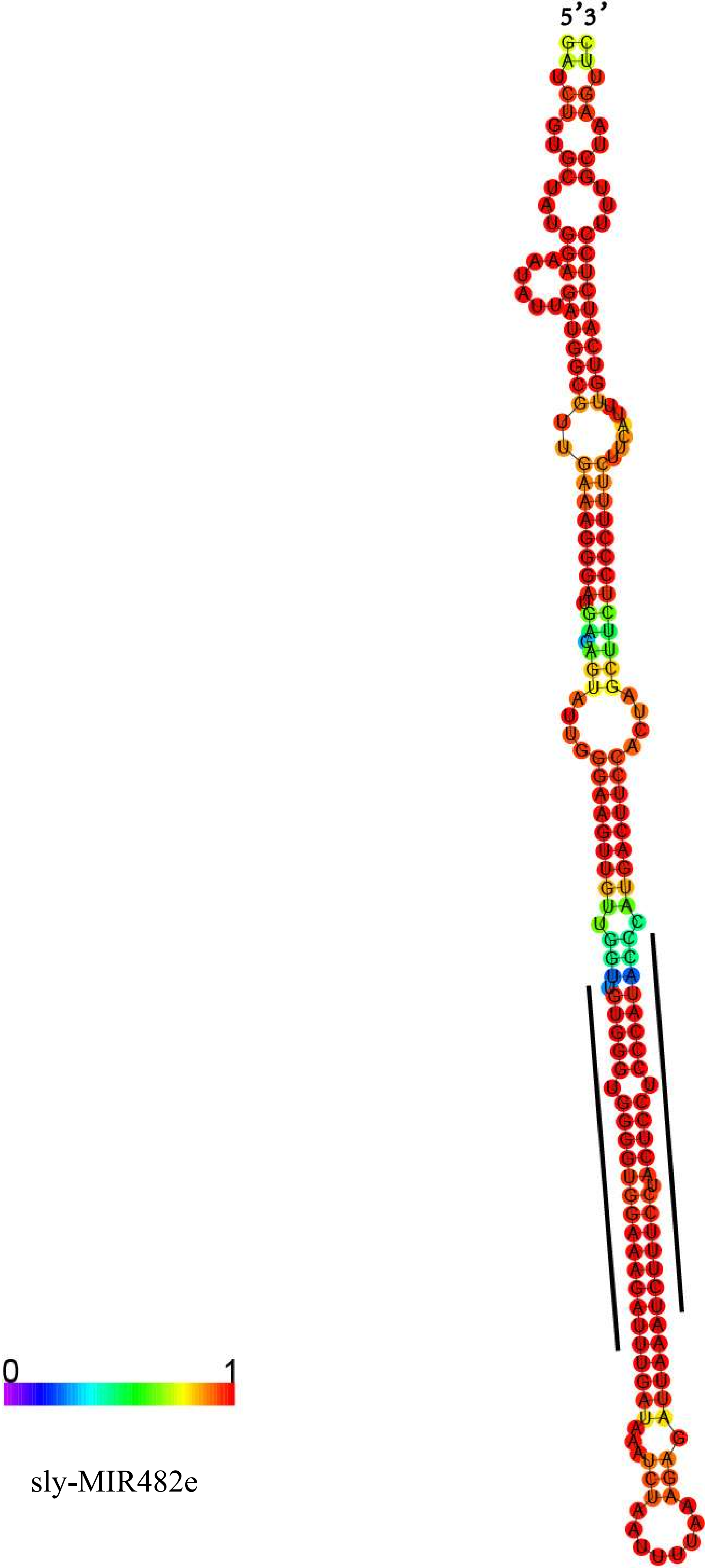

sly-MIR482e

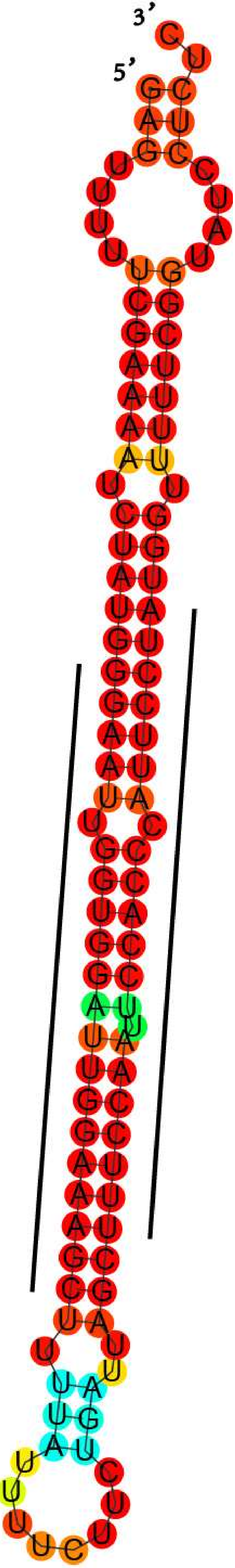

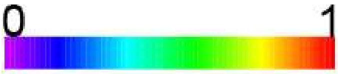

stu-MIR482a

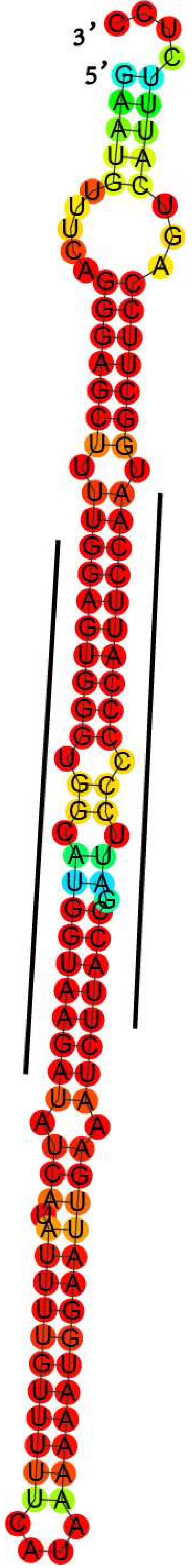

stu-MIR482b

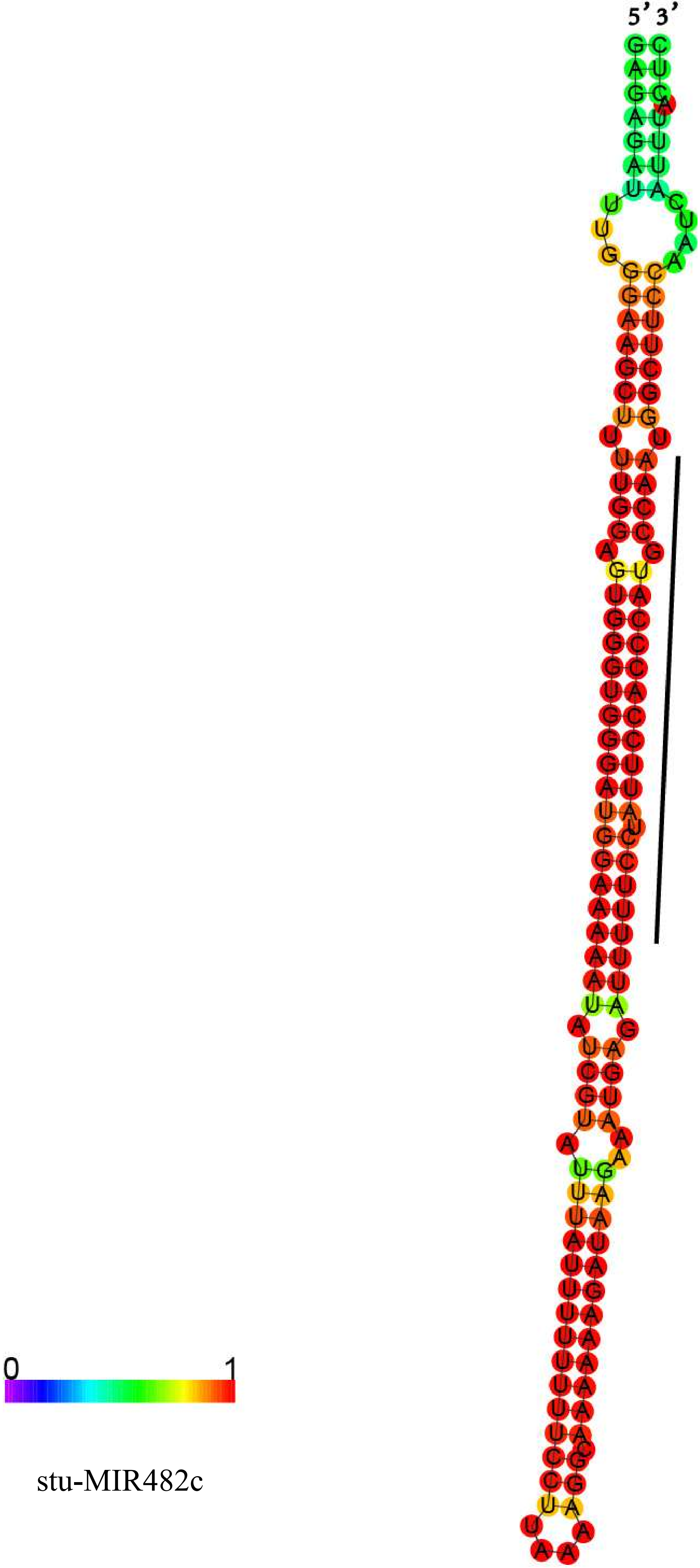

stu-MIR482c

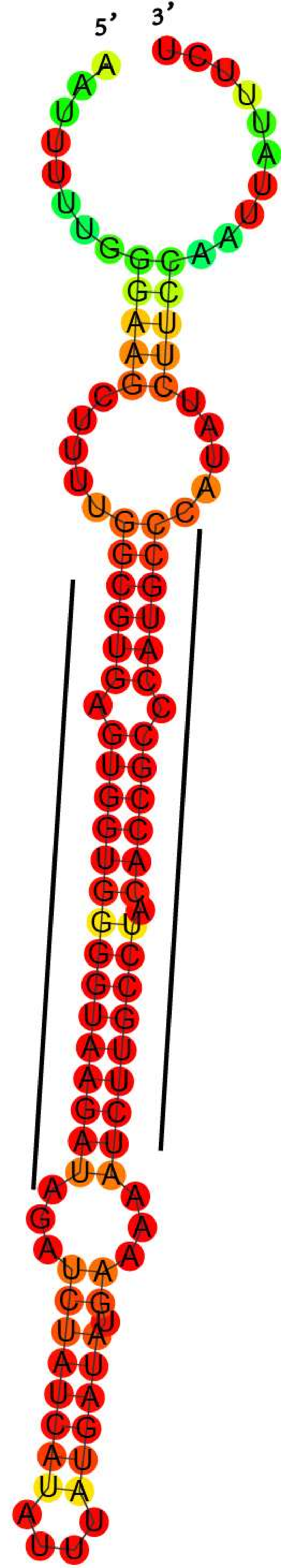

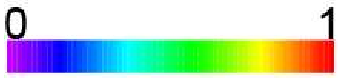

stu-MIR482d

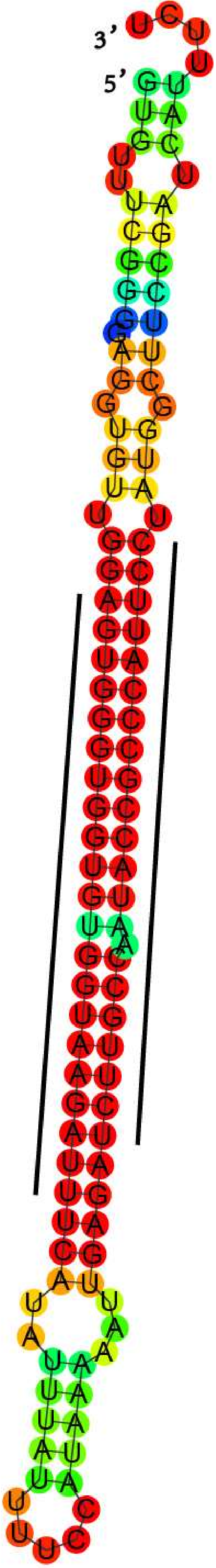

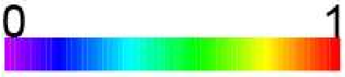

stu-MIR482e

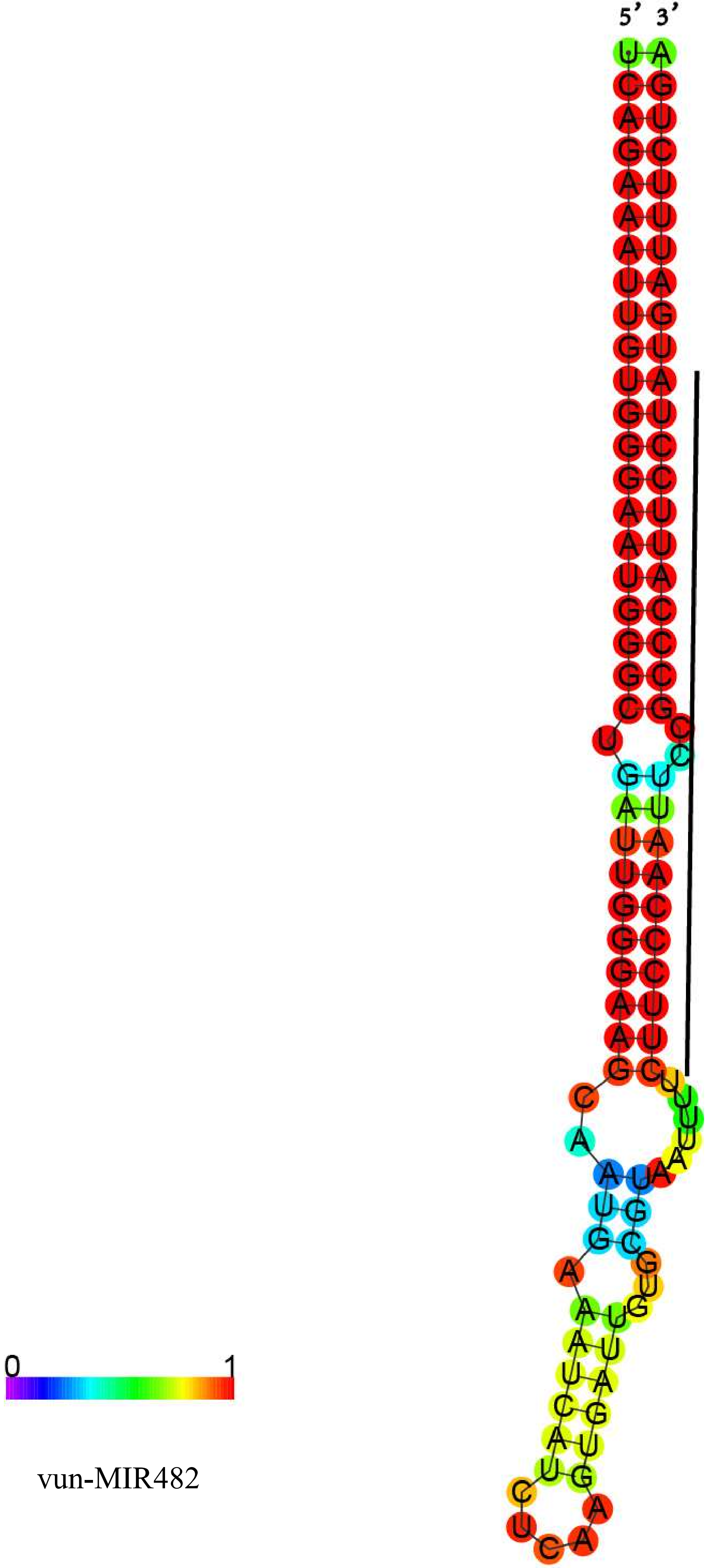

vun-MIR482

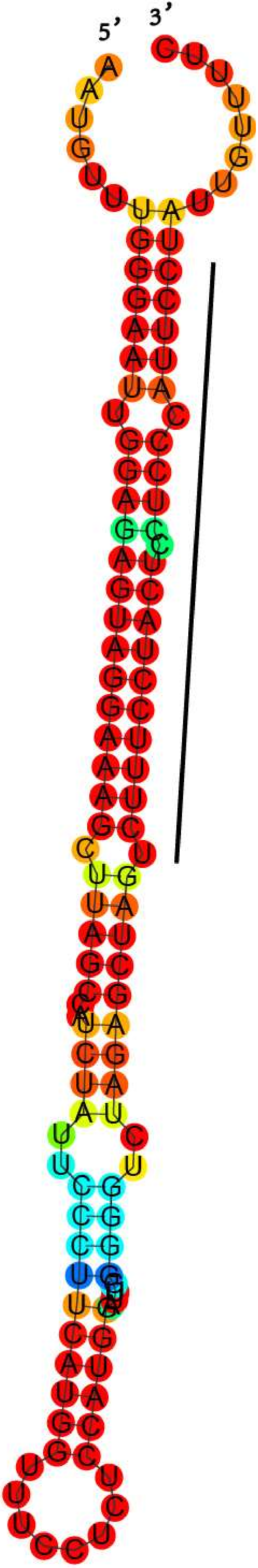

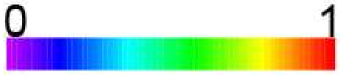

vvi-MIR482

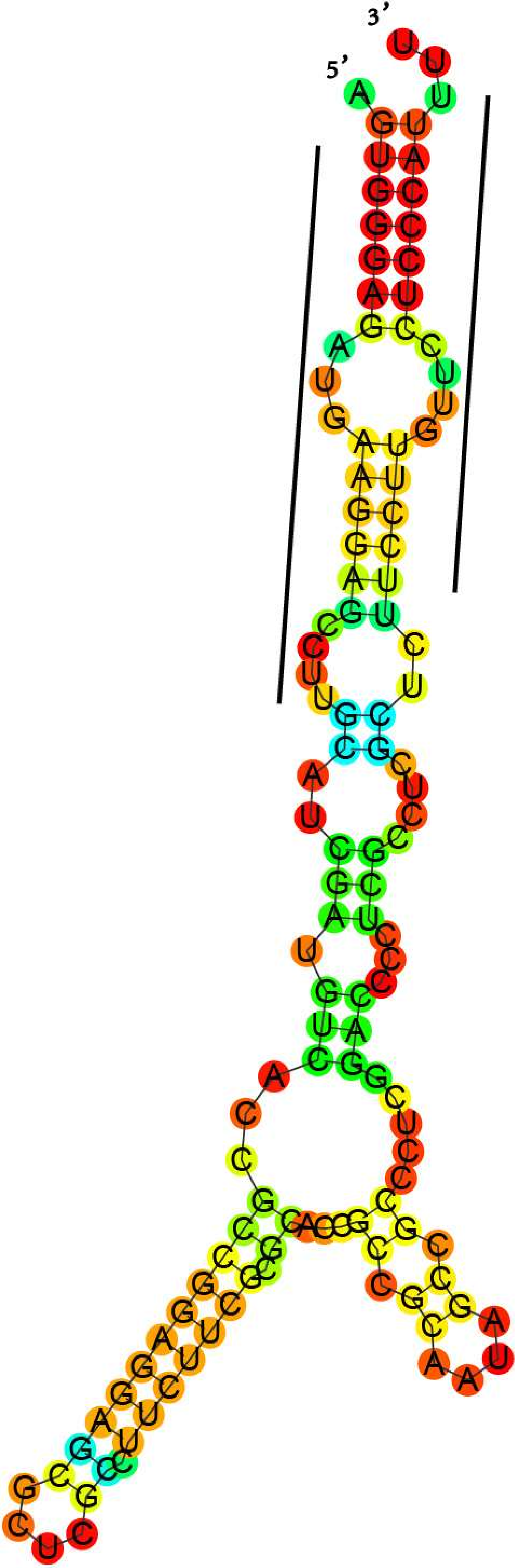

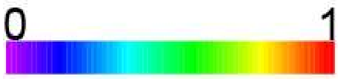

zma-MIR482

Figure S1: Secondary structure of miR482 precursor sequences in plants.

Note:The mature miRNAs sequences were marked with black underline
