# Supplementary material for: Analysis of the miR482 Gene Family in Plants
Source: Genes (Basel). 2024 Aug 8;15(8):1043. doi: 10.3390/genes15081043 (PMC11353999; doi:10.3390/genes15081043)
Supplement: Supplementary file 1 [file genes-15-01043-s001.zip › Supplementary Materials/Supplementary Materials-Table S1.docx]

**Supplementary Materials**

**Supplementary Table S1. The mature and precursor sequences of the miR482 family in plants.**

| **Precursor** | **Precursor sequence** | **MiRNA** | **MiRNA sequence** |
| --- | --- | --- | --- |
| aof-MIR482b | GAGGACUAGAUUGUUAGGUGUUAUUGGAUUGGGUUAGGAUUGGAAAAAUCUUAUGAAUCCAAGUAUUUUUUCCAAAACCUCCCAUUCCGAUGAUUUCUAACUGGCUAGCCCUCUC | aof-miR482b | UUUCCAAAACCUCCCAUUCCGA |
| aof-MIR482c | GGAAGAUGGGUUUCUUGGAAUUAUGGGAGUGGGCGGAUAGGCUAAAAAUAUCGAAAUGAUCCCCUGAUCAUCGAUAUUAUUGUAAUUCUUUUUGCCUACUCCGCCCAUUCCCCUGAUCUCAAGUCUCAUUUUUCCCCG | aof-miR482c | UUGCCUACUCCGCCCAUUCCCC |
| aqc-MIR482a | GGGGAUGGUGGAAAGUGAUUGAGUUAUGAGAAGCCUUGGGUAUGAGAGGGUCGGCAUGGUAUGAUGAUGAUGAUGAUGAUGAUGAUGAUGAUUCUUCUUUCAUUUUCUCUAUUUAAUCUUGCCGACUCCUCCCAUACCAAUGGUUUCUCAUUUCUCUUCCCUUUCCUUUUUAUCCCCCU | aqc-miR482a | UCUUGCCGACUCCUCCCAUACC |
| aqc-MIR482b | GGAUUGAGUUAUGAGAAGCCUUGGGUAUGAGAGGGUCGGCAUGGUAUGAUGAUGAUGAUGAUGAUGAUGAUGAUGAUUCUUCUUUCAUUUUCUCUAUUUAAUCUUGCCGACUCCUCCCAUACCAAUGGUUUCUCAUUUCUCUUCCCU | aqc-miR482b | UCUUGCCGACUCCUCCCAUACC |
| aqc-MIR482c | GGGGAUGGUGGAAAGGGAUUGAGUUAUGAGAAGCCUUGGGUAUGAGAGGGUCGGCAUGGUAUGAUGAUGAUGAUGAUGAUGAUGAUGAUGAUUCUUCUUUCAUUUUCUCUAUUUAAUCUUGCCGACUCCUCCCAUACCAAUGGUUUCUCAUUUCUCUCCCCUUCCUUUUUUCCCCCU | aqc-miR482c | UCUUGCCGACUCCUCCCAUACC |
| aof-MIR482a | GGGAGUCGGAUUGUUAGGAGUUAUCGGAUUGGGGUUGGAUUGGAAAAAUCUUUUUAAUGCCUAAAAUUAUUUUCCAAUGCCUCCCAUUCCGGUGAUUUCUAACCGUCUAUUCCUCUC | aof-miR482a | UUUCCAAUGCCUCCCAUUCCGG |
| csi-MIR482a | AGGAAGUUUUGGGAGUGGGAGCGUGGGGUAAGAAGUGAAGAAAACGUAUAAUUUUUCUUUUCUUUUUCAAUUGAGAAAGAAAAUUACAAAUUCAAUUUCUUCCCUAUGCCUCCCAUUCCUAUGAUUUCC | csi-miR482a-5p | AGUGGGAGCGUGGGGUAAGAAG |
|  |  | csi-miR482a-3p | UCUUCCCUAUGCCUCCCAUUCC |
| csi-MIR482b | AGGAAAUUUCUGGAAUGGGAGGCUUGGCAAGAAGCGAUCUAUUUUUCUUGUUCAAUAAAAAAGAAAAAGAAAAUUUUUCUUGCCCACCCCUCCCAUUCCUUGGAUUUCC | csi-miR482b-5p | AAUGGGAGGCUUGGCAAGAAG |
|  |  | csi-miR482b-3p | UCUUGCCCACCCCUCCCAUUCC |
| csi-MIR482c | UAAAAUUUUUGGAAUUGGGUGCUAGGGAAGGUUUUGUUUGACUUGUCUGUCAUAACUAAAUUAAUUUUCCCUAGUCCCCCUAUUCCUAUGAUUUUC | csi-miR482c-5p | GGAAUUGGGUGCUAGGGAAGG |
|  |  | csi-miR482c-3p | UUCCCUAGUCCCCCUAUUCCUA |
| csi-MIR482d | AGAGGGAGUUGAAGGAUAUUGGGAAGGUUUUGGUAUGGGUGAGUAGGGAAGAUAAUUUAACUAAGUAAUAAUAAUAUCACUUUUUUCAUUAUUACUAUUUACUUAAGGAAUUUGUAUAUCUUCCCUACUCCACCCAUGCCAUAGGUUUCCCAUUAUUCCUCCCUCUCCUUAUC | csi-miR482d-5p | UGGUAUGGGUGAGUAGGGAAG |
|  |  | csi-miR482d-3p | UCCCUACUCCACCCAUGCCAUA |
| csi-MIR482e | GGAAAGGGCUGAGGCCCGUAAGACAUUUUCGGUCAUGGGAGGAUUGGCGAAAAAAUCUUUCAAUUUUACCAUAAAGUAUAUAAGAUGACGAUGAUGAUUAAAUGAAAUCUUUUGCCAACUCCUCCCAUGCCGAUGAUUUCUUAUGGUUUCAAUUCUUUCCAAU | csi-miR482e-5p | GGUCAUGGGAGGAUUGGCGA |
|  |  | csi-miR482e-3p | UUGCCAACUCCUCCCAUGCCGA |
| csi-MIR482f | GGGAAGUCUUGGAGAUGGGUGAGUUGGGAAAAUAAUAACUUUCUUCACGAAAGAAAGCAAUUUUUUUCCCACACCUCCCAUCCCUAAGAUUUUC | csi-miR482f-5p | GAUGGGUGAGUUGGGAAAAUA |
|  |  | csi-miR482f-3p | UUUUCCCACACCUCCCAUCCC |
| csi-MIR482g | AGGAAGUUUUGGGAGUGGGAGCGUGGGGUAAGAAGUGAAGAAAACGUAUAAUUUUUCUUUUCUUUUUCAAUUGAGAAAGAAAAUUACAAAUUCAAUUUCUUCCCUAUGCCUCCCAUUCCUAUGAUUUCC | csi-miR482g-5p | AGUGGGAGCGUGGGGUAAGAAG |
|  |  | csi-miR482g-3p | UCUUCCCUAUGCCUCCCAUUCC |
| eun-MIR482a | GGGAAAGGGAGAGAGAGUCUCAGGAAGUUGUGGGCAUGGGUUGUUUGGUGAGAGGCAUCGCUCUCUAUUCUCAUGCGCGCAUGGAGGGAGGUGGGUGAGGUUUCUUGCCAAUACCACCCAUGCCAGCAAUUUCCUGCUUUUCCUCCCUUUCCC | eun-miR482a-5p | CAUGGGUUGUUUGGUGAGAGG |
|  |  | eun-miR482a-3p | UCUUGCCAAUACCACCCAUGCC |
| eun-MIR482b | GGGGAAAGAAAGAUGCAGCGUGAGGGAGUGCAUGAAAUGGGAGGGUGGGAAAGACGGUGAGUCGAGCGUGUGUCAUGGCGUUCUGCUCUUUUUCCUAUUCCUCCCAUUCCAUACACUCCAUCGCCUCCUUCCUUUCCCC | eun-miR482b-5p | GAAAUGGGAGGGUGGGAAAGA |
|  |  | eun-miR482b-3p | UUUCCUAUUCCUCCCAUUCCAU |
| eun-MIR482c | GAGGGAAAGGGAGAUUCGAGCUACCGGAAGUUGUGGGAAUGGGCGGUUUGGGAUAAACCGGAGAUAACAAAGAUGUUGUUGAGAUCUCGGCUCGAUGAGUUCUUUCGAAUUAAGUUUUUUCCCAAGGCCGCCCAUUCCGACGAUUUCCGGCGGCUCCUCCCUUUCCCUU | eun-miR482c-5p | GAGAUUCGAGCUACCGGAAGUUGUG |
|  |  | eun-miR482c-3p | UUCCCAAGGCCGCCCAUUCCGA |
| fve-MIR482a | UGAGGAAGUUUCUGUCAUGGGAAGUUUGGAAAGAAUUGGUUGUUUGUUUAUGACAUCAAACUUCUUUCCAAUUCCUCCCAUGCCAGUGGUUUCUCA | fve-miR482a | UCUUUCCAAUUCCUCCCAUGCC |
| fve-MIR482b | GAUGGUGUGACAGAGAAGAGAAAGGAGUUGGGGGGAAAGGGAGGAGUGGCCGGAAGUUGUGGAGUGGAGUUCUGGGAAAGAAGACCAUGGUUUUUCUCGAAGUAAUUUGGGAAAAAAACAUUGGUUCUUUCCUAGUCCUGCCAUUCCUUGAUUUCCUGUCAUGCAUC | fve-miR482b | UCUUUCCUAGUCCUGCCAUUCC |
| fve-MIR482c | GGUAGACUUGUGAGGAAGUUGUAGUUGGGAUGGGAGGCUAGGGAAGAAAUUACUGGAAACUCAUCCUAAUUUCUUUCCUAUUCCUCCCAUCCCUCCUCAAUUUCCCAAGGUUUCUCC | fve-miR482c | UCUUUCCUAUUCCUCCCAUCCC |
| fve-MIR482d | GAAAGGUAGAUAGAUCCCAAGGAAGUUUUGGGGUAUAGGUGGGUAGGAUAGAGAUCAAAGACUUGUUCUUUCCCUGAACAGUUUCUCUUCCCUAUUCCACCUAUUCCCCAUGAGAUCCUUUGGUUCCUCCCUUUC | fve-miR482d | UUCCCUAUUCCACCUAUUCCCC |
| ghr-MIR482a | AUUGUUAAAGAGGGAAAGGGAGAAAGAGUUGCCGGGAGUUUAUGGAAGUGGGAUGGGUGGAAAGAUUUGUAUUUUCUCUCUUGAGUUUCUAAGAAUGUAAAGCAAAUCUUUCCUACUCCUCCCAUACCACUUAUUUCCAGUUCUUCCUCUCCACUUCUUACUUUUCUCGAU | ghr-miR482a | UCUUUCCUACUCCUCCCAUACC |
| ghr-MIR482b | AGAGACGAUGGAGAAGGAGCUUAGGAUUCUAGAAAGCUCGUGGGAUGGGUGAGGGGGUAAGACAAUGGACUGUUUCAGUCUUUUGGAAACAGAAAUAAAGAAGAAGGCUGGUUUAUGUCUUGCCUACUCCACCCAUGCCACUGGUUUCCUGGUGUUCCUCCUCCUCCUAAAGUCUCU | ghr-miR482b | UCUUGCCUACUCCACCCAUGCC |
| gma-MIR482a | UCAGAAUUUGUGGGAAUGGGCUGAUUGGGAAGCAAUGUGUGCUGGUGCAAUGCAUUUAAUUUCUUCCCAAUUCCGCCCAUUCCUAUGAUUUCUGA | gma-miR482a-5p | AGAAUUUGUGGGAAUGGGCUGA |
|  |  | gma-miR482a-3p | UCUUCCCAAUUCCGCCCAUUCCUA |
| gma-MIR482b | GGUAUGGGGGGAUUGGGAAGGAAUAUCCAUAAGCAAAAUAUGCUAUUUCUUCCCUACACCUCCCAUACC | gma-miR482b-5p | UAUGGGGGGAUUGGGAAGGAAU |
|  |  | gma-miR482b-3p | UCUUCCCUACACCUCCCAUACC |
| gma-MIR482c | AGAAUUUGUGGGAAUGGGCUGAUUGGGAAGUAAUGAGAUUGAGCAAUACAUUUAAUUUCUUCCCAAUUCCGCCCAUUCCUAUGAUUUCUG | gma-miR482c-5p | AUUUGUGGGAAUGGGCUGAUUGG |
|  |  | gma-miR482c-3p | UUCCCAAUUCCGCCCAUUCCU |
| gma-MIR482d | GGGGGAAGACAUGGGUAUGGGGGGAUUGGGAAGGAAUAUCCAUAAGCAAAAUAUGUUAUUUCUUCCCUACACCUCCCAUACCACUGUUUUUCCUC | gma-miR482d-5p | UAUGGGGGGAUUGGGAAGGAAU |
|  |  | gma-miR482d-3p | UCUUCCCUACACCUCCCAUACC |
| gma-MIR482e | AGACAAAAUUGGGGGAAGGCAUGGGUAUGGGGGGAUUGGGAAGGAAUAUCCAUAAGCAAA AUAUGCUAUUUCUUCCCUACACCUCCCAUACCACUGUUUUUCCUCUCUUUCUCCCCC | gma-miR482e | UAUGGGGGGAUUGGGAAGGAA |
| gra-MIR482 | GAAGGAGGAAAAGGAGAGACCGUUUCUGGAAAUUUUCGGAAUGGAGGAGUUGGAAAGAUUUUGAAUUUUCUCUGUAUUUUAAUAAUGAAAAAUCUUUCCAAUUCCUCCCAUUCCACUGAUUUCCAGCACUUCCUCUCCAUUUUCUUACCUUU | gra-miR482 | UCUUUCCAAUUCCUCCCAUUCC |
| gra-MIR482c | UCUGGAAAUUAUAGGAAUCGCGUUUUUGGGAAGAAGCACGAUCGAUCUGCAUCCGCCACUAAUUUCUUCCCAAAACCUCCAAUUCCAAUGAUUUCCAG | gra-miR482c | UUCCCAAAACCUCCAAUUCCAA |
| gra-MIR482d | ACUGGAAAUUUUUGGAAGUGGGGUGCGAGGAAAGAUCAUUUCUCUUCUCUCUUAUAUAUAUACACACACACACACACACACACAUACAAAGAGAUCAAACCAGAUCUUUCCUAUGCCCCCCAUUCCACUGAUUUCUAG | gra-miR482d | UUUCCUAUGCCCCCCAUUCCAC |
| gso-MIR482a | GGGGAAGGCAUGGGUAUGGGGGGAUUGGGAAGGAAUAUCCAUAAGCAAAAUAUGCUAUUUCUUCCCUACACCUCCCAUACCACUGUUUUUCCU | gso-miR482a | UCUUCCCUACACCUCCCAUAC |
| gso-MIR482b | GGGGAAGACAUGGGUAUGGGGGGAUUGGGAAGGAAUAUUCAUAAGCAAAAUAUGUUAUUUCUUCCCUACACCUCCCAUACCACUGUUUUUCCU | gso-miR482b | UCUUCCCUACACCUCCCAUAC |
| hbr-MIR482a | GGUUAGAGGAAGUUCAUGGAGAUGGGUGGCUGGGCAAGAAGAAAAAGAUGUUUUGUCUAAUCGUGGGUCAUCUUCUUGCCUACUCCACCCAUUCCAUCCACUUCCUUGAACUU | hbr-miR482a | AGAUGGGUGGCUGGGCAAGAAG |
| hbr-MIR482b | AGAGCUACUGGAAGUUGUAGGAAUGGGCGGUUUGGGAAAGAAUUUUAUAUACGAUAAGAGAAUUAAUUAUAUAUAUGUUAUUAUUCUCUUGAAUAAGUGUAUAAAUGUUUCUUCCCAAUGUCGCCCAUUCCGAUGAUUUCCAGAAGCUCC | hbr-miR482b | GAAUGGGCGGUUUGGGAAAGA |
| mdm-MIR482a | GAGAAGAGGGAAAGGGAGAUUGGAGCUGCUGGAAGUUUUAGGAAUGGGCUGUUUGGGAAGAARGAAAAUUACAAGAAUAAUUGUCUUGUGGGGUUUCUUCCCAAGCCCGCCCAUUCCUAUGAUUUCCAGCUGUUCCUCCCUUUCCCUUGUAUCUC | mdm-miR482a-5p | AGGAAUGGGCUGUUUGGGAAGA |
|  |  | mdm-miR482a-3p | UUCCCAAGCCCGCCCAUUCCUA |
| mdm-MIR482b | AGGAAGUUGUUGGAAUGGAAGGGUAGGAAAGAAGAAACUUCUCUUGUAUGUUCCAAAUUCAAGAGAUAAAAUUUCUUUCCUAUCCCUCCCAUUCCUUCKAUUUUCU | mdm-miR482b | UCUUUCCUAUCCCUCCCAUUCC |
| mdm-MIR482c | GGAAUAGGAAGGCUUUGAGGAGGUUUAUGGAGUGGAAGGGUAGGAAAGAAGUAACUUCCUUUUURGGUCUCAAAUUCUGGAGAUCGAAUUUCUUUCCUAACCCUCCCAUUCCUUCAAUUUCCUAAAGRUUAUCCCUUUUCC | mdm-miR482c | UCUUUCCUAACCCUCCCAUUCC |
| mdm-MIR482d | AGGAAGUUGUUGGAAUGGAAGGGUAGGAAAGAAGAAACUUCUCUUGUAUGUUCCAAAUUCAAGAGAUAAAAUUUCUUUCCUAUCCCUCCCAUUCCUUCKAUUUUCU | mdm-miR482d | AAUGGAAGGGUAGGAAAGAAG |
| mes-MIR482 | GGAGAAAAAGCAAGGAUUUUCGGAAGGUUAUGAGAUGGGUAAGUGGGGAAGAUAACUGUGGGGCUUUCGUUUUGUUUGUUAUCUUCCCUACUCCACCCAUUCCAUAGCUUUCCGAUCGUUCCUCUCUCUCC | mes-miR482 | UCUUCCCUACUCCACCCAUUCC |
| mes-MIR482b | AGAUAGAGAGCUACUGGAAGUUGUAGGAAUGGGCGGUUUGGGAAAAAAUCAUAUACAUAAAUACGAUAACAGAUUACGUCGAAAAUCAAAUUAAGUGUAUACAAGUUUUCUUCCCAAUGUCGCCCAUUCCGAUGAUUUCCAGAAGCUCCUCCCUUUC | mes-miR482b | UUCCCAAUGUCGCCCAUUCCGA |
| mes-MIR482c | GAGAGGGGUUGCUGGGAGCUAUGGGUAUGGGGGGAUUGGGCAAAAUUGCUUGGCUUUGUUUAUUAGCAGAGGCUAAAGCUGAUUUUUCCCAAGACCUCCCAUACCAGUGGCCUCCAGCUGUUUCCUCCCC | mes-miR482c | UUUUCCCAAGACCUCCCAUACC |
| mes-MIR482d | AGAGAGUGCUAGAGGAAGUUAAUGGAGAUGGGUGGCUCGGGAAGAAGAAAAAGAGGCCUAGUCUUCUUCCCGACACCACCCAUUCCAUCCACUUCCUCCAACUUCUCUCUUUC | mes-miR482d | UUCCCGACACCACCCAUUCCAU |
| mes-MIR482e | AGUUAGGAUUUUCAGAAGGUUGCGGGAUGGGUGGGUGGGUGAGAUGACCAAUUAAUGGAUCUCAAUUGAUUGUGUUCAUCUUACCUACACCGCCCAUGCCUUGGGUUUCCGAUUAUUCCUCCCUC | mes-miR482e | UCUUACCUACACCGCCCAUGCC |
| mtr-MIR482 | GGGGAAGGCAUAGGCAUGGGAUAGUAGGGAAGAAGUGAUAACAGCAUAUGCUUAUUAUGGUGCUUCUUCUUACCUACACCUCCCAUGCCUACGUUUUCCCUCUA | mtr-miR482-5p | GGCAUGGGAUAGUAGGGAAGA |
|  |  | mtr-miR482-3p | CUUACCUACACCUCCCAUGCC |
| nta-MIR482a | AGUUUAUCCGGAAAACUACGGGGAUUGGUGGGUUGGAAAGCUUUUCACUUCUGAUUAAACGCUUUCCAAUUCCACCCAUUCCUAUGGUUUUUCAAUAAUCUCUC | nta-miR482a | UUUCCAAUUCCACCCAUUCCUA |
| nta-MIR482b | GUGUUUAAGGGAGCUUUUGGAGUGGGUGGAGUGGUAAGAUAUCAUAUUUAUGUUGAAUCAUAAUAAUUGAAAUCUUGCCAAUGCCAUCCAUUCCUAUGGCUUCCAAUCAUUUCU | nta-miR482b-5p | AGUGGGUGGAGUGGUAAGAUA |
|  |  | nta-miR482b-3p | UCUUGCCAAUGCCAUCCAUUCC |
| nta-MIR482c | GAAAACUAUGGGGAUUGGUGGGUUGGAAAGCUUUUCCCUUCUGAUUGAUUAAACGCUUUCCAAUUCCACCCAUUCCUAUGGUUUUUCA | nta-miR482c | UUUCCAAUUCCACCCAUUCCUA |
| nta-MIR482d | GGGAGCUGUUGGGGUGGGGGGCGUGGGAAGAUGUCAUCUUUACUUUUCCUGCGAAGGAGAUUGGCAUCUUCCCGACUCCCCCCAUACCACUGGCUUCCAA | nta-miR482d | UUCCCGACUCCCCCCAUACCAC |
| pab-MIR482a | UUGGAGGAGUGGAAGGAUAGGGUAAGACUGCGGUUAAGUGAGACUGCUUCAAUAAUUCUUUGCUCUAAUCUCACCAGUCUUCCCUACUCCUCCCAUUCCUGUUGCCUUCAUCACAGUU | pab-miR482a | UCUUCCCUACUCCUCCCAUUCC |
| pab-MIR482b | AUGUGGAUGGAAGUCUUGAGGAUUGGGAGGAUAGGAGAAGGCUGUUUGCUUAAUUAUCAGUUGAAACCUGAUUAGUCUUCCCUAUUCCUCCCAUUCCUAUUGCCUCCAUCACACAUCCCUU | pab-miR482b | UCUUCCCUAUUCCUCCCAUUCC |
| pab-MIR482c | UGAGAAGGGAAUGGAUGUGUUUUGUGGAUGGAAGUCUUGAGGAGUGGGAGCAGAGGAUAAGACUGCUGCUUUGAAUAUUACCAGUCUUUCCUACUCCUCCCAUUCCUAUUGCCUUCACCACACAUCCCUUCCCAACG | pab-miR482c | UCUUUCCUACUCCUCCCAUUCC |
| pab-MIR482d | AAGGCCAAAGGCUUGCGAGGAUAGGAAAAACUCGUGUGGUGUUGUAUUUUCUCACUCACCGAUCUACAGUUUUUCCCACUCCUCCCAAGCCCAUGGCCUU | pab-miR482d-5p | GGCUUGCGAGGAUAGGAAAAA |
|  |  | pab-miR482d-3p | UUUCCCACUCCUCCCAAGCCCA |
| pab-MIR482e | GGAGUAGUGGAAAUGGGUGAAGUCAAAAGACCUCUUUCCAUUGUAGAUAUGGCGGUCUUUCCACUUCUACCCAUUUCCGCUAUUUC | pab-miR482e | UCUUUCCACUUCUACCCAUUUC |
| pab-MIR482f | UAGUGGAAAUGGGUGAAGUCGAAAGGCCUCUUUCCAUGGCGGUCUUUCCACUUCUACCCAUUUCCGCUA | pab-miR482f | UCUUUCCACUUCUACCCAUUUC |
| pab-MIR482g | GGAGUAGCCGAAAUGGGUAGAUUGGAAAUACCUCUGCCCGAUUCUUCUCAUGGAUAUGGUGGUCUUUUCAGUUCUACCCAUUUCAGCUAUUCC | pab-miR482g | UCUUUUCAGUUCUACCCAUUUC |
| pab-MIR482h | CUUAUGGGAGUAGUCGAAAUGGGUAGAUUGGAAUGACCUCUGCCUGAUUCUUCAUAUGGAUGUGGCGGUCUUUUCAGUUCUACCCAUUUCCGCUAUUCCACUAAG | pab-miR482h | AAUGGGUAGAUUGGAAUGACC |
| pab-MIR482i | AGGCCUUAGGCUUGCGAGGAUGGGAAAAACGCAUACGGUGUUCCUUCACCGAUUUAUAGUCUUUCCUACUCCUCCCAAGCCCAUGGCCU | pab-miR482i | UUUCCUACUCCUCCCAAGCCCA |
| pab-MIR482j | GGUCUUUAGGCUUUGGAGGAUUUGGAAAGGCAUAGUCAUUAUUGUUACCGAGGAUCAUCGAUUUUCUCUGAUCCUUUCCAACGCCUCCCAUGCCUAUAGUCC | pab-miR482j | UUUCCAACGCCUCCCAUGCCUA |
| pab-MIR482k | AUGGGUAUGGGAGGAAUGGGCAAAGCUCACUGCUCAGAGCUCAAGGAAUGAGUUGUUUGAGUCUUCCCUAAACCUCCCAAACCCAU | pab-miR482k | UCUUCCCUAAACCUCCCAAACC |
| pab-MIR482l | GGGUAGAAAGGCCUUAGGCUUGCGAGGAUGGGAAAAAUUCAUACGGUGUUCACUCAUUGUUUUGCAGUUUUUCCUACACCUCCCAACCCCAUGGUCUUGUCUUACCU | pab-miR482l | UUUUUCCUACACCUCCCAACCC |
| pab-MIR482m | UGUAGUUUUAAGGCCACUGGGUCUGGGAGGAGAGGAAAAACCCAUGCAGUCACUCAUGAUUUCAUCUGAUCUGAUGUGAUAUAGGCAUUGUGACAGUUUUUCCAACUCCACCUAGACCUAUUGCCUUCACUACA | pab-miR482m | UUUUUCCAACUCCACCUAGACC |
| pab-MIR482n | UAGUUUUAAGGCCAUUAGGUCUCGGAGAGCAGGGAAAAACCAGGCAGUAACUCAUGAUCCCAUGUGAUUUAUGAACUGUUACAGUUUUCCCCACUCCACCGAGACCUAUGGCCUUCACUA | pab-miR482n | UUUUCCCCACUCCACCGAGACC |
| pab-MIR482o | ACAGGGAUGCUGUGCAGUGUGGGGCAUUAGGCCUGGGACGAUUGGAAAGACUUGGUCUAUUUUCUCCGCUUAGUCUUCCCAACCCCUCCCAUGCCUAUAUUCCCCACUGCACAUCCCUGU | pab-miR482o | UCUUCCCAACCCCUCCCAUGCC |
| pab-MIR482p | GGGAUUUUGUGGUUUGAGGGCAUUGGGAGUGGGCGGAACGGGCAAAACUCAGUCAGUUCGUUCAUUCGUUAGUGAACCCAUCCAAUCUGAUCGGGUUUUCCCGGCUCCGCCCAUUCCUACAGUCCUCACUACGCAUCCC | pab-miR482p | UUUUCCCGGCUCCGCCCAUUCC |
| pab-MIR482q | GUUACAGACGGAAGCUCUACAAGUUCGCGUGAUUAGUGUGUAUGGAGUGGCCACGCUGGAAAAAUAUUGUUGCUCAAAAUGAGCUACAAUCUCUAGAAAGUUAUUGAUGUUUCUGAUGUGGGUCACAGUCUCUUGAGAUUGUUGAGAGGAAAAAUCAAUUUAGAUAUGGAUGAAAAUUUCAUAUUAUAUUUAAAUCGAUUUUUGAAUAACAAUUUUGAGAAACUGUAAGCCAAAUCACAAUAUCACAAGUCACUGACUUUGUAGGUGUUGUAGAUUGUUUUGAGCAACGAUGUCUUUCCAACGCCGGCCAUUCCAUAUACACCAAUCACCCCAACUUGUAGAGUUUCAGCGAAC | pab-miR482q | UCUUUCCAACGCCGGCCAUUCC |
| pab-MIR482r | UGGCAACUCUCGUUCGCGGAAACUCUACAAGUUCGCGUGAUUGGUGUGUAUGGAGUGGCCCCGUUGGAAAAAUCUUGUUGCUCGAUAUUAUCUACAUUAACUACAAGUGAUUGAUGCGUCUGAUAAUGUGAUUUAGUCAGAGUGUCUCAAGUUUGUUGAGGGAAAAAUAAAUUAGAGAUGGUUGAAAUUCAGUUUACAGAGUAAACCAUUUCAUUCAUAUCUAAGUUGAUUCGCUGAGCAAAUUGAGAAAUUAUGAGCCAAAUCACAAUAUCAUGCAUACAUCACUGACCUUGUAAGUGUUGUAUUGUAGACUAUUUUGAGCAACGAUGUCUUUCCAACGCCGCCCAUUCCAUAUACACCAAUCACGCUAAUUUGUGGAGUUUCAGCGAACAAAGAGUAUUCA | pab-miR482r | UCUUUCCAACGCCGCCCAUUCC |
| pab-MIR482s | UGGCAACUCUCGUUAGCUGAAACUCUACAAGUUUGCGUGAUUGGUGUGUAUGGAGUGGCCCCGUUGGAAAAAUCUUGUUGCUCGAUAUUAUCUACAUUGACUACAAAGUGAUUGAUGCGUCUUGAUACUCUGAUUUGGUCACAGUCUCUCAAGUUUGUUGAGGGAAAAAUAACUUAGAGAUGGAUGAAAUUCAGUUUACAGAGUAAAAUUUUUUAUUCAUAUCUAAGUUGAUUCCUUGAACAAUUUUGAGAGACUGUGAGCCAAAUCACAAUAUCAUGCAUACAUCACUGACCUUGUAAGUGUUGUAUUGUAGACUAUUUUGAGCAACGAUGUCUUUCCAACGCCGCCCAUUCCAUAUACACCGAUCACGCCUACUUGUGGGGUUUCAGCUAACAAAGAGUAUUCA | pab-miR482s | UCUUUCCAACGCCGCCCAUUCC |
| pab-MIR482t | UCCUUAUGGGAGUAGUGGAAAUGGGUGAAGUCGAAAGACCUCUUUCCAGGGCGGUCUUUCCACUUCUACCCAUUUCCGCUAUUUCACUAAGUGA | pab-miR482t | UCUUUCCACUUCUACCCAUUUC |
| pab-MIR482u | GGAGUAGUGGAAAUGGGUGAAGUGGAAAGACCUCUUUCCAAUGUGGAUAUGGCGGUCUUUCCACUUCUACCCAUUUCCGCUACUUC | pab-miR482u | UCUUUCCACUUCUACCCAUUUC |
| pab-MIR482v | CUUAUGGGAGUAGUGGAAAUGGGUAAAGUUGAAAGACCUCUUUCCAUUGUGGAUAGGGCGGUCUUUCCACUUCUACCCAUUUCCGCUAUUUCACUAAG | pab-miR482v | UCUUUCCACUUCUACCCAUUUC |
| pab-MIR482w | GUGAUUGAUCCGUCUGAUACUGUGAUUUGGUCACAGUCUCUCAAGUUUGUUGAGGGAAAAAUAACUUAGAGAUGGAUGAAAUUCAGUUUACAGACAUUUCAUUCAUAUCUAAGUUGAUUCGCUGAACAAUUUUGAGAAACUGUGAGCCAAAUCACAAUACCAUGCAUACAUCACUCAC | pab-miR482w | UUGAGAAACUGUGAGCCAAAUC |
| pab-MIR482x | AGUGAUUGAUGCGUCUAAUACUCUGAUUUGGUCAGAGUCUCUUAAGUUUGUUGAGGGAUUCAUAUCUAAGUUGAUUCCCUGAACAAUUUUGAGAAACUGUGAGCCAAAUCACAAUAUCAUGCAUACAUCACU | pab-miR482x | UUGAGAAACUGUGAGCCAAAUC |
| pde-MIR482a | UGAGAAGUGAAGGGAUGUGUUUUGUGGAUGGGAGUCUUGAGGAGUGGGAGCAUAGGAUAAGGCUGCUUCAUAUCACCAGUCUUUCCUACUCCUCCCAUUCCUAUUGCCUUCACCACACAUCCCUUCCCAA | pde-miR482a | UCCUCCCUACUCCUCCCAUU |
| pde-MIR482b | AUGUGGAUGGAAGUCUUGAGGAGUGGGAGGGUAGGAGAAGGCUCUGUGGUGAGGUUUCAGUCAUAAUCUCAUCAGUCUUCCCUAUUCCUCCCAUUCCUAUUGCCUCCCUUCACAAUGUAAC | pde-miR482b | UCUUCCCUAUUCCUCCCAUUCC |
| pde-MIR482c | AAGGCCAAUGGCUUGCGAGGGUAGGAAAAGCUCAGUGUGAUGAUAUAUUUCUCGCUCACUGAUCUGCAGUUUUUCCCACUCCUCCCAAGCCCAUGGCC | pde-miR482c | GGCUUGCGAGGGUAGGAAAAG |
| pde-MIR482d | GUCAUGGGGUCUUUAGGCUUUGGAGGAUUUGGAAAGGCUUAGUCAUUCUUUUUACCGAGGAUAUUCGAUUUUCUAAGAACCUUUCCAACGCCUCCCAUGCCUAUAGUCCUCAUUGUACAUGUCACCCGAUCGCAG | pde-miR482d | GGCUUGCGAGGGUAGGAAAAG |
| pgi-MIR482a | GAGAUGGGUCUUAUGAAGGCAGAGGUAGUGGGAGGCUUAGCAAGAAAAAUAAACAACUUCAACUGGCUGUUUUUCUUGCCAAUUCCUCCCAUUCCAAUGGUUUCAUGAGGUUACUC | pgi-miR482a | UCUUGCCAAUUCCUCCCAUUCC |
| pgi-MIR482b | AUUUUUCUUGCCAAUUCCUCCCAUUCCAAUGGUUUCAUGAGGUUACUCCUUUUCCUAUCAUAUCCUUGUAACCCAACACCCCCAUAACGUGUUCGACAGAGAGAGAGGGAAAGGGAGAUAGAUCUAGAUGAAGUUGUUUGGAUGGGUGAUUUGGAAAGAAAAGU | pgi-miR482b | UCUUGCCAAUUCCUCCCAUUCC |
| ppe-MIR482a | AAGAGGAAAUGGAAAUUUUUGGGUGAGAGGUUGCCGGAAAGAAUACAUAAUAUUCAUCCAAGUUUUGAUAUUAAAUUCUUUCCGAAACCUCCCAUUCCAAUGAUUUCCAGCUAUUCCUUU | ppe-miR482a-5p | GGGUGAGAGGUUGCCGGAAAGA |
|  |  | ppe-miR482a-3p | UUUCCGAAACCUCCCAUUCCAA |
| ppe-MIR482b | AGUUGCAGUAGGGAGUUAGGGGAAUGGGAGGAUUGGGAAAAACAUGCACCCCAAGUGUUCGUGAAUAUUACACAAGGAACACAAAAAAGGACUUCUUCCCAAACCUCCCAUUCCUAUUAUUUCCUACAGUUGCUCU | ppe-miR482b-5p | GGAAUGGGAGGAUUGGGAAAA |
|  |  | ppe-miR482b-3p | CUUCCCAAACCUCCCAUUCCUA |
| ppe-MIR482c | UGGAGCUACUGGGAAUUGUAGGAAUGGGCUGUUUGGGAUGAAAGAAAAUCACUAAGAAAAUUUUCUUGUUGAUAUUUUCUUCCCAAGCCCGCCCAUUCCAAUGAUUUCUAGCGGCUCCUCC | ppe-miR482c-5p | GGAAUGGGCUGUUUGGGAUG |
|  |  | ppe-miR482c-3p | UUCCCAAGCCCGCCCAUUCCAA |
| ppe-MIR482d | AGGCCUCCCAGAGGCCAAUGGAGAUGGGUGGCUGGGAAGGAUCCUCAUAAUUUCUCUACAUUUUAUUAAUUGAAAACUAAGGAGGUGACAAAAUAGGGGCUCCCUCCCAUGCCACGCAUUUCUAUUGUUUCUGGGUUUCUCUCC | ppe-miR482d-5p | GAGAUGGGUGGCUGGGAAGGA |
|  |  | ppe-miR482d-3p | CCUCCCAUGCCACGCAUUUCUA |
| ppe-MIR482e | GAGGAAGUUUUUGGCCAUGGGAAGUUUGGCAAGAAAUAUUUAUUAUAUAUUCUACUACUUCAAGUUCUGUUUGAAGUAUGUAUGUAUAAGUAGUUCUUGCCUAUUCCUCCCAUGCCAAUGAUUUCCUA | ppe-miR482e | UUGCCUAUUCCUCCCAUGCCAA |
| ppe-MIR482f | GGUGGGGAUAGGAAGGGCUUGAGGAUGCUGUUGGAGUGGGAGAGUGGGAAAAGAAUGGAUCUCUUUGGUUGGUUCAAAGGGAACAAAUUUCUUUCCUACUCCACCCAUUCCUGCAAUUUCCUUAGGUUUCUCCCUUUCCCUUUCC | ppe-miR482f | UCUUUCCUACUCCACCCAUUCC |
| pta-MIR482a | GAGGUGUGGAAGGAUAGGGUAAGACUAAGGUUAAGGAGAAACUCUGCUUCAUUAAUUCUCUGCUCCAGUCUUCCCUACUCCUCCCAUUCCUU | pta-miR482a | UCUUCCCUACUCCUCCCAUUCC |
| pta-MIR482b | GAGGUGUGGAAGGAUAGGGUAAGACUAGGGUUAAGCAGAAACUCUGCUUCAUUAAUUCUCUGCUCCAGUCUUCCCUACUCCUCCCAUUCCUU | pta-miR482b | UCUUCCCUACUCCUCCCAUUCC |
| pta-MIR482c | AGGAGUGGGAGGGUAGGAGAAGGCUCUGUGGUGAGGUCUCAGUCAUAAUCUCAUCAGUCUUCCCUAUUCCUCCCAUUCCU | pta-miR482c | UCUUCCCUAUUCCUCCCAUU |
| pta-MIR482d | GAGGUGUGGAAGGAUAGGGUAAGACUAAGGUUAAGGAGAAACUCUGCUUCAUUAAUUCUCUGCUCCAGUCCUCCCUACUCCUCCCAUUCCUU | pta-miR482d | UCCUCCCUACUCCUCCCAUU |
| ptc-MIR482a | GAGUCCUAGCAAGUCUUUGGAGAUGGGAGAGUAUGCAAGAAGGAAAAAUUCAUGAUUUAAUAUUCCUUCUUGCCUACUCCUCCCAUUCCAUCUGCUUUCUGCGACUC | ptc-miR482a.2 | UCUUGCCUACUCCUCCCAUU |
|  |  | ptc-miR482a.1 | CCUACUCCUCCCAUUCC |
| ptc-MIR482b | GAGAAGAGCCGGAGGGGGAGACUGAGACCUACUGGAAGUUGUGGGCAUGAGGUGUUUGGCAAGAAAAUGGAUCUUUUCCUUAUGAUGAUUUCUUACCAAUACCUCUCAUGCCAAUGAUUUCCAGCAGUUCCUCCCUUUCCCUUCUUUCUC | ptc-miR482b-5p | GGCAUGAGGUGUUUGGCAAGA |
|  |  | ptc-miR482b-3p | UUACCAAUACCUCUCAUGCCAA |
| ptc-MIR482c | UCCUCCGCCGUGUUGGAAAGGAAAGGGAGACUGCUUUACUGGAAGCUUCCGGUAUGGGAGAGGCGGGAAUGACUCUGAAAGAACUUAGAAGAGUCUUUCCGAGUCCUCCCAUACCGCAGAUUUCCAGUAGUUCCUCCCUUUACUUAUCUUCACUAUUCCAGCAGCUCUGGA | ptc-miR482c-5p | UAUGGGAGAGGCGGGAAUGACU |
|  |  | ptc-miR482c-3p | UCUUUCCGAGUCCUCCCAUACC |
| ptc-MIR482d | AGAGAAGAGGGGAAAGAGAUGGAGGACGAGGAAGUUUCUGGACAUGGGUUGGUUUGCAAGAAAAUAAGAUUUUUGAACUCUCUGAUAUUGUUCAUGGAAAACAGUUUUCUUGCCGACCCCACCCAUGCCAAUGAUUUCCUCAGCUUCCUCUCUCUCCCUGUUUCCUUCUCU | ptc-miR482d-5p | GGACAUGGGUUGGUUUGCAAGA |
|  |  | ptc-miR482d-3p | UUGCCGACCCCACCCAUGCCAA |
| pvu-MIR482 | UUGUGGGAAUGGGCUGAUUGGGAAGCAAUGAAAACUCAAGCAAUUGUGUCUACAUUUUCUUCCCAAUUCCGCCCAUUCCUAUGA | pvu-miR482-5p | GGAAUGGGCUGAUUGGGAAGCA |
|  |  | pvu-miR482-3p | UCUUCCCAAUUCCGCCCAUUCC |
| sly-MIR482a | CGAAAAUCUAUGGGGAUUGGUGAGUUGGAAAGCUUUUCUUUUUCUUCUCUUGUGAUUAGCUUUCCAAUUCCACCCAUUCCUAUGGUUUUUCG | sly-miR482a | UUUCCAAUUCCACCCAUUCCUA |
| sly-MIR482b | GGAAAGGGAUGAAGAUUUUUGGGAAGCUUUUGGCGUGAGCGGUGGGGAAAGAUAGAUCUAUCAUAUUAGUAUGAUAUGAUAAAUCUUGCCUACACCGCCCAUGCCCAUGGCUUCCAAUGAUUUCU | sly-miR482b | UCUUGCCUACACCGCCCAUGCC |
| sly-MIR482c | GUGUUUCGAGGAGGUGUUGGAGUGGGUGGUGUGGUAAGAUUUCAUAUUUAUUUUCCAUAAAAAUUGAGAUCUUGCCAAUACCGCCCAUUCCUAUGGCUUCUGAUCAUUUCU | sly-miR482d | UCUUGCCAAUACCGCCCAUUCC |
| sly-MIR482d | GGAAAGGGAGGAGGGAUUUGGGAAUCUUUUGGAGUGGGUGGGAUGGAAAAAUAUUGUAUUUUUUUUUCUAACGGAAAAAAAGAUGAGAAUUGAGAUUUUUCCUAUUCCACCCAUGCCAAUGGCUUCCAGUCAUUUACUCCUUUUCC | sly-miR482d-5p | GGAGUGGGUGGGAUGGAAAAA |
|  |  | sly-miR482d-3p | UUUCCUAUUCCACCCAUGCCAA |
| sly-MIR482e | GAUCUGUGCUAUGGAAAUAUUGAUGGCGUUGAAAGGGAUGAGAGUAUUGGGAAGUUGUUGGUUGUGGGUGGGGUGGAAAGAUUUGAUAAAUCUAAUUUUAAAGAGAUUAAAUCUUUCCUACUCCUCCCAUACCCAUGACUUCCACUAGCUUCUCCCUUUCUUCAUUUGUCAUCUCCUUUGCUAAGUUC | sly-miR482e-5p | UGUGGGUGGGGUGGAAAGAUU |
|  |  | sly-miR482e-3p | UCUUUCCUACUCCUCCCAUACC |
| stu-MIR482a | GAGUUUUUCGAAAAUCUAUGGGAAUUGGUGGAUUGGAAAGCUUUUAUUUUCUUCUGAUUAGCUUUCCAAUUCCACCCAUUCCUAUGGUUUUUCGGUAUCCUCUC | stu-miR482a-5p | GGAAUUGGUGGAUUGGAAAGC |
|  |  | stu-miR482a-3p | UUUCCAAUUCCACCCAUUCCUA |
| stu-MIR482b | GAAUGUUUCAGGGAGCUUUUGGAGUGGGUGGCAUGGUAAGAUAUCAUAUUUUGUUUUUCAUAAAAAAUGGAAUUGAAAUCUUACCGAUUCCCCCCAUUCCAAUGGCUUCCAGUCAUUUCUCC | stu-miR482b-5p | GGAGUGGGUGGCAUGGUAAGA |
|  |  | stu-miR482b-3p | UUACCGAUUCCCCCCAUUCCAA |
| stu-MIR482c | GAGAGAUUUGGGAAGCUUUUGGAGUGGGUGGGAUGGAAAAAUAUCGUAUUUAUUUUUUUUCCUUAAAAGGCAAAAAAGAUAAGAAAUGAGAUUUUUCCUAUUCCACCCAUGCCAAUGGCUUCCAAUCAUUUACUC | stu-miR482c | UUUCCUAUUCCACCCAUGCCAA |
| stu-MIR482d | AAUUUUUGGGAAGCUUUUGGCGUGAGUGGUGGGGUAAGAUAGAUCUAUCAUAUUUAUGAUAUGAAAAAUCUUGCCUACACCGCCCAUGCCCAUAUCUUCCAAUUAUUUCU | stu-miR482d-5p | CGUGAGUGGUGGGGUAAGAUA |
|  |  | stu-miR482d-3p | UCUUGCCUACACCGCCCAUGCC |
| stu-MIR482e | GUGUUUCGGGGAGGUGUUGGAGUGGGUGGUGUGGUAAGAUUUCAUAUUUAUUUUCCAUAAAAAUUGAGAUCUUGCCAAUACCGCCCAUUCCUAUGGCUUCCGAUCAUUUCU | stu-miR482e-5p | AGUGGGUGGUGUGGUAAGAUU |
|  |  | stu-miR482e-3p | UCUUGCCAAUACCGCCCAUUCC |
| vun-MIR482 | UCAGAAAUUGUGGGAAUGGGCUGAUUGGGAAGCAAUGAAAUCAUCUCAAGUGAUUGUGCGUAAUUUUCUUCCCAAUUCCGCCCAUUCCUAUGAUUUCUGA | vun-miR482 | UCUUCCCAAUUCCGCCCAUUCCUA |
| vvi-MIR482 | AAUGUUUGGGAAUUGGAGAGUAGGAAAGCUUAGCCAUCUAUUCCCUUCAUGGUUUCCUCUCCAUGGAUUGGGGGUCUAGAGCUAGUCUUUCCUACUCCUCCCAUUCCUAUUGUUUUC | vvi-miR482 | UCUUUCCUACUCCUCCCAUUCC |
| zma-MIR482 | AGUGGGAGAUGAAGGAGCCUUGCAUCGAUGUCACCGCCGGAGGAGCGCUCGCCUUCUUCGCGCACCGCCGCAAUAGCCGCCCUCGGACCCCUCGCCUCGCUCUUCCUUGUUCCUCCCAUUUU | zma-miR482-5p | UGGGAGAUGAAGGAGCCUU |
|  |  | zma-miR482-5p | UCUUCCUUGUUCCUCCCAUU |
