# Supplementary material for: Analysis of the miR482 Gene Family in Plants
Source: Genes (Basel). 2024 Aug 8;15(8):1043. doi: 10.3390/genes15081043 (PMC11353999; doi:10.3390/genes15081043)
Supplement: Supplementary file 1 [file genes-15-01043-s001.zip › Supplementary Materials/Supplementary Materials-Table S3.docx]

**Supplementary Table S3. The ceRNAs network of miR482.**

| **Species** | **LncRNA/CircRNA** | **MiRNA** | **MRNA** | **Function annotation of Mrna** |
| --- | --- | --- | --- | --- |
| Cotton | TCONS_00003967 | miR482g | VOZ1 | Transcription factor VOZ1 |
|  |  |  | MDIS2 | Protein MALE DISCOVERER 2 |
|  |  |  | KCS1 | 3-ketoacyl-CoA synthase 1 |
|  |  |  | EXPA5 | Expansin-A5 |
|  |  |  | EXPA5 | Expansin-A5 |
|  |  |  | RER4 | Protein RETICULATA-RELATED 4, chloroplastic |
|  |  |  | APFP | Aspartyl protease family protein At5g10770 |
|  |  |  | ABF2 | Acid-insensitive 5-like protein 5 |
| Tomato | LncRNA15492 | miR482a | NBS-LRR | Nucleotide-binding site-leucine-rich repeat |
|  | LncRNA23468 | miR482b |  |  |
|  | LncRNA08489 | miR482e-3p |  |  |
|  | LncRNA39298 | miR482e-5p |  |  |
|  | TCONS-00026121, TCONS-00061862, TCONS-00026124, TCONS-00061875, TCONS-00061867 | miR482c-3p | CC-NBS-LRR | CNL (CC-NBS-LRR) |
|  |  |  |  |  |
|  |  |  |  |  |
|  |  |  |  |  |
|  |  |  |  |  |
|  | TCONS-00057529, TCONS-00113349, TCONS-00057528 | miR482e-3p | CC-NBS-LRR | CNL (CC-NBS-LRR) |
|  |  |  |  |  |
|  |  |  |  |  |
| Peanut | MSTRG.2115, MSTRG.30601, MSTRG.30599, MSTRG.31962, CircRNA320 | miR482c-p5 | NEIN3W | Mediator of RNA polymerase II transcription subunit 15-like isoform X1 |
|  |  |  | CTM7LX | Uncharacterized protein LOC112802719 |
|  |  |  | Z9NEHU | Hypothetical protein Ahy_B09g094556 |
|  |  |  | X5NWFC | Uncharacterized protein LOC107643774 |
